# Metabolic Dysregulation of the Lysophospholipid/Autotaxin Axis in the Chromosome 9p21 Gene SNP rs10757274

**DOI:** 10.1161/CIRCGEN.119.002806

**Published:** 2020-05-12

**Authors:** Sven W. Meckelmann, Jade I. Hawksworth, Daniel White, Robert Andrews, Patricia Rodrigues, Anne O’Connor, Jorge Alvarez-Jarreta, Victoria J. Tyrrell, Christine Hinz, You Zhou, Julie Williams, Maceler Aldrovandi, William J. Watkins, Adam J. Engler, Valentina Lo Sardo, David A. Slatter, Stuart M. Allen, Jay Acharya, Jacquie Mitchell, Jackie Cooper, Junken Aoki, Kuniyuki Kano, Steve E. Humphries, Valerie B. O’Donnell

**Affiliations:** 1 Division of Infection and Immunity, Systems Immunity Research Institute (S.W.M., J.I.H., D.W., R.A., P.R., A.O., J.A.-J., V.J.T., C.H., Y.Z., M.A., W.J.W., D.A.S., V.B.O.), Cardiff University, United Kingdom.; 2 Division of Neuropsychiatric Genetics and Genomics and Dementia Research Institute at Cardiff, School of Medicine (J.W.), Cardiff University, United Kingdom.; 3 School of Computer Science and Informatics (S.M.A.), Cardiff University, United Kingdom.; 4 Department of Bioengineering, University of San Diego, La Jolla, CA (A.J.E.).; 5 Applied Analytical Chemistry, Faculty of Chemistry, University of Duisburg-Essen, Essen, Germany (S.W.M.).; 6 Department of Cellular and Molecular Neuroscience and Dorris Neuroscience Center, The Scripps Research Institute, La Jolla, CA (V.L.S.).; 7 Cardiovascular Genetics, Institute of Cardiovascular Science, University College London, United Kingdom (J. Acharya, J.M., J.C., S.E.H.).; 8 School of Pharmaceutical Sciences, School of Pharmaceutical Sciences, Tohoku University, Sendai, Miyagi, Japan (J. Aoki, K.K.).

**Keywords:** atherosclerosis, lipids, mass spectrometry, phenotype, phospholipids

## Abstract

**Background:**

Common chromosome 9p21 single nucleotide polymorphisms (SNPs) increase coronary heart disease risk, independent of traditional lipid risk factors. However, lipids comprise large numbers of structurally related molecules not measured in traditional risk measurements, and many have inflammatory bioactivities. Here, we applied lipidomic and genomic approaches to 3 model systems to characterize lipid metabolic changes in common Chr9p21 SNPs, which confer ≈30% elevated coronary heart disease risk associated with altered expression of ANRIL, a long ncRNA.

**Methods:**

Untargeted and targeted lipidomics was applied to plasma from NPHSII (Northwick Park Heart Study II) homozygotes for AA or GG in rs10757274, followed by correlation and network analysis. To identify candidate genes, transcriptomic data from shRNA downregulation of ANRIL in HEK-293 cells was mined. Transcriptional data from vascular smooth muscle cells differentiated from induced pluripotent stem cells of individuals with/without Chr9p21 risk, nonrisk alleles, and corresponding knockout isogenic lines were next examined. Last, an in-silico analysis of miRNAs was conducted to identify how ANRIL might control lysoPL (lysophosphospholipid)/lysoPA (lysophosphatidic acid) genes.

**Results:**

Elevated risk GG correlated with reduced lysoPLs, lysoPA, and ATX (autotaxin). Five other risk SNPs did not show this phenotype. LysoPL-lysoPA interconversion was uncoupled from ATX in GG plasma, suggesting metabolic dysregulation. Significantly altered expression of several lysoPL/lysoPA metabolizing enzymes was found in HEK cells lacking ANRIL. In the vascular smooth muscle cells data set, the presence of risk alleles associated with altered expression of several lysoPL/lysoPA enzymes. Deletion of the risk locus reversed the expression of several lysoPL/lysoPA genes to nonrisk haplotype levels. Genes that were altered across both cell data sets were *DGKA, MBOAT2, PLPP1*, and *LPL.* The in-silico analysis identified 4 ANRIL-regulated miRNAs that control lysoPL genes as miR-186-3p, miR-34a-3p, miR-122-5p, and miR-34a-5p.

**Conclusions:**

A Chr9p21 risk SNP associates with complex alterations in immune-bioactive phospholipids and their metabolism. Lipid metabolites and genomic pathways associated with coronary heart disease pathogenesis in Chr9p21 and ANRIL-associated disease are demonstrated.

## Abstract 2

Supplemental Digital Content is available in the text.

The association of altered plasma lipids with coronary heart disease (CHD) risk has been known for decades, however, for some CHD-risk single nucleotide polymorphisms (SNPs), there is no association with traditional lipid measurements, such as lipoproteins (HDL [high-density lipoprotein] or LDL [low-density lipoprotein]) or their constituents: cholesteryl esters and triglycerides.^1^ As a prominent example, the relatively common *CDKN2A/2B* (rs10757274, A>G; minor allele frequency =0.48) SNP on chromosome 9p21 confers ≈30% elevated risk of CHD but acts independently of traditional lipid risk factors.^1^ Chr9p21 SNPs, including rs10757274, are believed to alter disease risk through modulation of the long noncoding (lnc)RNA, ANRIL, although both up and downregulation has been associated with risk (see discussion for more detail).^2,3^ ANRIL isoforms are detected in peripheral blood cells, aortic smooth muscle, endothelial cells, and heart, and SNPs in Chr9p21 are associated not only with CHD but also numerous cancers.^2,4–6^ Cellular studies show that ANRIL lncRNA downregulates the tumor suppressors *CDKN2A/2B* by epigenetic regulation, modulating expression of pathways involved in differentiation, apoptosis, matrix remodeling, proliferation, apoptosis, senescence, and inflammation.^5,7^ Whether or how the entire CHD-risk region or ANRIL regulates bioactive lipids is currently unknown.

Lipids represent thousands of diverse molecules. However, CHD clinical risk algorithms such as Framingham or QRISK include circulating lipoproteins only.^8,9^ Importantly, bioactive lipids that regulate vascular inflammation/proliferation in line with the function of ANRIL and thus may be directly relevant to Chr9p21-mediated CHD are not included in these measures. Indeed, whether ANRIL mediates its effects via an impact on bioactive lipid signaling has not been examined and was studied herein using lipidomics.

Here, plasma from a prospective cohort (NPHSII [Northwick Park Heart Study II]), which recruited ≈3000 men aged 50 to 64 years clinically free of CHD from 1990 to 1991, was analyzed using targeted and untargeted lipidomics, followed by validation, metabolic correlation, and network analysis.^10,11^ Then, gene transcription for lipid metabolic enzymes was mined in data from a cellular ANRIL knockdown study and from vascular smooth muscle cells differentiated from induced pluripotent stem cells obtained from individuals with/without Chr9p21 risk, nonrisk (NN) alleles, and corresponding isogenic lines deleted of the entire CHD locus.^12,13^ Database mining for potential candidate miRNAs linking ANRIL with gene expression was conducted. The study reveals novel insights into the potential role of key bioactive signaling lipids in this common but poorly understood form of CHD.

## Methods

The authors declare that all supporting data are available within the article (and its Data Supplement). Ethical approval for the use of NPHSII samples was provided by the National Hospital for Neurology and Neurosurgery and the Institute of Neurology Joint Research Ethics Committee, and Joint UCL/UCLH Committee of Human Research, Committees A and Alpha, and all samples were obtained with informed consent. Full methods are provided in Materials in the Data Supplement.

## Results

### Global Lipidomics Demonstrates That LysoPLs Are Reduced in GG Plasma Versus AA

**Figure 1.**
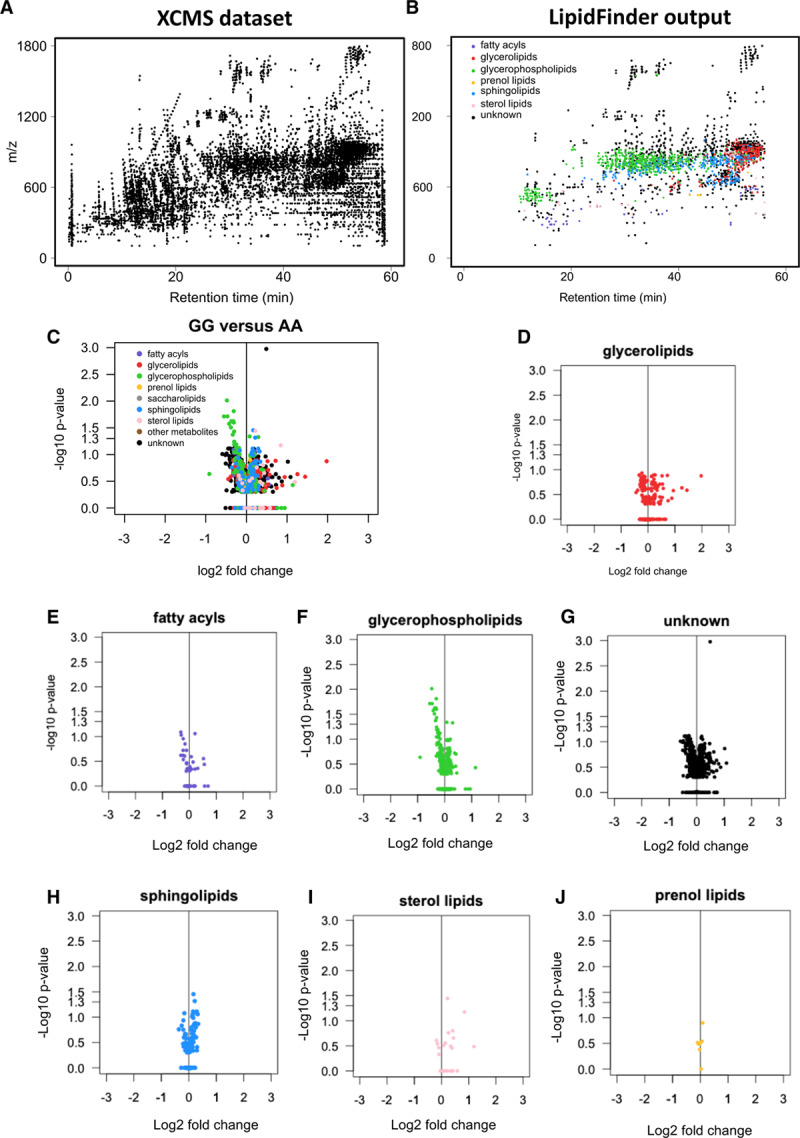
**Global lipidomics reveals class-specific changes in glycerophospholipids (GPLs) in rs10757274 GG vs AA.** **A**, Scatterplot of features in a plasma sample (≈14 000) after processing high-resolution MS data using XCMS. Analysis was undertaken using parameters provided in Materials in the Data Supplement. **B**, Scatterplot obtained after LipidFinder and manual-data clean-up, as described in Methods. Each dot represents a lipid described by m/z value and retention time. Putative identification and assignment of category were performed using WebSearch of the curated LIPID MAPS database. **C–J**, Volcano plots show differences in lipid classes with genotype. Volcano plots were generated as described in Methods, plotting log2(fold change) vs −log10(*P* value) for all (n=39 AA and 33 GG), following *P* value adjustment using sequential goodness of fit metatest (SGoF).

To capture all lipids (knowns/unknowns), high-resolution Orbitrap MS data from long chromatographic separations were analyzed using XCMS, then processed for cleanup/assignment to LIPID MAPS categories, using LipidFinder (Figure 1A and 1B).^14^ Plasma quality was checked through careful comparison with fresh plasma, detailed in Materials in the Data Supplement. Most lipid categories were unchanged, however, oxidized phospholipids and lysoPCs had elevated somewhat in storage (Figures I through III in the Data Supplement). This is not unexpected, and we include a full discussion of this phenomenon in Materials in the Data Supplement. To assess the impact of the rs10757274, A>G SNP, we compared AA (n=39) with the risk genotype GG (n=33). Data were analyzed first using the Mann-Whitney *U* test, then chromatograms for all features with *P*<0.075 were manually checked for quality. LipidFinder detected 1878 lipids, with 872 assigned to a category (Figure 1B). Next, quantile normalization was applied followed by the Mann-Whitney *U* test, and then a *P* value adjustment using sequential goodness of fit metatest to each subclass.^15^ The sequential goodness of fit metatest has been shown as especially well-suited to small sample sizes when the number of tests is large. This data is shown in volcano plots in Figure 1C through 1J, and the *P* values are in column M (Tabs I and II in the Data Supplement). Those most affected by genotype were glycerophospholipids and unknowns (Figure 1C through 1J, Table 1). Following *P* value adjustment the number of significantly different lipids was 17, with 7 putatively identified as lysoPC ions and adducts (Tabs I and II in the Data Supplement). An additional group of 8 had *P* values close to significance at 0.05 to 0.08. All were reduced in GG plasma. As this method is used as for hypothesis generation only, we next validated our results using gold-standard quantitative targeted methods.

**Table 1.**
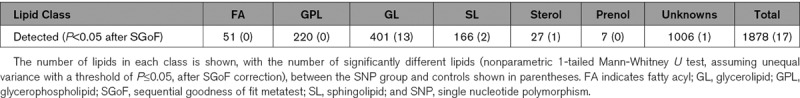
Number of Detected and Identified Lipid Features in the Global Lipidomics Assay

### Quantitative Targeted Lipidomics Confirms Decreased LysoPLs in the GG Samples

**Figure 2.**
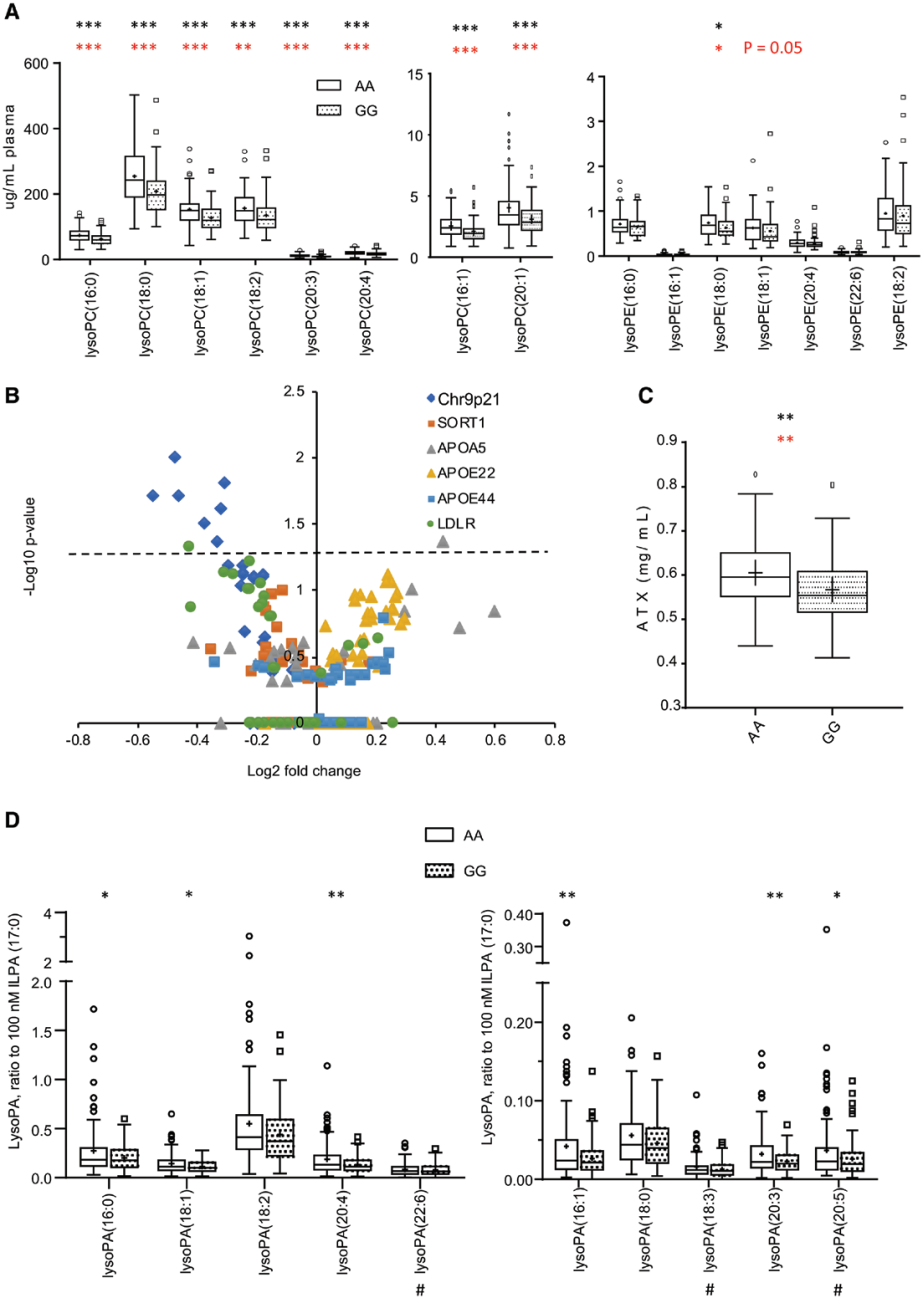
**LysoPLs (Lysophosphospholipids) are significantly reduced in rs10757274 GG but not in subjects with unrelated SNPs.** **A**, Several LPCs are lower in GG samples than AA controls, and LPEs trend towards lower levels. LysoPLs were determined using LC/MS/MS as described in Methods (n=88 AA and 81 GG). Tukey box plot, *P<0.05, **P<0.01, ***P<0.005, 2-tailed, unpaired Student *t* test (black) and Mann-Whitney *U* (red). **B**, Plasma lysoPL are not altered by other risk SNPs. Plasma from the NPHSII (Northwick Park Heart Study II) cohort containing several risk (up or down) SNPs were analyzed using LipidFinder, and m/z values corresponding to lysoPL extracted and compared. These are plotted on a volcano plot, to show fold change vs significance, following *P* value adjustment using sequential goodness of fit metatest (SGoF). Numbers and genotypes are shown in Table I in the Data Supplement. **C**, ATX (Autotaxin) is significantly decreased in GG samples compared with AA controls. Plasma ATX activity was measured as described in Methods (n=47 AA and 49 GG). **D**, LysoPAs (Lysophosphatidic acids) are significantly decreased in GG plasma compared with AA controls. Plasma lysoPAs were measured as described in Methods, using LC/MS/MS (n=95 AA and 100 GG). Tukey box plot, *P<0.05, **P<0.01, ***P<0.005, 2-tailed, unpaired Student *t* test (black). LC/MS/MS indicates liquid chromatography-tandem mass spectrometry; and SNP, single nucleotide polymorphism.

The same plasmas were analyzed using a targeted fully quantitative assay for 15 lysoPLs (lysophosphospholipids). Of these several lysoPCs significantly decreased, with both lysoPC and lysoPEs all trending towards lower levels in GG (Figure IVA in the Data Supplement). This was replicated using new samples from NPHSII (n=47: AA, 49: GG; Figure IV in the Data Supplement). When both data sets were combined (n=82−86/group), all 8 lysoPCs were significantly lower in the GG genotype (Figure 2A). Thus, lysoPLs are overall suppressed in the GG genotype, with a more robust effect on lysoPCs than lysoPEs.

### Significantly Altered LysoPLs Are Not Detected in 5 Additional CHD Risk-Altering SNPs

LipidFinder data were analyzed for additional SNPs from the NPHSII cohort, comparing subjects homozygous for the common alleles with subjects homozygous for rare protective alleles for *SORT1*, *LDLR* or *APOE* E2/E2, or rare risk alleles *APOA5* or *APOE4/E4* (Table I in the Data Supplement). For most lysoPLs, levels were not significantly altered, with the exception of one for *APOA5* (upregulated, lysoPE(18:1), and one for *LDLR* [downregulated, lysoPC(18:2)]; Figure 2B). This indicates that lysoPLs are consistently reduced only in the GG risk SNP rs10757274.

### The Plasma LysoPA/ATX Axis Is Dysregulated in the GG Group

Next, lysoPL-related metabolites/enzymes were measured. Metabolism of lysoPL to lysoPA (lysophosphatidic acid) in healthy plasma can be mediated by ATX (autotaxin).^16^ Here, we used a targeted liquid chromatography-tandem mass spectrometry assay for lysoPAs and an immunoenzymatic assay for ATX. ATX was significantly decreased (*P*=0.026). Based on power calculations (Material in the Data Supplement), an additional set of plasmas was included to increase sample numbers to 95 to 100 per group for lysoPAs. Liquid chromatography-tandem mass spectrometry demonstrated overall small reductions but with several being significantly lower (Figure 2C and 2D). Taken with the lysoPL data, this indicates a global suppression of lysoPL/lysoPA/ATX metabolic pathway in the GG group.

**Figure 3.**
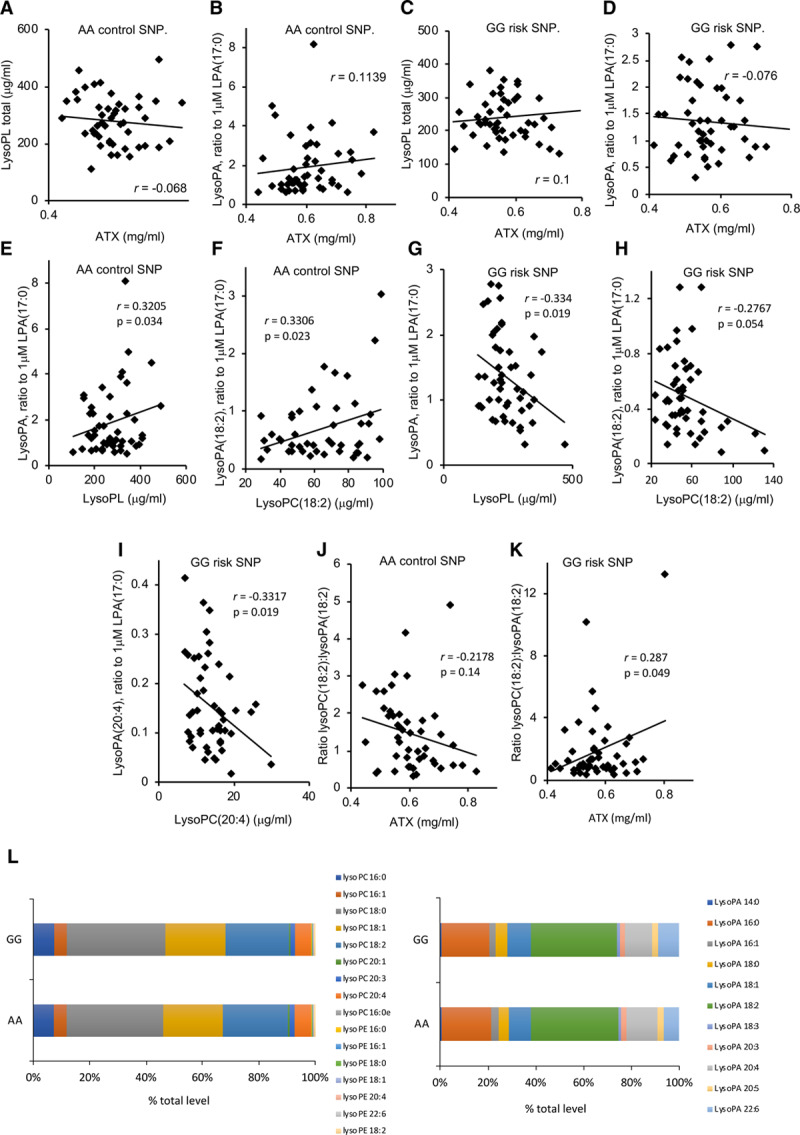
**The lysoPL (lysophosphospholipids)/lysoPA (lysophosphatidic acid)/ATX (autotaxin) axis is dysregulated in the GG plasmas, while the profile of molecular species is unchanged for lysoPL/lysoPA.** **A–D**, ATX shows altered correlations with plasma lysoPL or lysoPA in GG vs AA plasma. Levels of lysoPL or lysoPA quantified by LC/MS/MS in the validation cohort were correlated using Answerminer, to determine Pearson correlation coefficient. **A** and **B**, AA control plasma, (**C** and **D**) GG risk plasma (n=47 AA and 49 GG). **E–I**, LysoPL and lysoPA are positively correlated for AA plasma, but negatively correlated for GG. The sum of all lysoPAs or lysoPLs in each set were correlated using Answerminer, as above (**E** and **G**). Alternatively, lipids containing 18:2 or 20:4 were separately correlated (**F**, **H**, and **I**). **E** and **F**, AA control plasma, (**G**, **H**, and **I**) GG risk plasma (n=47 AA, 49 GG). **J** and **K**, The lysoPA(18:2)/lysoPL(18:2) ratio positively correlates with ATX in AA plasma, but negatively for GG, indicating a block in substrate:product conversion in GG. Correlations were performed using Answerminer (n=47 AA and 49 GG). **J**, AA plasma, (**K**) GG plasma. *P*<0.05 indicates significant using the Pearson correlation test. **L**, The profile of individual lysoPL or lysoPA molecular species is unchanged between GG and AA plasmas. Levels of individual lysoPL/lysoPA were compared across both groups and shown as %. LC/MS/MS indicates liquid chromatography-tandem mass spectrometry.

Next, correlation analysis was undertaken to examine the contribution of ATX in metabolizing lysoPL to lysoPA. In AA plasmas, ATX showed very weak positive or negative correlations with total lysoPL or lysoPA, respectively (Figure 3A and 3B). This agrees with reports that ATX contributes to lysoPL conversion to lysoPA in healthy subjects.^16^ In contrast, in GG plasmas, these weak trends were somewhat reversed (Figure 3C and 3D). To look in more depth, we correlated substrates with products (Figure 3E through 3H). In the AA group, significant positive correlations were seen for total lysoPA with lysoPL (*P*=0.034). Comparing lipids with the same fatty acyl, significant correlation was seen between lysoPA(18:2) and lysoPL(18:2) (*P*=0.023; Figure 3E and 3F). This indicates that as the pool of lysoPL increases, the level of lysoPA increases in parallel, and this would be consistent with conversion by ATX. This relationship was fully reversed in the GG group, where total lysoPL, lysoPL(18:2), or lysoPL(20:4) were negatively correlated with their corresponding lysoPAs (*P*=0.019, 0.054, and 0.019, respectively; Figure 3G through 3I). We next analyzed correlation slopes for AA versus GG, comparing either lysoPL:lysoPA (Figure 3E versus Figure 3G), or lysoPL(18:2):lysoPA(18:2) (Figure 3F versus Figure 3H). Both these comparisons revealed significant differences (*P*=0.0264 and 0.0029, respectively).^17^ These data confirm altered metabolism of lysoPL and lysoPA lipids between genotypes. Specifically, conversion of lysoPL to lysoPA appears to be suppressed in the GG homozygotes.

The direct contribution of ATX to metabolizing lysoPL to lysoPA was next examined by correlating normalized ratios of lysoPC(18:2):lysoPA(18:2) with ATX. In this comparison, we expect that as ATX increases, the ratio of substrate:product would reduce due to their interconversion. For AA plasma, a weak negative correlation was seen (Figure 3J). In contrast, a significant positive correlation was observed for GG plasma (Figure 3K). Thus, as ATX increases, a higher ratio of substrate:product was seen in GG, suggesting a decoupling of ATX from metabolizing lysoPL to lysoPA. Comparing the slopes for AA versus GG revealed significant differences based on genotype (*P*=0.0157). This further underscores the dysregulation of the lysoPL metabolic pathway in the GG group and suggests that non-ATX pathways may mediate lysoPL to lysoPA conversion. Last, the relative ratios of all lysoPL and lysoPA molecular species were unchanged in the GG versus AA groups (Figure 3L). Thus, while metabolism of lysoPL/lysoPA by ATX is altered, there was no influence of genotype on molecular composition overall. Notably, ATX preferentially metabolizes unsaturated lysoPCs.^18^ Overall, despite the correlations only showing associations, when taken with our observations that plasma from GG subjects has significantly less ATX protein and that all lysoPC molecular species are similarly affected, our data strongly evidence that there is less involvement of ATX in metabolizing these lipids in GG plasmas.

**Figure 4.**
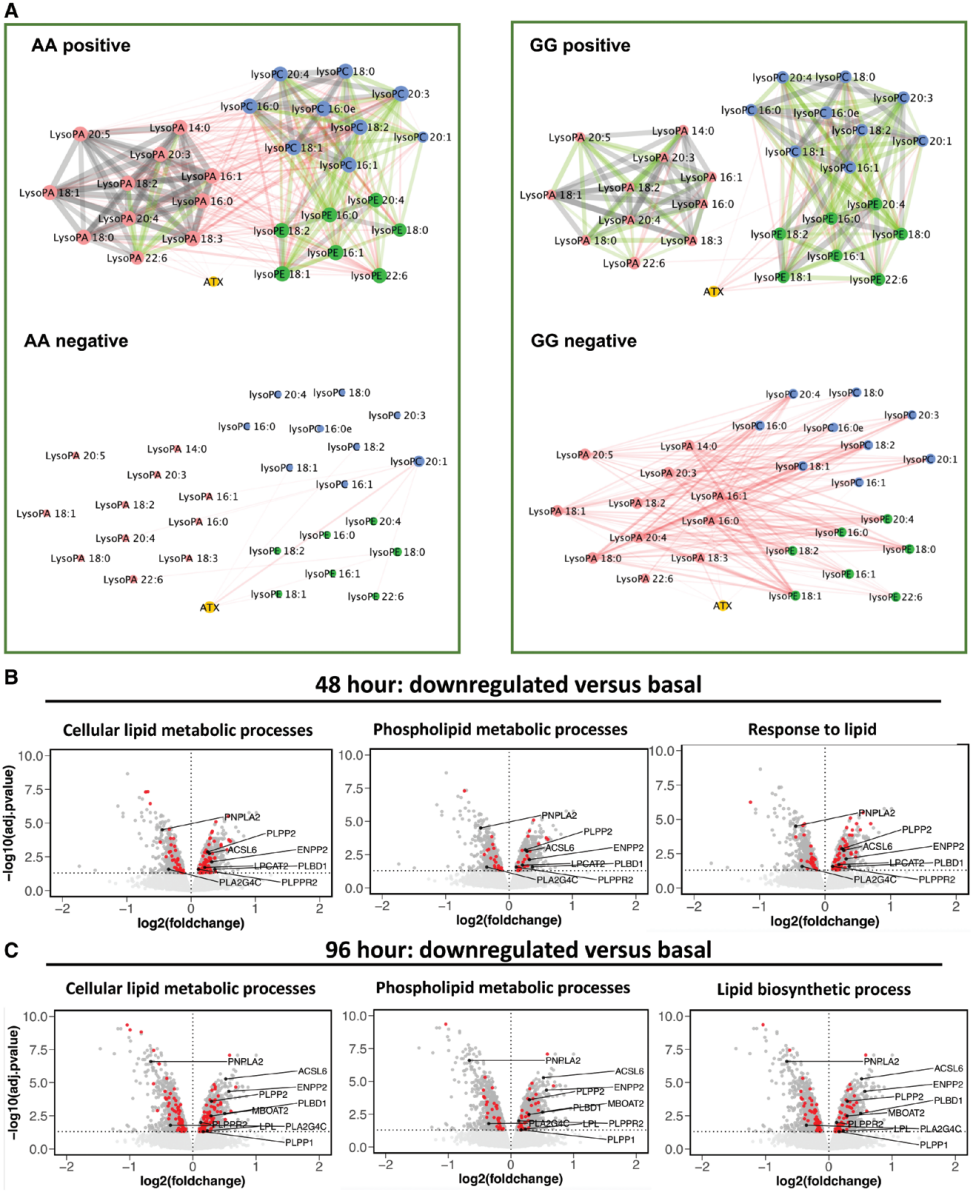
**Cytoscape analysis of lipids reveals divergent metabolism in GG vs AA, while ANRIL knockdown is associated with significant changes to lysoPL (lysophosphospholipids)/lysoPA (lysophosphatidic acid)-metabolizing genes.** **A**, Cytoscape reveals strong links within related families, but a positive-negative switch for lysoPL-lysoPA correlations between AA-GG plasmas. Pearson correlation networks were generated for the AA and GG validation samples (n=47 AA and 49 GG), using lipid concentrations. Nodes are colored by lipid sub-category and represent individual molecular species, and edges represent the correlation. Edges detail the Pearson correlation coefficients between nodes (lipids), where the width of the edge denotes value. Additionally, edges are colored by value: red (*r*=0.10–0.39); green (*r*=0.40–0.69); gray (*r*=0.70–1.00). **C** and **D**, Significant changes in lipid regulatory gene expression are observed with ANRIL knockdown in cell culture. Affymetrix array data generated in Congrains et al^5^ was analyzed using GO as described in Methods. Volcano plot showing differential gene expression of all genes on the Affymetrix HuGene1.0 v1, chip. LysoPL/lysoPA regulating genes that alter in line with decreased levels of the lipids in GG plasma are labeled. The horizontal dashed line shows where adj. *P* value <0.05 (Benjamini-Hochberg correction) where points (genes) above this line are significantly differentially expressed. LysoPL-regulating genes that alter in line with decreased levels of the lipids are labeled in black. Genes in red are annotated to the GO term detailed in the plot title. Data are plotted in R using ggplot2. **B**, Forty-eight hour shRNA knockdown. **C**, Ninety-six hour shRNA knockdown.

Next, a Pearson correlation analysis looking at relationships between individual lipids and ATX was next undertaken using Cytoscape. For thresholds, the classification system of Schober was used.^19^ Here, we see that there are moderate (*r*=0.40–0.69, green) or strong (*r*=0.70–1.00 gray) correlations between lipids of the same class, while there are weak (*r*=0.10–0.39, red) correlations between different lipid classes (Figure 4A). Importantly, the key difference in the data set is that the weak correlations between classes are positive for the AA group, while they are negative for the GG group (Figure 4A). Overall, this indicates that these lipids behave similarly within AA subjects. In contrast, in GG plasma, while lipid classes still positively correlate within their groups (eg, lysoPCs correlate strongly with each other), the links between lysoPL and lysoPA are lost. Instead correlations were weakly negative between lysoPE and lysoPA (Figure 4A). As in Figure 4, ATX weakly positively correlates with lysoPA in the AA group but instead with lysoPL in the GG group. This analysis reinforces our findings of altered metabolism for lysoPL/lysoPA but here at the level of individual lipid species.

### ANRIL Knockdown Significantly Alters Lipid and LysoPL Metabolism Gene Expression

Chr9p21 risk SNPs are believed to act via altering expression of ANRIL, which regulates cell proliferation/senescence in vitro.^2,4,5^ To examine for a functional link with lysoPL/lysoPA metabolism, we analyzed the effect of shRNA downregulation of the proximal ANRIL transcripts EU741058 and DQ485454 in HEK-293 cells at 48 and 96 hours.^12^ A GO analysis found significant alterations of several lipid pathways by ANRIL, including Regulation of Lipid Metabolic Processes (GO: 0019216), Phospholipid Metabolic Processes (GO:0006644), Cellular Lipid Metabolic Process (GO:0044255), and Lipid Biosynthetic Processes (GO:0008610), for example, Regulation of Lipid Metabolic Processes was 1.9- or 1.88-fold enriched (false discovery rate <0.05, Benjamini-Hochberg), respectively, at 48 and 96 hours, respectively (Table 2, Tabs III and IV in the Data Supplement). Thus, large numbers of lipid-associated genes were significantly differentially regulated (Tabs V and VI in the Data Supplement).

**Table 2.**
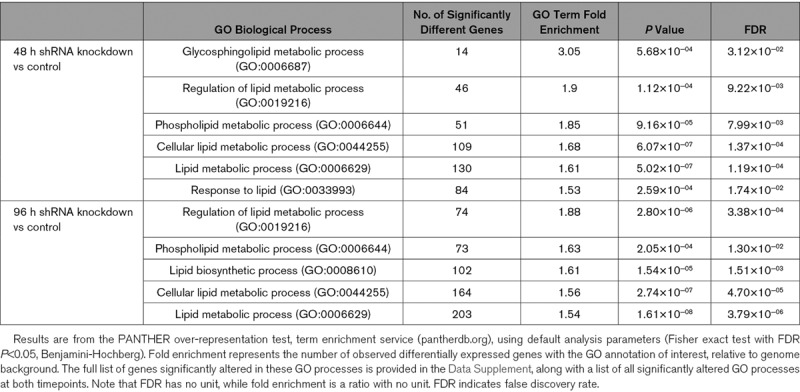
Several Lipid Related Gene Ontology Pathways Are Significantly Regulated by ANRIL Silencing in HEK-293 Cells

We next examined the effect of ANRIL knockdown on 49 candidate lysoPL metabolism genes (Tab VII in the Data Supplement). Of these, 9 were significantly changed at both time points and another 6 at a single timepoint (Table 3). Several were consistent with lowered lysoPL/lysoPA, including reduced *PNPLA2, PLA2G4C*, increased *LPCAT2, MBOAT2, ACSL6, PLBD1, PLPP1, PLPP2*, and *PLPPR2* (Table 3, Figure 4B and 4C). Additional relevant genes were regulated, but in the opposing direction, including decreased *LPCAT1* and *LPCAT3* and increased *LPL, PLA2G7*, and *DGKA* (Table 3). *ENPP2* (the gene encoding ATX) was significantly increased by ANRIL suppression (Table 3, Figure 4B). This data is displayed in volcano plots of the full Affymetrix data set (Figure 4B and 4C and Figure V in the Data Supplement). Genes in red represent significantly different lysoPL metabolizing genes from the lipid GO pathways.

**Table 3.**
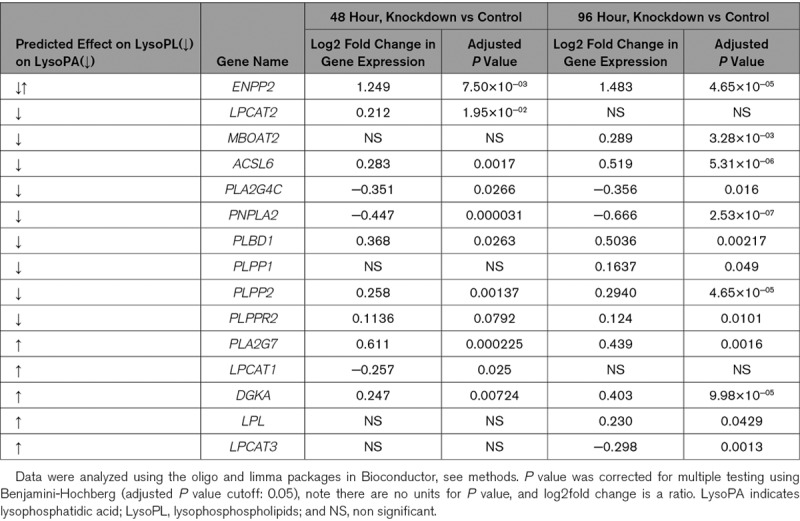
Several LysoPL Relevant Genes Are Significantly Altered in ANRIL Knockdown

### Vascular Smooth Muscle Cells Generated From Induced Pluripotent Stem Cells From Chr9p21 Risk Haplotypes Show Altered Expression of LysoPL Metabolism Genes and Correlation of Expression With ANRIL Isoform Expression

**Figure 5.**
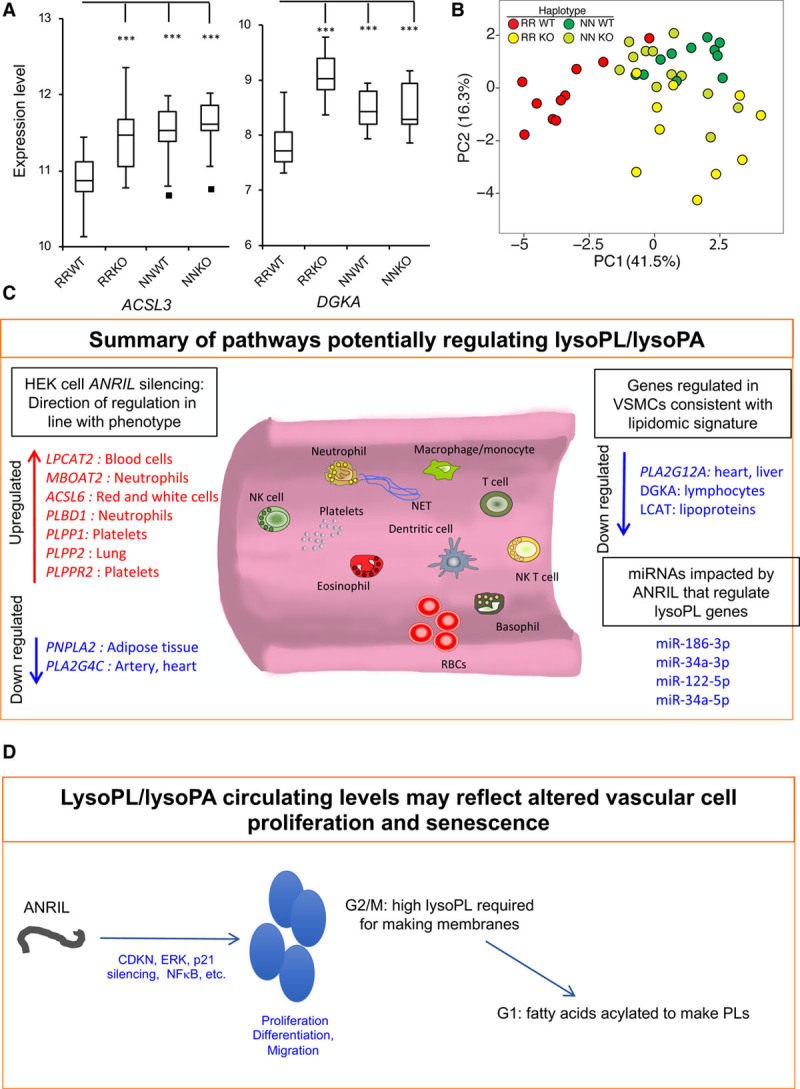
**VSMCs from risk haplotypes show differential gene expression of lysoPL (lysophosphospholipid) metabolizing genes that are rescued by deletion of the Chr9p21 locus.** **A**, PCA shows that the presence of risk haplotypes is associated with differential gene expression of lysoPL genes. Induced pluripotent stem cells from peripheral monocytes were obtained and differentiated as described in Materials in the Data Supplement. RNAseq data were clustered using lysoPL metabolizing genes by PCA in R. Nonrisk haplotype (NNWT), risk haplotype (RRWT) and their genome edited counterparts (NNKO and RRKO) are shown. **B**, Example data sets for ACSL3 and DGKA, showing that removing the risk locus reverts gene expression back to levels in nonrisk individuals. *P<0.05, **P<0.01, ***P<0.005, Students *t* test, n=9–10 clones per group. **C** and **D**, Schematics showing impact of ANRIL silencing or risk haplotypes on relevant lysoPL metabolizing genes. PCA indicates principal component analysis; PC1, principal component-1; and VSMCs, vascular smooth muscle cells.

Vascular smooth muscle cells (VSMCs) generated by differentiation of induced pluripotent stem cells from humans homozygous for risk haplotypes in Chr9p21 show globally altered transcriptional networks, dysregulated adhesion, contraction, and proliferation, with deletion of the risk haplotype rescuing the phenotype.^13^ Here, we interrogated an RNAseq data set of mature iPSC-derived VSMCs for expression of the 49 lysoPL metabolism genes (Tab VIII in the Data Supplement). Examination of individual genes revealed 14 that were significantly different between risk haplotypes (RRWT) and other lines, and where removal of the risk locus in RR led to partial or complete rescue: *ACSL3, DGKA, PLA2G2A, LPCAT2, LPL, PLA2G3, PLPPR2/LPPR2, PLA2G12A, PLPP1/PPAP2A, LCAT, PLA2G6, ACSL1, MBOAT2*, and *PNPLA3* (Figure 5A, Figure VI in the Data Supplement). Of these, *DGKA, PLA2G12A*, *and LCAT* were regulated in line with reduced lysoPC/lysoPA.

Multivariate analysis using principal component analysis for expression of these 14 genes shows clear separation of VSMC lines containing the RRWT from controls (NNWT) in PC-1 (Figure 5B). When the risk locus was deleted, the resulting RRKO cell lines instead clustered closer to NNWT and NNKO in PC-1 (Figure 5B). This analysis indicates that expression of several lysoPL metabolizing genes is different in risk haplotype cells but reverts closer to NN on removal of the 9p21 locus.

Next, correlations of genes that metabolize lysoPLs with ANRIL isoforms (exons 6–7 and 18–19) were performed. Deletion of the Chr9p21 locus in the KO VSMC lines starts around exon 9 and runs downstream to the end of the coronary artery disease region.^13^ Analysis of ANRIL was performed by qPCR detection of ANRIL isoforms containing exons 6 and 7 (present in long and short isoforms) and exons 18 to 19 (in long isoforms only). The analysis showed a significant increase of isoforms containing exons 6 and 7 in RRWT cells, compared with NNWT cells (Figure VII in the Data Supplement), as previously described.^13^ ANRIL expression was minimal in NNWT, with levels comparable to a residual expression of ANRIL detected in both KO lines, possibly due to transcription of truncated transcripts. ANRIL analysis performed using detection of exons 18 and 19 showed no significant differences between RRWT and NNWT cells. No transcript expression was detected in KO lines as expected because the deletion encompasses the last 10 exons of the ANRIL gene. These analyses confirmed that ANRIL short isoforms containing exons 6 and 7 but not 18 and 19 are upregulated in RRWT VSMCs. To evaluate possible correlations between lysoPLs-related genes and ANRIL expression, all samples were used for ANRIL (exons 6–7) analysis, while for correlations with ANRIL (exons 18–19), only WT (RR and NN) were tested. Circular ANRIL isoforms have not been detected in these cells.

#### ANRIL (Exons 6–7)

Several genes correlated significantly, either in a positive or a negative direction with these ANRIL isoforms (Figure VIII in the Data Supplement). RRWT samples (in red) clustered together as groups, separated from all other samples, which were seen to express phospholipid (PL) metabolism genes similarly. This was somewhat expected because these PL metabolism genes were differently expressed in RRWT versus RRKO, NNWT, NNKO, as shown for ANRIL (6–7) expression (Figure 5B, Figure VII in the Data Supplement). However, the significant Pearson correlations between ANRIL (exons 6–7), and the individual genes show a direct association between this form of these ANRIL isoforms and some lysoPL genes.

#### ANRIL (Exons 18–19)

Here, correlations were tested using RRWT or NNWT clones separately and then compared. Five genes were identified where a significant negative correlation between ANRIL (18–19) and lysoPL gene expression was seen (*PNPLA3*, *DGKA*, *ENPP2*, *LPCAT3*, and *PLA2G4C*) in the RR clones. In contrast, correlations in NN samples were weaker and not significant (Figure IX in the Data Supplement). This suggests an impact of ANRIL (18–19) isoforms on gene expression, that is absent/reduced in NN. One NN clone displayed higher levels of ANRIL (18–19) compared with others, and as an outlier had a large impact on the correlation, reducing statistical power.

### In Silico Analysis of miRNA Databases Suggests Potential Candidates for ANRIL Regulation of LysoPL Gene Expression

ANRIL displays sponge activity towards miRNAs.^20^ To examine whether this could mechanistically link ANRIL with lysoPL gene expression, we undertook an in-silico analysis using 2 databases (including one that is experimentally validated: TarBase v8 (http://carolina.imis.athena-innovation.gr/diana_tools/web/index.php?r=tarbasev8%2Findex) and TargetScan (*http://www.targetscan.org/vert_72/). We searched whether miRNAs known to be inhibited by ANRIL interact with PL-metabolizing genes that are altered in HEK or VSMC data sets. Here, the expected outcome is that target genes should be regulated in the same direction as ANRIL. Some hits were found, including 2 that were conserved across both data sets. In the HEK data, the miRNAs that interact with downregulated genes were miR-186-3p, miR-34a-3p (*LPCAT1*) and miR-122-5p, miR-34a-5p (*LPCAT3*). In the VSMC data set, where ANRIL (exons 6–7) is significantly upregulated in RR, we focused on genes that were elevated in RR and reduced when the locus was deleted. Here, we found miR-34a-5p (*PLA2G6*) and miR-122-5p (*PNPLA3*). These hits were all from the experimentally validated database (Tarbase) and differences in the target genes impacted may be due to the different cell types used.

## Discussion

Lipidomics MS is increasingly applied to prospective CHD cohorts that contain no genetic information, while conversely GWAS studies have examined associations with traditional lipid measures only (eg, total cholesterol or triglycerides).^21–35^ Cohorts are only now starting to examine the association of individual lipid molecular species with specific risk SNPs, and little information on this is yet available. Cohort lipidomics is an area that is increasing in popularity, however, there are some serious pitfalls with using only untargeted methods. Including a high degree of validation, we show that a common Chr9p21 (rs10757274, A>G) CHD-risk SNP is associated with metabolic alterations to the lysoPL/lysoPA/ATX axis in human plasma (Figures 1 through 4). This revealed a genotype-specific change that was absent in 5 other GWAS-proven CHD-risk SNPs. Since the action of rs10757274 GG is independent from traditional lipid measurements, it may represent a different component of the disease, characterized, in part, by changes to bioactive signaling PLs, rather than storage/energy lipid pools.^1^

LysoPLs have an emerging role in cardiovascular disease that is not yet understood. In vitro, they mediate GPCR (G protein-coupled receptor) signaling that causes immune cell migration and apoptosis. This has led them to be proposed as proinflammatory.^36–40^ However, this is disputed since most lysoPL is bound to albumin, immunoglobulins, and other plasma carrier systems, and levels are already higher than required for mediating GPCR activation.^41–43^ Importantly, recent cohort studies have shown that plasma lysoPC is inversely related to incidence of an event. These include Malmö, Bruneck, TwinGene, ULSAM, and PIVUS, which showed correlations of lower lysoPC with incident CHD risk, using untargeted lipidomics.^31,44,45^ Also, patients on hemodialysis show higher risk of a CHD event and elevated mortality with lower lysoPC.^43^ Lower lysoPCs are also associated with CHD factors such as visceral obesity, although since lysoPA cannot be measured using shotgun or untargeted methods, we have not found other cohort data that includes this lipid as yet.^27,31,32^ In the Bruneck cohort, inclusion of lysoPCs in classifiers improved power for CHD-risk prediction, indicating that although the reduction is rather modest, it is clinically significant.^31^ Furthermore, the Malmö cohort reported that CHD development is preceded by reduced levels of lysoPCs, around 8% similar to our data.^44^ In Malmö and Bruneck, the lipidomics was limited to untargeted and shotgun methods without further validation, thus our new data provides stronger analytical confidence while linking their findings to a specific risk locus. Given the prevalence of rs10757274 GG in the general population (≈23%), our data may at least, in part, explain the findings in other cohorts with lower lysoPLs now associating with a subgroup with a common SNP. In contrast, it is also known that elevations in long-chain unsaturated lysoPA maybe a feature of an acute cardiac event, where a sudden plaque rupture results in generation/release, likely via activation of platelet phospholipases.^18^

In addition to lipid class-specific changes in PLs, many significantly decreased unknowns were found, which are currently absent in databases (Figure 1). The plasma lipidome contains large numbers of such species, and a significant challenge lies in their structural and biological characterization. The comprehensive list of all lipids detected with fold change and significance levels is provided (Tab I in the Data Supplement) as a resource for further mining.

We next searched for potential mechanisms to explain the lipid changes using data sets from 2 cell models of ANRIL modulation because there is increasing evidence that this long noncoding RNA plays a central role in Chr9p21-linked cardiovascular disease (CVD). ANRIL is expressed by exons contained within Chr9p21, and there are many isoforms, including long, short, and circular, resulting from alternative splicing across several exons. Both increases and decreases of various ANRIL transcripts have been reported to be associated with CVD. For example, compared with AA individuals, GG, and several other Chr9p12 risk SNPs have almost 50% lower ANRIL (exon 2) in peripheral blood cells.^2^ This agrees with the finding that multiple risk alleles are associated with a decrease in ANRIL (exons 1–2) in peripheral blood mononuclear cells.^5^ However, others showed various ANRIL transcripts are increased in carriers of risk alleles, including from exons 1 to 5, 7 to 13, and 18 to 19, with no change at exons 7b or 10 to 13b.^3^ Elsewhere, expression of short variants (exons 1–2, ending with alternative 13, and exons 1, 5–7+13) were increased while long variants (coded by exons 1–12+14–20) were decreased in risk allele carriers.^6^ In the VSMC data set used in our study, lines carrying risk haplotypes (which were also GG for rs10757274) expressed higher levels of ANRIL transcripts (exon 6–7) compared with NNWT, which have a minimal expression, similar to a residual expression detected in KO lines.^13^ In contrast, the HEK inducible knockdown targeted proximal alternatively spliced ANRIL transcripts EU741058 (exons 1, 5–7, and 13) and DQ485454 (exons 1–12 and alternative exon 13). Thus, while they both model human CVD they differ significantly in terms of their impact on ANRIL.

While it is known that *ANRIL* gene products regulate metabolic genes in cultured cells and stimulate VSMC proliferation while reducing adhesion and contraction, the impact of ANRIL on lysoPL/lysoPA metabolism is not characterized.^12,13^ Here, we showed in both HEK and VSMC data sets that there was a significant impact on a large number of lysoPL/lysoPA metabolism genes, with GO term analysis identifying a large number of lipid terms being significantly altered in HEK cells. In the case of VSMCs, removal of the risk locus indicated that several affected genes were directly influenced by the Chr9p21 locus (Figure 5A and 5B). Our in-silico screen also identified 4 miRNAs across the cell types with 2 candidates identified from both HEK and VSMC lysoPL gene regulation: miR-34a-5p (*LPCAT3* and *PLA2G6*), miR-122-5p (*LPCAT3* and *PNPLA3*). Collectively, this suggests that Chr9p21 risk alleles may alter lysoPL/lysoPA in humans via ANRIL regulation, providing novel insights into the biology of this important cause of CHD.

Many of the candidate genes are expressed in leukocytes, platelets, erythrocytes, heart, adipose tissue, and plasma, thus measuring them in plasma is not possible. However, we could measure ATX (*ENPP2*), a plasma enzyme that converts primarily unsaturated lysoPC to lysoPA in healthy subjects.^18^ ATX protein was reduced, and furthermore, correlation analysis was consistent with lower ATX activity (Figures 2C and 3A through 3K), providing a potential explanation for lower lysoPAs in the GG group. In line with this, *ENPP2* expression negatively correlated with ANRIL (exons 18–19) in VSMC from the risk group, suggesting an association between this gene and a risk form of ANRIL (Figure IX in the Data Supplement). Also, *ENPP2* was elevated in HEK cells, which lack ANRIL transcripts that contain exons 6 and 7. Furthermore, it has been reported that inflammatory cytokine induction of *ENPP2* is suppressed by 50% in primary human monocyte-derived macrophages that carry the Chr9p21 risk haplotype allele.^46^ The contribution of ATX to CHD is not understood and may vary with underlying genetic cause.^47^ Indeed, while it metabolizes lysoPL to lysoPA in health, in acute coronary syndromes other pathways appear to predominate.^18^ This mirrors our suggestion that ATX might be less involved in plasmas with elevated CVD risk, with other uncharacterized pathways being relevant. Here, significant downregulation of ATX in GG plasma from middle-aged men who are otherwise healthy and without clinically detectable CHD was seen indicating it precedes cardiovascular events in this group (Figure 2C). While we suggest that reduced lysoPA maybe at least, in part, relate to reduced levels of plasma ATX, additional candidates were identified through transcriptional analysis, including *PLPP1*, *PLPP2, PLPPR2* (all induced in HEK cells), or *DGKA* (reduced in RRWT VSMC cell lines).

**Figure 6.**
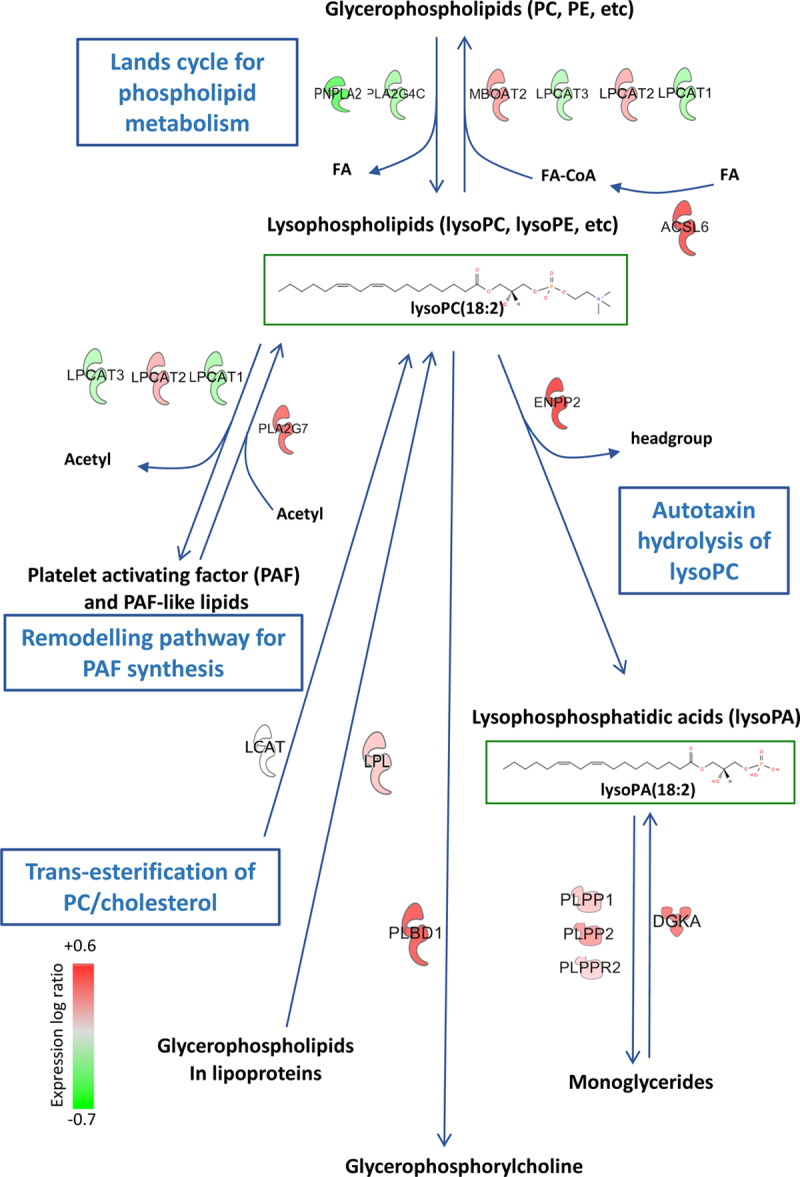
**Metabolic pathway showing lysoPL (lysophosphospholipids)/lysoPA (lysophosphatidic acid) regulatory genes that are significantly altered by ANRIL knockdown in HEK cells.** Genes that metabolize these lipids are shown. Full data on their transcriptional regulation is provided in Table 3, Tab VII in the Data Supplement. LCAT was not significantly regulated but is shown for completeness.

In healthy subjects, lysoPLs, particularly lysoPC, circulate at relatively high concentrations, where they could be generated by (1) lipases bound to the cell surface of endothelial cells in liver, heart, and adipose tissues (*LPL*, *LIPC*, and *LIPG*), (2) Land’s cycle enzymes in circulating blood cells/platelets,^16^ (3) lecithin-cholesterol acyl transferase (*LCAT*) trans-esterification in the liver, or (4) by remodeling pathways for PAF (platelet-activating factor) removal (Figure 6). In healthy tissue, lipases predominate but during vascular inflammation the balance may alter, but this is not well characterized. The Land’s cycle involves phospholipase A2 hydrolysis, although the isoforms controlling blood levels are not fully known. Candidates include stromal isoforms and cellular or secreted phospholipase A2 from circulating cells and platelets that may become relevant during inflammation. Also, a role for circulating/platelet phospholipase A1 from platelets in lysoPL formation has been proposed.^40^ The reduction in lysoPCs is consistent with significantly different genes identified in the data sets, for example, *ACSL6, MBOAT2, LPCAT2, PLBD1* (all induced in HEK cells), *PNPLA2* and *PLA2G4C* (downregulated in HEK cells), and *PLA2G12A* and *LCAT* (reduced in RRWT VSMC cell lines). Notably, the transcriptional data sets showed complex and largely different changes in lysoPL gene expression, which may relate to them being from different cell types, and with different manipulations to ANRIL isoform expression. However, 4 genes were significantly altered across both data sets: *MBOAT2 and LPL* (always upregulated) and *DGKA, PLPP1* (increased in HEK, decreased in VSMC). A detailed discussion of the known expression patterns and roles of all these genes is provided in Material in the Data Supplement.

Notably, many of the genes found to be altered in the transcriptional data are genes with known SNPs that correlate with traditional lipid measures. For many of these genes, it is not clear how or even if they regulate lipid levels directly or are simply associated with altered levels (in the case of traditional lipids). Cardiovascular disease is complex with different forms, however, in the case of Chr9p21-linked CHD, a strong phenotype of coronary artery disease is noted.^48^ Similarly, traditional lipid levels are also strongly associated with a strong phenotype of coronary artery disease. This indicates that clinical outcomes are similar despite the different genetic origins and for this to be the case, some convergence in biochemical or cellular pathways downstream of genetics would be expected. A potential explanation for our findings is that downstream of ANRIL, some of the same genes that are already known to be genetically associated with CHD are subtly altered at their gene expression levels through transcriptional mechanisms, such as proposed here (eg, miRNA sponge activities of ANRIL), and that this then goes on to impact inflammation. This would promote the development of coronary artery disease, through some common mechanisms, independent of the biology of traditional lipoprotein measures. While several genes were found to be altered in either the HEK or VCMC data sets, more research is required to link gene expression changes with lysoPC/lysoPA levels, including protein and enzyme activity measurements from carriers of Chr9p21 risk SNPs. LysoPCs and lysoPAs are metabolized in a complex manner, with many gene products in the vasculature expected to play a contributing role. It is also the case that standard lipids are metabolized in a complex manner and that changes in one relevant gene could be compensated by changes in others, that overall lead to standard lipids such as triglycerides and cholesteryl esters being overall unchanged in these subjects.

We also note that many genes in the HEK or VSMC analysis changed in directions that on their own could be considered to predict increases in lysoPC/lysoPA. However, overall it is the combination/balance of several enzymatic activities that will determine the flux of lipids through this pathway, and thus, their overall levels at steady state. Thus, changes in some could be over-compensated by changes in others, leading to the observed phenotype of overall reduced levels of these lipids. For further reference, genes with SNPs that have been found to associate with standard lipid traits and were also found to change herein are listed in.^49,50^

A final question relates to how ANRIL and lysoPLs are functionally connected. PL metabolism is finely tuned during cell proliferation, with higher concentrations of lysoPC and lysoPE detected at G2/M, which fall dramatically along with concomitant increases in PC/PE due to acylation during progression to G1.^51^ This provides the PL membranes required to complete the cell cycle. Given ANRIL’s ability to regulate cell proliferation and observations that silencing ANRIL prevents division and promotes senescence, the lower levels of lysoPLs in plasma may simply reflect altered rates of cell turnover in the vasculature, but this remains to be determined (Figure 5C and 5D). Here, 4 miRNAs known to be regulated by ANRIL that also suppress expression of selected lysoPL genes across both HEK and VSMC data sets were found miR-186-3p, miR-34a-3p, miR-122-5p, and miR-34a-5p. These potential hits can be followed up as candidate downstream mediators of ANRIL’s regulation of lysoPL/lysoPA metabolism. Several have well-known roles in regulating proliferation and notably circulating miR-122-5p associates with acute myocardial infarction.^52^

In summary, we reveal an association of altered PL metabolism with CHD risk in a common risk SNP. The alterations in multiple lysoPL/lysoPA regulatory pathways seen on ANRIL silencing, or the presence/removal of the risk locus in vitro, further suggest the involvement of bioactive signaling lipids in this form of vascular disease, and mechanistic studies are warranted. To this end, fresh blood from AA and GG subjects is required to measure plasma and cellular levels of all candidate enzymes and relevant lipids, to identify how lysoPC/lysoPA metabolism is altered by the presence of the risk SNP. It would also be important to compare AG with AA and GG subjects (also including women) and also those who go on to have events. However, for this considerably larger sample numbers would be required than we have available currently in NPHSII. Furthermore, if heparin is administered to subjects then *LPL, LIPC, LIPG* enzymes would be released and could be measured in plasma.

## Acknowledgments

We gratefully acknowledge expert discussion with Drs Gerhard Liebisch (Regensburg), and Kristin Baldwin (Scripps Research Institute) during the revision of the article. Dr Meckelmann, J.I. Hawksworth, Dr White, P. Rodrigues, Dr Engler, Dr Aldrovandi, Dr Zhou, Dr Alvarez-Jarreta, Dr Andrews, Dr Tyrrell, Dr Hinz, Dr Slatter, Dr Aoki, Dr Lo Sardo, Dr Kano conducted experiments and undertook data analysis. Dr Allen supervised computational tool design. Dr Acharia, J. Cooper, and J. Mitchell collected and processed clinical samples. Drs Meckelmann, O’Donnell, and Humphries conceived the experiments, designed the studies, and drafted the article. All authors edited and approved the article.

## Sources of Funding

This work was supported by the Wellcome Trust (094143/Z/10/Z), British Heart Foundation (RG/12/11/29815), and European Research Council (LipidArrays) to Dr O’Donnell. Dr O’Donnell is a Royal Society Wolfson Research Merit Award Holder and acknowledges funding for LIPID MAPS from Wellcome Trust (203014/Z/16/Z). Dr Humphries acknowledges grant RG008/08 from the British Heart Foundation and the support of the UCLH NIHR BRC.

## Disclosures

None.

## Supplementary Material


