# Supplementary material for: Metabolic Dysregulation of the Lysophospholipid/Autotaxin Axis in the Chromosome 9p21 Gene SNP rs10757274
Source: Circ Genom Precis Med. 2020 May 12;13(3):e002806. doi: 10.1161/CIRCGEN.119.002806 (PMC7299226; doi:10.1161/CIRCGEN.119.002806)

SUPPLEMENTAL MATERIAL

Supplementary Methods

The full dataset is provided as Supplementary Data (supplementary data.xlsx, tab 1).

Patient samples
NPHSII is a prospective CHD study of ~3000 men (20,21)[1, 2]. Briefly, middle-aged men (aged 50–64 yrs) were recruited from 9 general practices in the UK 27 yrs ago. Exclusions included a history of CHD or diabetes. CHD was defined as acute myocardial infarction (MI), silent MI or undergoing coronary surgery. Ethical approval was provided by the National Hospital for Neurology and Neurosurgery and the Institute of Neurology Joint Research Ethics Committee, and Joint UCL/UCLH Committee of Human Research, Committees A and Alpha, and all samples were obtained with informed consent. There was a median of 13.5 years follow-up. Plasma lipids were measured at recruitment using standard methods as described[3]. Samples were selected which had been previously genotyped and shown to be carriers or homozygous for a particular gene variant (Supplementary Table 1) [3, 4]. All plasma used had been stored at -80 ºC since isolation. SNPs were chosen based on known association with altered risk of CHD. For the *APOA5* and *CDKN2A/2B* SNPs, the rare allele is associated with elevated risk of CHD. The rare allele of the *SORT1* and *LDLR* SNPs are associated with lower levels of LDL-C and lower CHD risk. Carriage of the *APOE* E2 allele is associated with decreased risk, and the *APOE* E4 allele with higher risk of both CHD and Alzheimer's disease. Samples selected as controls were non-carriers for all the other selected risk gene variants, and all were homozygous for the *APOE* E3 allele. Thus, they were an appropriate comparison group for the other *APOE* genotype groups and the other SNP genotypes. Details of sample sizes, genes, SNPs and average levels of total triacylglycerides (TAG) and total cholesterol determined for these samples, are provided in Supplementary Table 1. Samples were randomly chosen. Apart from the genotypes mentioned there was no selection or matching of the individuals for any measured criteria. All subjects were non-smokers and were all chosen to be homozygous for the common APOE E3 allele (to avoid possible influence of this genotype). Each respondent attended in the non-fasting state after having been instructed to avoid a cooked breakfast or a heavy meal before examination. They had refrained from smoking and vigorous exercise from midnight beforehand. Therefore, they were not fasting but postprandial to a varying degree. Importantly, the mean (SD) Tg concentration in the NPHSII study cohort is 1.79(1.24)mmol/l which is well within the normal range for men of this age group[5]. Notably, they will not have consumed a substantial amount of fat or carbohydrate, thus limiting postprandial metabolic sequelae. For traditional lipid concentrations, the most likely effect would be to increase variability of the triglyceride measures (compared with fasting) will consequently add "noise" and therefore make associations more difficult to find. Thus, given that we found robust associations, the validity of our results are not in question. We note that our cohort is exclusively male. It is well accepted that the basic vascular physiology of men and women is similar, although there are a number of well reported differences in "traditional" lipid concentrations between men and women, most notable with pre-menopausal women having mean higher concentrations of HDL-C than men, though these differences become smaller post menopause. The relationship between HDL-C and concentrations of the key lipidomic traits we have identified here is unknown, and we have no data to address this.

Global Lipidomics.

In brief, lipids were extracted using two consecutive liquid-liquid extractions, first, hexane:isopropanol:acetic acid, then a modified Bligh and Dyer method as outlined in Supplementary Methods below[6]. Orbitrap datasets were processed using the R version of XCMS (Version 3.4), then using LipidFinder as described in Supplementary Methods [7, 8]. This enabled assignment of a putative lipid class to 30 – 50 % of all ions detected. Our approach to statistical analysis of global datasets is described in full detail below.

The full workflow for global lipidomics was performed in several separate “experiments” to enable regular calibration. Each consisted of samples with a known risk SNP, controls randomly chosen from the cohort, quality controls and blanks. Pooled control plasma was prepared using blood from ten healthy subjects. Samples were randomized before extraction. Control pooled plasma, water blanks and methanol blanks were analysed in a group after every tenth cohort sample. After lipid extraction the samples were analysed in the same randomized order as the extraction using LC-ESI- FTMS in positive and negative mode. A 50 min separation in high-resolution mode was utilised. All major lipid classes, including low abundance species such as fatty acids (FA)/eicosanoids were detected. Lipids were extracted by combining 950 µL water with 50 µL plasma, 4 µl glacial acetic acid and 250 pg internal standards (2H8-arachidonic acid (Cayman Chemicals) and 4ME 16:0 Diether PE) (Avanti Polar Lipids)). Next, 2.5 mL of solvent (1M acetic acid/propan-2-ol/hexane; 2:20:30; v/v/v) was added and samples vortexed for 1 min. Hexane (2.5 mL) was added, vortexed for 1 min and centrifuged for 5 min at 500 g, 4°C. The upper hexane layer was collected. The sample was re-extracted by adding hexane (2.5 mL) to the remaining aqueous phase, vortexing for 1 min and centrifuging for 5 min at 500 g, 4°C. Again, the upper hexane layer was collected and combined with the first hexane layer. The remaining aqueous phase was re-extracted using a modified Bligh and Dyer protocol[6]. 3.75 mL solvent (chloroform/methanol; 1:2; v/v) was added per sample. After vortexing for 1 min., 1.25 mL chloroform was added, and vortexed again for 30 sec., before adding 1.25 mL water, followed by 30 sec. vortex. Samples were centrifuged for 5 min. at 500 g and 4°C and the bottom chloroform layer collected and combined with the two hexane layers before drying under vacuum. Samples were re-suspended in 400 µL methanol and filtered through a centrifuge filter (10 kDa cut off, Millipore) before LC-MS.

Global lipidomics was carried out on an Accela liquid chromatography system coupled to an Orbitrap Elite mass spectrometer (Thermo Fisher Scientific). Liquid chromatographic separation was performed at 30°C using an Accucore C18 (Thermo Fisher Scientific) reversed phase column (150 × 2.1 mm, 2.7 µm) with solvent gradient of mobile phase A (water/acetonitrile; 80/20; v/v; 4 mM ammonium acetate and 0.1 % glacial acetic acid) and B (acetonitrile/isopropanol; 30/70; v/v; 4 mM ammonium acetate and 0.1 % glacial acetic acid) at 0.4 mL/min over 60 min. The linear gradient of B was 16 - 60% for 12 min, 60 - 72% from 12 min - 19 min, 72 - 84% from 19 min - 42 min, 84-98% from 42 min - 51 min and held at 98% for another 6.5 min before re-equilibration. MS conditions were as follows for analysis in positive ESI ionization mode: resolution 60,000 at 400 *m/z* (providing approx. 3 scans/sec) HESI-II temperature 400°C, N2 as drying gas, sheath gas flow 37 arbitrary units (au), auxiliary gas flow 15 au, sweep gas flow 1 au, capillary temperature 320°C, spray voltage + 4.0 kV, S-lens RF level 62 %. Lock mass was *m/z* 391.2843. For analysis in negative ESI ionization

mode: resolution 60,000 at 400 *m/z*, HESI-II temperature 350°C, N2 as drying gas, sheath gas flow 37 au, auxiliary gas flow 15 au, sweep gas flow 2 au, capillary temperature 320 °C, spray voltage – 3.5 kV, S-lens RF level 69 %. Lock mass was *m/z* 265.1479.

Informatics and statistical analysis of global datasets.
These global lipidomic datasets are of significant size requiring specialised informatics. Extensive clean up was used to remove artefacts (adducts, background contaminants, etc), combining positive and negative runs to generate single datasets for comparison between sample sets, and putative identification and assignment to specific lipid classes. Orbitrap datasets were first processed using the R version of XCMS (Version 3.4) for feature detection and alignment[7]. Extracted and aligned features were further processed using LipidFinder to remove isotopes, adducts and background contaminations, with parameters as in Supplementary Methods[8]. No examples of missing values that clustered according to sample type were found, thus we decided that the appropriate approach was to (i) remove any features where >50% of samples were missing values, and random across genotypes(most likely an artefact or below LOD), and (ii) leave missing value cells empty. All metabolites were treated equally.

In addition to using Websearch in LipidFinder, with only curated lipids from LIPID MAPS, we applied an in-house generated retention time and m/z database which identifies around 1,000 lipids known to be in human plasma (level three identification according to the metabolomics standard initiative[9]). The LIPID MAPS database enabled a lipid category to be assigned, with a putative match generated where possible. Settings for the programs used and database lipids are provided in Supplementary Data.xlsx (tab 9, database method). This enabled assignment of a putative lipid class to 30 – 50 % of all ions detected. Next, univariate statistics were applied to identify interesting lipid features (Mann-Whitney u test (non-parametric), calculation of fold change). Chromatograms for all lipids with $p \leq 0.075$ or a high relative fold-change (top 20 % of the features) were manually checked to ensure peak quality, by examining raw data Xcalibur datasets (around 1,500). Next, we processed the data using quantile normalization, applied non-parametric Mann–Whitney U test assuming unequal variance and adjusted the resulting p-values to confirm significance. Quantile normalization was performed using the normalize.quantiles function in the preprocessCore package in R (Version 1.4)[10]. p-Values were adjusted for lipid classes by the sequential goodness of fit approach described in by using the R based version (Version 2.3) [11]. These approaches were applied to lipid categories/sub-classes separately (as seen in Volcano plots) since there is a high degree of correlation structure in the dataset.

Multiple hypothesis testing-corrected approaches are overly punitive for this type of data. Due to this, we instead took the approach of using Mann Whitney U test, adjusting the values for multiple hypothesis testing using a sequential goodness of fit (SGoF) approach as described by Carvajal-Rodriguez et al[11].

Targeted analysis of TGs, CEs and free cholesterol.
Plasma was extracted as for global lipidomics, outlined in Supplementary Methods. LC/MS/MS was performed on a Nexera liquid chromatography system (Shimadzu) coupled to an API 4000 qTrap mass spectrometer (Sciex). Plasma (5 µL) was added to 500 µl water, and 10 µL internal standards (50 ng 2H5-TG(51:1), 500 ng 2H7-CE(18-1)) added. 1.25 mL of solvent (1M acetic acid/propan-2-ol/hexane; 2:20:30; v/v/v) was added and the sample

vortexed for 1 min. Hexane (1.25 mL) was added, vortexed for 1 min and centrifuged for 5 min at 500 g, 4°C. The upper hexane layer was collected. The sample was re-extracted using hexane, vortexed for 1 min and centrifuged for 5 min at 500 g, 4°C. Again, the upper hexane layer was collected and combined with the first. The aqueous phase was then re-extracted using a modified Bligh and Dyer protocol[6]. 1.9 mL solvent (chloroform/methanol; 1:2; v/v) was added. After vortexing for 1 min., 0.6 mL of chloroform was added, and vortexed again for 30 sec. before adding 0.6 mL water, followed by 30 sec. vortexing. Samples were centrifuged for 5 min. at 500 g, 4°C and the bottom chloroform layer collected and combined with the two hexane layers before drying under vacuum, and re-suspending in 400 µL methanol and storing at -80 °C until analysis. Lipid extracts were diluted 1:10 with MeOH. LC-MS/MS for free cholesterol and cholesterol esters and LC-MS analysis of triacylglycerides was performed on a Nexera liquid chromatography system (Shimadzu) coupled to an API 4000 qTrap mass spectrometer (Sciex). Liquid chromatography was performed at 40 °C using a Hypersil Gold C18 (Thermo Fisher Scientific) reversed phase column (100 × 2.1 mm, 1.9 µm) at a flow rate of 0.4 mL/min over 11 min. Mobile phase A was (water/solvent B 95/5; v/v and 4 mM ammonium acetate) and mobile phase B was acetonitrile/isopropanol (40/60; v/v and 4 mM ammonium acetate). The following linear gradient for B was applied: 90 % for 1 min, 90 – 100 % from 1 to 5 min and held at 100 % for 3 min followed by 3 min at initial condition for column re-equilibration. Triglycerides were analysed in selected ion monitoring (SIM) mode covering a range from TG(32:0) up to TG(56:0) including also unsaturated TGs (Supplementary Table 2). MS conditions were: ESI temperature 450°C, N2 as drying gas, ion source gas1 35 psi, ion source gas2 50 psi, curtain gas 35 psi, ESI positive spray voltage 5,0 kV, declustering potential 60 V and entrance potential 10 V. Dwell time was 10 ms resulting in a cycle time of 0.56 sec. TAGs were quantified using an external calibration with the following TG species (TG(14:0-16:1-14:0)-d5, TG(15:0- 18:1-15:0)-d5, TG(16:0-18:0-16:0)-d5, TG(19:0-12:0-19:0)-d5 and TG(17:0-17:1-17:0)- d5). Free cholesterol and CEs were analysed in MRM mode monitoring the parent to daughter transitions of 12 CEs and free cholesterol (Supplementary Table 3). MS conditions were as follows: ESI temperature 150°C, N2 as drying gas, ion source gas1 25 psi, ion source gas2 50 psi, curtain gas 35 psi, ESI positive spray voltage 5.0 kV, declustering potential 70 V, entrance potential 10 V, collision energy 20 V, and collision cell exit potential 25 V. Dwell time was 75 ms for each transition and the cycle time 1.12 sec. cholesterol and CEs were quantified using an external calibration with the following CE standards: CE(14:0), CE(16:0), CE(18:0), CE(18:1), CE(20:4), CE(22:6) and CE(18:1-d7).

Targeted analysis of lysoPLs .

Plasma (50 µL) was added to 950 µl water, and 10 µL internal standards (20 ng LysoPC(18:1-d7) and LysoPE(18:1-d7) and 4µL glacial acetic acid added. 2.5mL of solvent (1M acetic acid/propan-2-ol/hexane; 2:20:30; v/v/v) was added and the sample vortexed for 1 min. Hexane (2.5 mL) was added, vortexed for 1 min and centrifuged for 5 min at 500 g, 4°C. The upper hexane layer was collected. The sample was re-extracted using hexane, vortexed for 1 min and centrifuged for 5 min at 500 g, 4°C. Again, the upper hexane layer was collected and combined with the first. The aqueous phase was then re-extracted using a modified Bligh and Dyer protocol[4]. 3.75 mL solvent (chloroform/methanol; 1:2; v/v) was added. After vortexing for 1 min., 1.25 mL of chloroform was added, and vortexed again for 30 sec. before adding 1.25 mL water, followed by 30 sec. vortexing. Samples were centrifuged for 5 min. at 500 g, 4°C and the bottom chloroform layer collected and combined with the two hexane layers before drying under vacuum, and re-suspending in 400 µL methanol. Samples were

filtered (Amicon® Ultra centrifugal filter units, 10,000 NMWL; Millipore) before storing at -80 °C until analysis. LC-MS/MS was performed on a Nexera liquid chromatography system (Shimadzu) coupled to an API 4000 qTrap mass spectrometer (Sciex). Liquid chromatography was performed at 30 °C using a Accucore C30 (Thermo Fisher Scientific) reversed phase column (100 × 3.0 mm, 2.6 µm) at a flow rate of 0.5 mL/min over 52 min. Mobile phase A was acetonitrile/water (20/80 v/v; 5mM ammonium acetate; 0.1% v/v glacial acetic acid) and mobile phase B was acetonitrile/isopropanol (30/70 v/v; 5 mM ammonium acetate; 0.1% glacial acetic acid). The following linear gradient for B was applied: 60 % for 0.5 min, 60 – 90 % from 0.5 to 15.5 min and held at 90 % for 40 min followed by 10 min at initial conditions for column re-equilibration. LysoPL were analysed in MRM mode monitoring the parent to daughter transitions of 8 lysoPC and 7 lysoPE species (Supplementary Table 4). MS conditions were: ESI temperature 450°C, N2 as drying gas, ion source gas1 40 psi, ion source gas2 30 psi, curtain gas 20 psi, ESI negative spray voltage 4.5 kV. Dwell time was 100 ms resulting in a cycle time of 2.8 sec. Entrance potential was 10 V and collision energy 36 V. Declustering potential was 165 V and 112 V for lyso PC and lyso PE species, respectively. LysoPL were quantified using an external calibration using various species (lysoPC(16:0), lysoPC (18:0), lysoPE(16:0), lysoPE(18:1)) in a concentration range between 0.1-1000 ng/mL. For each lipid, two peaks were often seen and in those cases, both were integrated to give a single value. Most lysoPL are detected as the 1-acyl-2-lyso forms, due to rapid spontaneous acyl migration (the second peak detected)[12]. Examples of chromatograms for lysoPLs are in Supplementary Figure 10

Measurements of LPA

Quantification of LPA was performed according to a previous method with minor modification [13]. 10 µL plasma was mixed with 90 µL methanol containing internal standard (100 nM 17:0-LPA) and then centrifuged at 21.5K x g. The supernatant was filtered using YMC-duo filter (http://www.ymc.co.jp/en/columns/before_disposition/ymc_filter.html). Mobile phase for LC separation: A, Ammonium formate (100 mM) : MQ water = 5 : 95. B, Ammonium formate (100 mM) : ACN = 5 : 95. Gradient was 60 % B for 15 sec, then rising to 100 % B over the following 2.7 min, keeping at 100% B for 2.9 min, then returning to 60 % B over 0.1 min. Flow rate was 200 µL/min, column: Capcell Pak C18 ACR (100 mm × 1.5 mm inner diameter, 3 µm particle size). The system consisted of a Ultimate3000 HPLC and a TSQ Quantiva triple quadropole mass spectrometer (Thermo Fisher, San Jose, CA).Source parameters were 3000V, Sheath Gas 45, Aux Gas 21, Sweep Gas 1, ITP 325 °C, vaporizer temp 275 °C, 0.7 resolution, CID gas 2, cycle time 1 sec. Analysis was conducted in negative ion MRM mode using precursor ions for [M-H]- fragmenting to the phosphate+glycerol fragment remaining after loss of the fatty acyl group (*m/z* 152.9). Examples of chromatograms for lysoPAs are in Supplementary Figure 11

Measurement of ATX.

The ATX levels in the plasma were determined using a two-site immunoenzymatic assay with an ATX assay reagent equipped with an automated immunoassay analyzer, AIA-2000 (Tosoh, Tokyo, Japan) as previously described [14].

Analysis of Affymetrix data from ANRIL down-regulation in cell lines

Raw Affymetrix CEL files relating to total transcript expression in HEK 293 cells stimulated with Tetracycline (shRNA ANRIL silenced for 0h, 48h and 96h) were downloaded from the GEO database (accession: GSE111843) and analysed using packages in CRAN and

Bioconductor: limma, oligo, ggplot2 [15-19]. Data were processed using RMA-normalization and differential gene expression analysis performed using "best practice" detailed in the limma vignette. P-values were corrected for multiple- testing using Benjamini-Hochberg. GO-term enrichment analyses for significant differentially expressed genes (adjusted p-value cut-off: 0.05) were performed using the online PANTHER enrichment tool[20]. The IPA software (QIAGEN Inc.) was used to generate gene/protein interaction networks from genes identified as being significantly differentially expressed (between 96h vs 0h, and 48h vs 0h) and mapping to a common lipid process-associated GO-term. Networks were plotted within IPA using the standard Ingenuity Knowledge Base and default analysis settings. Volcano plots were generated in R using ggplot2.

RNAseq Methods and Analyses for iPSC-derived Vascular Smooth Muscle Cells
iPSCs were created from patient-derived peripheral blood monocytes, edited to selectively delete the locus, differentiated, and lysed, prepared, and run on an Illumina HiSeq2500 to obtain transcriptomic expression data from RNA-sequencing as reported by Lo Sardo and coworkers, available at GEO (GSE120099) [21]. Data was normalized using variance stabilizing transformation and differentially expressed genes (DEGs) were identified using DESeq2 R package (v1.8.2)[22]. Data was selectively clustered using lipid enzyme genes reported in Supplementary Data.xlsx, tab 8, by MORPHEUS[23], employing hierarchical cluster with Pearson correlation to identify DEGs with common expression patterns. These data were further analysed for principal components using ClustVis in R[24]. Data is grouped and reported by patient genotype for the non-risk haplotype (NNWT), risk haplotype (RRWT) and their genome edited counterparts (NNKO and RRKO). DEGs where RRWT is lower than the other three genotypes were identified, and significance assessed by Mann Whitney U test. Pearson correlation coeffecients (r) and p-values were determined using AnswerMiner (https://www.answerminer.com/calculators/correlation-test/).

Analysis of databases for miRNAs that potentially link ANRIL with target lysoPL metabolism genes. TarBase v8 (http://carolina.imis.athena-innovation.gr/diana_tools/web/index.php?r=tarbasev8%2Findex) and TargetScan (*http://www.targetscan.org/vert_72/) were downloaded and searched for experimentally-determined and in silico predicted interactions between the lysoPL metabolism genes and the target mirs identified in [25].

Statistics.
Targeted data are shown as Tukey box plots, * $p < 0.05$, ** $p < 0.01$, *** $p < 0.005$, Mann Whitney U and Student's t-test were used as described in legends. Correlation analysis was undertaken using Answerminer (https://www.answerminer.com/calculators/correlation-test), using Pearson's correlation coefficient. To compare the slopes (or "Pearson correlation coefficients") of regression lines between AA and GG carriers, we used the method described [26].

XCMS parameters and R-script

```
# all samples

setwd("C:/myData")

myClass1 <- "c"

myClass2 <-"sol"


# peak picking using wavelet algorithm for peak detection (centWave)

xset <- xcmsSet (method="centWave",ppm=10, peakwidth=c(10,120), snthresh=5,
prefilter=c(10,20000), integrate=1, mzdiff=0.001, fitgauss=FALSE,  noise=20000,
scanrange=c(1,11485))


# peak alignment

xset <- group(xset, bw=30, mzwid=0.005, minfrac=0.5, minsamp=1)


# retention time correction

xset <- retcor(xset, method="obiwarp", profStep=0.05, response=20, center=1
,plottype="deviation")


#re-align

xset <- group(xset, bw=3, mzwid=0.005, minfrac=0.5, minsamp=1)


# fill in missing peak data

xset <- fillPeaks(xset)


# output results

reporttab <- diffreport(xset, filebase="output")
```

LipidFinder Parameters

Parameter	Parameter descritption	Expected Data Type	Current value
firstRepOffset	This is the index of the first sample, first replicate column	Integer(>3)	3
numberOfSamples	The number of samples in the experiment	integer (>0)	49
numberOfTechReps	The number of replicates of each sample	integer (>0)	1
numberOfQCReps	The number of QC replicates in the input file(s)	integer (>=0)	0
numberOfSolventReps	The number of solvent replicates in the input file(s)	integer (>=0)	5
filePolarityMode	File Polarity Mode (P: Positive mode files		
columnType	The type of column used for LC (PO: Polar		
QCLowRSD	Lower relative standard deviation cut off	integer (>0 AND <QCHighRSD)	30
QCHighRSD	upper relative standard deviation cut off	integer (>QCLowRSD AND <100	50
removeSolvent	Solvent removal toggle (TRUE: Remove solvent intensity		
solventFoldCutOff	The minimum fold difference greater than the solvent intensity a sample replicate intensity must be in order to be considered significant enough to process further	Float (>0.0)	3
intensitySignificanceCutOff	The level at which the intensity of a sample reading is significant enough to process	Integer (>0)	1
mzFixedError	The fixed error allowable when observing a mass	Float (>0.0)	0.0005
mzSizeErrorPPM	The mass size dependant PPM error to add to the fixed error	integer (>0)	4
peakMaxRTWidth	The maximum allowable retention time (mins) a single lipid peak can span	Float (>=3*peakAdjacentFrameMaxRT)	0.003

Parameter	Parameter descritption	Expected Data Type	Current value
peakMinFoldCutOff	The minimum fold difference greater than the adjacent candidate frame's intensity that the current frame intensity must be in order for the current frame to be considered part of a peak.	Float (>1.0)	1.3
peakAdjacentFrameMaxRT	The maximum time difference (mins) between a feature edge and an adjacent frame where the adjacent frame could be considered for inclusion in the same feature	Float(<=peakMaxRTWidth/3)	0.001
peakConcatenateAllFrames	This toggle indicates which peak concatenation should be performed (TRUE:Concatenate all peak frame intensities into the peak centre, FALSE:Concatenate only the most intense peak frame into the peak centre)	boolean (True or False)	TRUE
removeContaminant	This toggle allows the user to specify whether contaminant removal should be executed (TRUE: Remove contaminants		
removeAdduct	This toggle allows the user to specify whether adduct removal should be executed (TRUE: Remove adducts		
adductAddition	Adduct addition toggle. Allows the user to specify whether they wish to add the intensity of any adducts identified to the intensity of the primary mass (True: Add adduct intensity to primary mass intensity		
removeStack	This toggle allows the user to specify whether stack removal should be executed (TRUE: Remove stacks		
maxStackGap	The maximum number missing values before a stack search will terminate for a particualr lipid or contaminant	integer (>=0)	3
lipidStackAddition	Lipid stack addition toggle. Allows the user to specify whether they wish to add the intensity of any lipid stacks ions identified to the intensity of the primary mass (True: Add adduct intensity to primary mass intensity		
rtTolMultipler	A multiplier for peakAdjacentFrameMaxRT to allow a smaller tolerance in certain circumstances (e.g. when looking for stacks)	Float (<=1.0)	1
outlierHighIntensityValue	The cut off point of the replicate means of a sample between using the lower RSD cut off (outlierLowIntensityRSD) and the higher RSD cut off (outlierHighIntensityRSD)	integer (>0)	5000
outlierLowIntensityRSD	The RSD to use when the mean average intensities are lower than the replicate mean intensity cut off (repMeanOutCorCutOff)	integer (>0 AND <outlierHighIntensityRSD)	35
outlierHighIntensityRSD	The RSD to use when the mean average intensities are higher than the replicate mean intensity cut off (repMeanOutCorCutOff)	integer (>outlierLowIntensityRSD)	40
featureLevelMassAssignment	This toggle allows the user to specify whether they wish to assign the masses of every frame to the highest intesity mass in the feature set, default is assignment at the mass group level (TRUE: Re-assign every		

Parameter	Parameter descritption	Expected Data Type	Current value
	mass in a feature group to the mass of the highest intensity frame within the feature group		
negativeModeAdductPairs	The pairs of negative adducts relating to the index in the adducts.csv file	2d list of indices example format [[0,1],[0,2],[3,4],_]	[[0,1],[0,2],[0,3],[0,4],[2,3]]
positiveModeAdductPairs	The pairs of positive adducts relating to the index in the adducts.csv file	2d list of indices example format [[0,1],[0,2],[3,4],_]	[[5,6],[5,7],[5,8],[5,9],[5,10],[6,7],[6,10],[8,9]]
broadContsdMult			1
broadContminPoints			4
broadContRSDCutOff			30
broadContrtSDCutOff			2
retentionTimeLowCutOff			1.3
retentionTimeHighCutOff			56
rtCorrectStDev			999
rtCorrectMeans			FALSE

Supplementary Results

Targeted MS analysis of triglycerides (TG), cholesterol and cholesteryl esters (CE) shows no change in profile between genotypes

Traditional cardiovascular risk factors were measured many years ago for the NPHSII cohort. Overall, total cholesterol, TG and CE tended to be lower for this GG risk group (Supplementary Table 1). This is a reflection of the control AA group randomly selected, which overall had basal lipid levels at the upper range of normal, since large cohort studies have shown that this risk variant is not associated with changes in circulating lipoproteins (1, 9). However, clinical measurements do not include the large number of molecular species of both TG and CE. Herein, quantitative MS analysis found that the relative abundance of these across genotypes is similar (Supplementary Figure 12). Lipidomics of TG generally reports single "species" based on molecular weight, which describes the total number of carbons and double bonds in the FAs, e.g. TG(52:4)[27]. Thus, we also interrogated individual TG peaks eluting within each chromatogram for genotype-specific changes, but none were found (data not shown). Thus, the profile of individual CE and TG molecular species are not altered in the GG risk genotype.

Global lipidomics analysis of plasma

Here, we used an untargeted analysis to generate a hypothesis which we next addressed using fully-validated gold standard quantitative targeted methods as a second step. The LipidFinder approach provides an extensive but unvalidated dataset, considered analogous to a gene array (Figure 1 A). This required extensive method development for plasma samples, outlined in full below. Since high resolution MS is not quantitative and does not fully identify the lipid structures being measured, we instead assigned ions to lipid categories, and then statistically analysed the categories separately to examined for SNP-dependent differences. We then followed up further, measuring again the lipids of interest in a targeted assay, then again using a second independent set of samples to increase statistical power, as described[28].

(i) Optimization of the workflow. XCMS was used for initial processing, however this is not designed for deep mining to detect "unknown" lipids, and was unable to remove many artefacts. Post-XCMS, around 14,000 ions are retained in the dataset, far more than would be considered representative of true lipids (Figure 1 A). When LipidFinder was then used for automatic data clean-up and removal of duplicate ions in both positive and negative ion modes, the number reduced to around 10,000. This was followed by isotope removal, then a manual clean up including intensity cut-off and missing value analysis. Features with > 50 % missing values equally distributed across all samples were considered as artefacts/below LOD and removed. Next, extracted ion chromatograms (EIC) were generated for all ions with p-values ≤ 0.075 and/or high fold-change (top 10 % of features with highest change in either positive or negative mode) and corrected for noisy/abnormal peak shape/spikes. Any peaks not matching the quality criteria (> 3-times background level, Gaussian peak shape and <10 data points per peak) were removed. These steps further reduced the number of ions to around 2,000 - 3,000 per sample. Scatter diagrams show the difference between data obtained post-XCMS, then after LipidFinder and manual clean- up steps, including WebSearch and in-house database search (Figure 1 A,B). Major lipid categories were assigned according to the LIPID MAPS classification system. As expected for reverse phase chromatography, glycerophospholipids (GPL, green) elute before more non-polar

glycerolipids (GL, including TGs, DGs, red) and sterols (including CEs, pink). The most abundant lipids detected were glycerophospholipids (GPLs, including phospholipids (PL) and lysoPL (lysoPL)) accounting for ~21%, followed by glycerolipids (GL, including diglycerides (DG) and triacylglyceridesTG, 12%), sphingolipids (SL, 9%), fatty acyls (FA, ~3%), sterol lipids (~1.4%) and prenol lipids (<1%). Unknowns accounted for 53%. An example sample analysis is shown in Figure 1 B.

(ii) Assay performance

Throughout the experiment total ion current (TIC) chromatograms for QC samples were visually compared, since these originate from a single pooled plasma sample. Also, extracted ion chromatograms (EIC) of several known lipids were compared for chromatographic peak shape and mass accuracy across all QC samples. % CV was calculated for all features and those with % CV higher than 50% were removed from the dataset. For example, average % CV across all features for the QC samples of the *CDKN2A/2B* dataset was 17.5 % (n = 7). In addition, % CV was calculated for 15 lipid classes (one lipid per class). As an example, Figure shows boxplots of these 15 lipids for the QC samples (Supplementary Figure 13). Here, % CV ranged from 5.7 % for γ-linolenic acid up to 28.9 % for PS(38:1), comparable with previous studies[9].

(iii) Storage considerations

A random set of NPHSII controls was compared with plasma from genetically-unrelated healthy donors obtained in the present day (n = 10 for both) using untargeted MS. Total ion current (TIC) for NPHSII versus fresh samples was found to be similar (Supplementary Figure 1 A). Relative abundance of selected lipids from different classes was then compared[29]. The majority were not significantly different and likely represent normal variation in unrelated human subjects (Supplementary Figure 1 B). Also, several TGs and CEs were separately compared using a targeted assay, and only 1 of 17 was significantly lower in NPHSII samples (Supplementary Figure 2 A,B). Since oxidation during storage could lead to artefactual generation of bioactive species, we measured four non-enzymatically-oxidized PLs using targeted MS and found these to be considerably higher in the older samples (Supplementary Figure 2 C). Last, we measured lysoPC signals from the untargeted dataset and found that there were some increases, although not all were significant (Supplementary Figure 3). Thus, for most lipid classes, plasma that has been correctly stored for many years is usable. In the case of oxidized lipids and lysoPLs the impact of changes during storage need to be considered, including whether differences in lysoPLs could result from altered levels of plasma lipase enzymes inherent in plasmas from different patient groups (this is discussed in more detail below).

Statistics considerations for targeted datasets

In targeted assays, lysoPLs were in general normally distributed (Kolmogorov-Smirnov test) or near normal, while ATX was normal. For both lysoPLs and ATX, we include both t-tests (black) and Mann Whitney U (red) on our box and whisker plots, and note that we get virtually identical results with either test (in some cases, the Mann Whitney U test returns higher levels of significance for AA versus GG). To further examine the issue of statistical power, we conducted a power calculation for all variables using GPower[30]. These were performed for a t-test with alpha = 0.05 and power at 80%. Minimum sample numbers returned varied from 38-71 for lysoPCs (e.g. 16:0 - 44, 18:0 - 38, 18:1 - 44, 18:2 - 71 for lysoPCs), and 23 for ATX.

Generally, non-parametric tests would require up to 15% more, so the sample sizes for lysoPCs and ATX were sufficient.

For lysoPA, we first analysed the second validation cohort (just under 50 samples per group). However, our power calculation indicated numbers were required as follows: 14:0 – 65, 16:0 – 52, 16:1 – 53, 18:0 – 81, 18:1 – 69, 18:2 – 60, 18:3 – 201, 20:3 – 36, 20:4 – 39, 20:5 – 110, 22:6 – 3000). Furthermore, lysoPAs were not normally distributed since they had some values that tended to be considered “outliers” on the higher side of the median. This was more obvious for AA than GG samples in general. To address this, we analysed a second cohort of samples from different individuals, bringing the total number of samples to 95,100 (AA,GG), and providing sufficient statistical power for 8 out of 11 lysoPAs. It is well accepted that t-tests can be applied to non-normal data, so long as the sample size is sufficiently large. There is debate in the literature on what sample size would be considered sufficient with estimates from 15 up to 80 in various papers [31]. As we have 95-100 for our groups, we have applied both t-test and Mann Whitney U test. We found that using t-test, that 7 of 11 lysoPAs are significantly lower in the GG risk group, while we did not see significant differences using Mann Whitney U. Thus, overall lysoPAs were lower, but extent of the reduction was relatively small.

Consideration of sample storage and processing relating to lipid levels.
We took significant efforts to ensure that the cohort plasma was of sufficient quality. In comparison with fresh plasma, we found that most lipids were unchanged, however oxidized phospholipids and lysoPCs were somewhat increased during the time in storage (lysoPA is not detectable using untargeted lipidomics). In the case of NPHSII samples used herein, all samples were centrifuged at 1000 x G for 10 min at room temperature before immediate low temperature storage. This will have prevented their generation during initial sample processing. However, it is likely that during the 25 yrs of storage, some generation of these lipids will have occurred at a low rate. Although we could not measure this in the Orbitrap data, similar elevations in lysoPA may have occurred, as Liebisch has also seen that these lipids also rise with storage of plasma at room temperature[32].

Since lysoPC appears to have increased in storage, the altered levels we found in AA vs GG may have been generated during low temperature storage. However, the AA and GG plasma is different in relation to both lysoPC and lysoPA and their underlying metabolism, and elucidating why is important in terms of revealing the underlying vascular lipidomic impact of this SNP. Our observations need to be considered a starting point for further studies to fully delineate the biological processes involved. We also point out that our findings are relevant for others undertaking cohort studies some of which are uncovering similar alterations in lysoPC being associated with future risk of cardiovascular disease and visceral obesity[27, 33, 34]. In those cases, and for future studies it needs to be understood that these lipids can alter during storage even at low temperatures, and further work is required to understand the mechanistic basis of this and properly interpret cohort findings in light of this information.

We compared our data with the Bruneck cohort[27]. Findings there were similar to us (reduced lysoPC associated with CVD risk), but that study looked at prospective risk of an event, not genetic risk. In that case, most lysoPCs (unlike pretty much all other lipid classes they looked at) were inversely associated with CVD risk but only 3 achieved nominal statistical significance of $p<0.05$. None maintained significance after adjustment for multiple testing by

means of the Benjamini-Hochberg procedure. However, using the three different selection procedures (Lasso, best subset, stepwise), LysoPCs were frequently selected and adding them to the lipid panel improved the C-index and Continuous net reclassification index (Manuel Mayr, personal communication). Thus, LPCs appeared to provide additional information compared to the other lipid classes that are mostly positively associated with CVD risk. This finding shows that a relatively small reduction in lysoPC is a consistent finding associated with risk. The observation that lysoPC measurement can add value to predictions of CVD risk strongly suggests a genetic component is involved and that they are pathophysiologically relevant even with a modest change. We propose that the genetic component may be accounted for by their association with a common CVD risk SNP present in up to 30% of the population.

Validation and replication of findings.
In this study, we conducted several forms of validation using different assays, and also additional samples from the NPHSII cohort as follows: our initial study (untargeted) was conducted on 33 and 39, from GG and AA allele carriers, respectively. Following this, the same samples were analysed again for lysoPLs, using the gold standard targeted quantitative assay. This confirmed the same result in two separate assays. A second set (GG:49, AA:47) of new samples was then obtained and lysoPL measured using the targeted assay. Both sample sets showed the same results, that overall lysoPLs (lysoPCs primarily) are reduced in GG. This data was combined to generate Figure 2 A, but both sets are shown also separately as Supplementary Figure 4 A,B. LysoPAs were first measured in the second set (GG: 49, AA:47), and some were significantly lower. We then increased sample numbers up to 95-100 for each genotype, and showed that overall 7 of 11 were significantly decreased. In that case, increasing sample numbers didn't change outcome significantly, so the data shown in Figure 4 D represents both sample sets combined.

Additional information on some regulated genes from the HEK dataset.
In HEK cells, downregulation of *PNPLA2* (*ATGL*, a lipase) and *PLA2G4C* (PLA_2Group IVC, a PLA_2 strongly expressed in artery and heart) is consistent with our lipidomics findings of decreased lysoPL (Table 3) [35, 36] (Supplementary Data.xlsx, tab 7, Table 3, Scheme 1).
We also tested for upregulation of potential lysoPL removal pathways following *ANRIL* knockdown. LysoPL can be metabolized by *PLBD1* (Phospholipase B Domain Containing 1, expressed in neutrophils), removing the phospholipid headgroup, and this was significantly elevated in GG (Table 3, Scheme 1) [37]. LysoPL can also be recycled back into PL pools via Land's cycle enzymes, and consistent with this, upregulated *LPCAT2* (PC-acyl transferase in blood cells) and *MBOAT2* (a PE-acyl transferase in neutrophils) were seen (Table 4, Scheme 1) [35, 38, 39]. Also, significant upregulation of *ACSL6*, a long chain acyl-CoA synthetase expressed in leukocytes and erythrocytes, required for fatty acid re-acylation was noted (Table 3, Scheme 1) [35, 40-42]. *ACSL6* works in concert with *LPCAT2* and *MBOAT2*. The data with MBOAT2 is interesting since its expression increased in both cell types in association with "risk", and it has been recently associated with elevated T2D risk[43]. This enzyme reduces lysoPLs by esterifying them to fatty acyl-CoAs and is known to be expressed in whole blood cells, including leukocytes.

GG plasma showed significantly lower lysoPAs (Figure 2 D). These can be removed by phospholipid phosphatases, including *PLPPR2*, *PLPP1* (expressed in platelets), or *PLPP2* (in lung), and all were significantly induced by ANRIL knockdown [44, 45].

Additional genes identified in the VSMC data include PLA2G12A (GXII sPLA2 expressed by several cell types including blood cells), *LCAT* (a plasma enzyme generated in liver that converts cholesterol and PC to cholesteryl esters and lysoPCs), *LPL* (lipoprotein lipase, generated in liver and tightly bound to vascular endothelium. Hydrolyses complex lipids to generate lysoPC), *PLPP1* (phospholipid phosphatase1, in platelets, where it hydrolyses lysoPA), *DGKA* (diacylglycerol kinase, can convert lysoPA to monoglycerides, in lymphocytes).

In summary, our *in vitro* analysis provided several new candidates for reducing lysoPL/lysoPA in the context of Chr9p21-mediated CHD risk. Based on their known cellular localization, potential sources are proposed (Supplementary Figure 5 C).

Table and Figure legends

Supplementary Table 1. Summary of genes, SNPs, sample numbers and recruitment lipid levels used in the untargeted analysis. $ All subjects were homozygous for the common alleles for all other selected SNPs. * $p < 0.05$, ** $p < 0.01$, *** $p < 0.005$, 2-tailed, unpaired Student's T-test, significantly different to APOE E3E3 controls.

Supplementary Table 2: MS parameters for analysing triacylglycerols by selected ion monitoring using the API 4000 (Sciex) platform

Supplementary Table 3: MS parameters for analysing free cholesterol and cholesterol esters by MS/MS using the API 4000 (Sciex) platform.

Supplementary Table 4: MS parameters for analysing lysoPLs by MS/MS using the API 4000 (Sciex) platform.

Supplementary Figure 1. Comparison of fresh and cohort samples shows similar lipidomic results. *Panel A. Total ion counts (TICs) of freshly-drawn plasma samples and controls from the Northwick Park Heart Study II (NPHSII) are similar.* Total ion current for each sample was integrated for the whole of the time of elution (n = 10 for old and new samples), and is shown as Tukey box plots. *Panel B. Integrated peak areas were compared for 19 lipids and are similar for fresh and cohort plasma.* Lipids were compared in fresh plasma or NPHSII samples (n = 10 for both) and are shown as Tukey box plots. * $p < 0.05$, ** $p < 0.01$, *** $p < 0.005$, 2-tailed, Mann Whitney U

Supplementary Figure 2. CEs and TGs are similar between cohort and fresh plasma samples, but oxPL are significantly increased in storage. *Panel A.* CEs were analysed in fresh human plasma and from NPHSII control samples by targeted lipidomics (n = 10 for both), shown as Tukey box plots. *Panel B*. TG molecular species were analysed in fresh human plasma and from NPHSII control samples by targeted lipidomics (n = 10 for both), shown as Tukey box plots. Panel C. oxPL were analysed in fresh human plasma and from NPHSII control samples by targeted lipidomics (n = 10 for both), shown as Tukey box plots. * $p < 0.05$, ** $p < 0.01$, *** $p < 0.005$, 2-tailed, Mann Whitney U

Supplementary Figure 3. Integrated peak areas were compared for lysoPCs and were higher in cohort plasma. Lipids were compared in fresh plasma or NPHSII samples (n = 10

for both) and are shown as Tukey box plots. * $p < 0.05$, ** $p < 0.01$, *** $p < 0.005$, 2-tailed, unpaired Student's T-test

Supplementary Figure 4. Separate analysis of two sets of samples confirms reduced lysoPLs in GG samples versus AA. *Panel A. LysoPLs were analysed using LC/MS/MS as in Methods in AA (39) and GG (33) samples.* The sample set used for global LipidFinder analysis was first compared using a targeted assay. *Panel B. Confirmation of decreased lysoPLs in a second sample set.* An additional set of samples of each genotype (AA: 47, GG: 49) were analysed using LC/MS/MS, as described in Methods. * $p < 0.05$, ** $p < 0.01$, *** $p < 0.005$, 2-tailed, unpaired Student's T-test (black) or Mann Whitney U (red)), shown as Tukey box plots.

Supplementary Figure 5. Significant changes in lipid regulatory gene expression are observed with ANRIL knockdown in cell culture. Volcano plots showing differential gene expression of all genes on the Affymetrix HuGene1.0 v1, chip. The horizontal dashed line shows where adj.pvalue < 0.05 (Benjamini-Hochberg correction) where points (genes) above this line are significantly differentially expressed. LysoPL regulating genes that alter in line with decreased levels of the lipids are labelled in black. Genes in red are annotated to the GO-term detailed in the plot title. Data are plotted in R using ggplot2. These volcano plots show additional GO terms that were significantly regulated in addition to those in Figure 5 of the main text.

Supplementary Figure 6. Datasets for lysoPL metabolizing genes showing that removing the risk locus reverts gene expression back to levels in non-risk individuals. * $p < 0.05$, ** $p < 0.01$, *** $p < 0.005$, Students t-test.

Supplementary Figure 7. Expression of ANRIL isoforms in iPSC-derived VSMCs from carriers of either risk (RRWT) or non-risk alleles (NNWT) in Chr9p21, and isogenic knock out lines (RRKO, NNKO). Graphs show expression level of ANRIL by RT-qPCR. PPIA (Cyclophilin A) was used as housekeeping gene. Expression level of different ANRIL isoforms was evaluated by using primers detecting junctions between exons 6-7 (present in short and long isoforms) and exons 18-19 (present only in long ANRIL isoforms). Graphs show mean ± 95% CI. ***$p < 0.0001$ one-way ANOVA-Bonferroni.

Supplementary Figure 8. Correlation analysis using AnswerMiner shows significant positive or negative correlations with expression of ANRIL (exons 6-7) for several lysoPL metabolising genes. Gene expression data from Lo Sardo *et al* was compared, using Pearson correlations [21]. Non-risk haplotype (NNWT), risk haplotype (RRWT) and their genome edited counterparts (NNKO and RRKO) are shown, n = 9-10 clones per group.

Supplementary Figure 9. Expression of ANRIL isoforms in VSMC cell lines, and correlation of ANRIL (exons 18-19) with several lysoPL metabolising genes. Gene expression data from Lo Sardo et al was compared, using Pearson correlations [21]. Non-risk haplotype (NNWT), risk haplotype (RRWT), n = 9-10 clones per group. Genome edited counterparts are not used since ANRIL (18-19) has been removed.

Supplementary Figure 10. Example chromatograms for lysoPC and lysoPEs measured using a targeted LC/MS/MS assay (Cardiff).

Supplementary Figure 11. Example chromatograms for lysoPAs measured using a targeted LC/MS/MS assay (Tokyo)

Supplementary Figure 12. The profiles of TG or CE molecular species are not altered in the rs10757274 GG genotype. TG or CE were measured using LC/MS/MS as outlined in Methods. Heatmaps of normalized mean values for AA and GG are shown.

Supplementary Figure 13. Calculation of variance for assay performance, based on data obtained from Orbitrap data. Boxplots of 15 abundant representative lipids detected in QC samples (n = 7). y-Linolenic acid, LPC(18:2), PI(36:1), PS(38:1), and CER(42:0) were identified in ESI negative mode and LPE(18:0), PC(36:2p)/(36:3e), PE(38:5), PA(38:2), PG(40:6), SMpe(43:0), SMpc(40:1), hexCER(36:2), DG(34:2) and TG(50:4), and %CV calculated, as shown on Tukey box plots, where box represents interquartile range and line represents median.

References

1. Cooper JA, Miller GJ and Humphries SE. A comparison of the PROCAM and Framingham point-scoring systems for estimation of individual risk of coronary heart disease in the Second Northwick Park Heart Study. *Atherosclerosis*. 2005;181:93-100.
2. Miller GJ, Bauer KA, Barzegar S, Foley AJ, Mitchell JP, Cooper JA and Rosenberg RD. The effects of quality and timing of venepuncture on markers of blood coagulation in healthy middle-aged men. *Thromb Haemost*. 1995;73:82-6.
3. Humphries SE, Cooper JA, Talmud PJ and Miller GJ. Candidate gene genotypes, along with conventional risk factor assessment, improve estimation of coronary heart disease risk in healthy UK men. *Clin Chem*. 2007;53:8-16.
4. Drenos F, Talmud PJ, Casas JP, Smeeth L, Palmen J, Humphries SE and Hingorani AD. Integrated associations of genotypes with multiple blood biomarkers linked to coronary heart disease risk. *Hum Mol Genet*. 2009;18:2305-16.
5. Miller GJ, Bauer KA, Barzegar S, Cooper JA and Rosenberg RD. Increased activation of the haemostatic system in men at high risk of fatal coronary heart disease. *Thromb Haemost*. 1996;75:767-71.
6. Bligh EG and Dyer WJ. A rapid method of total lipid extraction and purification. *Canadian journal of biochemistry and physiology*. 1959;37:911-7.
7. Smith CA, Want EJ, O'Maille G, Abagyan R and Siuzdak G. XCMS: processing mass spectrometry data for metabolite profiling using nonlinear peak alignment, matching, and identification. *Anal Chem*. 2006;78:779-87.
8. O'Connor A, Brasher CJ, Slatter DA, Meckelmann SW, Hawksworth JI, Allen SM and O'Donnell VB. LipidFinder: A computational workflow for discovery of lipids identifies eicosanoid-phosphoinositides in platelets. *JCI Insight*. 2017;2:e91634.

9. Sumner LW, Amberg A, Barrett D, Beale MH, Beger R, Daykin CA, Fan TW, Fiehn O, Goodacre R, Griffin JL, et al. Proposed minimum reporting standards for chemical analysis Chemical Analysis Working Group (CAWG) Metabolomics Standards Initiative (MSI). *Metabolomics*. 2007;3:211-221.
10. Blekhman R, Perry GH, Shahbaz S, Fiehn O, Clark AG and Gilad Y. Comparative metabolomics in primates reveals the effects of diet and gene regulatory variation on metabolic divergence. *Sci Rep*. 2014;4:5809.
11. Carvajal-Rodriguez A, de Una-Alvarez J and Rolan-Alvarez E. A new multitest correction (SGoF) that increases its statistical power when increasing the number of tests. *BMC Bioinformatics*. 2009;10:209.
12. Pluckthun A and Dennis EA. Acyl and phosphoryl migration in lysophospholipids: importance in phospholipid synthesis and phospholipase specificity. *Biochemistry*. 1982;21:1743-50.
13. Okudaira M, Inoue A, Shuto A, Nakanaga K, Kano K, Makide K, Saigusa D, Tomioka Y and Aoki J. Separation and quantification of 2-acyl-1-lysophospholipids and 1-acyl-2-lysophospholipids in biological samples by LC-MS/MS. *J Lipid Res*. 2014;55:2178-92.
14. Nakamura K, Igarashi K, Ide K, Ohkawa R, Okubo S, Yokota H, Masuda A, Oshima N, Takeuchi T, Nangaku M, et al. Validation of an autotaxin enzyme immunoassay in human serum samples and its application to hypoalbuminemia differentiation. *Clin Chim Acta*. 2008;388:51-8.
15. Bochenek G, Hasler R, El Mokhtari NE, Konig IR, Loos BG, Jepsen S, Rosenstiel P, Schreiber S and Schaefer AS. The large non-coding RNA ANRIL, which is associated with atherosclerosis, periodontitis and several forms of cancer, regulates ADIPOR1, VAMP3 and C11ORF10. *Hum Mol Genet*. 2013;22:4516-27.
16. Barrett T, Wilhite SE, Ledoux P, Evangelista C, Kim IF, Tomashevsky M, Marshall KA, Phillippy KH, Sherman PM, Holko M, et al. NCBI GEO: archive for functional genomics data sets--update. *Nucleic Acids Res*. 2013;41:D991-5.
17. Carvalho BS and Irizarry RA. A framework for oligonucleotide microarray preprocessing. *Bioinformatics*. 2010;26:2363-7.
18. Ritchie ME, Phipson B, Wu D, Hu Y, Law CW, Shi W and Smyth GK. limma powers differential expression analyses for RNA-sequencing and microarray studies. *Nucleic Acids Res*. 2015;43:e47.
19. ggplot2: Elegant Graphics for Data Analysis [computer program]. New York: Springer-Verlag; 2009.
20. Mi H, Muruganujan A, Ebert D, Huang X and Thomas PD. PANTHER version 14: more genomes, a new PANTHER GO-slim and improvements in enrichment analysis tools. *Nucleic Acids Res*. 2019;47:D419-D426.
21. Lo Sardo V, Chubukov P, Ferguson W, Kumar A, Teng EL, Duran M, Zhang L, Cost G, Engler AJ, Urnov F, et al. Unveiling the Role of the Most Impactful Cardiovascular Risk Locus through Haplotype Editing. *Cell*. 2018;175:1796-1810 e20.
22. Love MI, Huber W and Anders S. Moderated estimation of fold change and dispersion for RNA-seq data with DESeq2. *Genome Biol*. 2014;15:550.
23. Morpheus. *ps://softwarebroadinstituteorg/morpheus*.
24. Metsalu T and Vilo J. ClustVis: a web tool for visualizing clustering of multivariate data using Principal Component Analysis and heatmap. *Nucleic acids research*. 2015;43:W566-70.
25. Kong Y, Hsieh CH and Alonso LC. ANRIL: A lncRNA at the CDKN2A/B Locus With Roles in Cancer and Metabolic Disease. *Front Endocrinol (Lausanne)*. 2018;9:405.

26. Wuensch KL. Comparing correlation coeffecients, slopes and intercepts. 2007.
27. Stegemann C, Pechlaner R, Willeit P, Langley SR, Mangino M, Mayr U, Menni C, Moayyeri A, Santer P, Rungger G, et al. Lipidomics profiling and risk of cardiovascular disease in the prospective population-based Bruneck study. *Circulation*. 2014;129:1821-31.
28. Skol AD, Scott LJ, Abecasis GR and Boehnke M. Joint analysis is more efficient than replication-based analysis for two-stage genome-wide association studies. *Nat Genet*. 2006;38:209-13.
29. Quehenberger O, Armando AM, Brown AH, Milne SB, Myers DS, Merrill AH, Bandyopadhyay S, Jones KN, Kelly S, Shaner RL, et al. Lipidomics reveals a remarkable diversity of lipids in human plasma. *J Lipid Res*. 2010;51:3299-305.
30. Faul F, Erdfelder E, Lang AG and Buchner A. G*Power 3: a flexible statistical power analysis program for the social, behavioral, and biomedical sciences. *Behav Res Methods*. 2007;39:175-91.
31. Lumley T, Diehr P, Emerson S and Chen L. The importance of the normality assumption in large public health data sets. *Annu Rev Public Health*. 2002;23:151-69.
32. Scherer M, Schmitz G and Liebisch G. High-throughput analysis of sphingosine 1-phosphate, sphinganine 1-phosphate, and lysophosphatidic acid in plasma samples by liquid chromatography-tandem mass spectrometry. *Clin Chem*. 2009;55:1218-22.
33. Fernandez C, Sandin M, Sampaio JL, Almgren P, Narkiewicz K, Hoffmann M, Hedner T, Wahlstrand B, Simons K, Shevchenko A, et al. Plasma lipid composition and risk of developing cardiovascular disease. *PLoS One*. 2013;8:e71846.
34. Syme C, Czajkowski S, Shin J, Abrahamowicz M, Leonard G, Perron M, Richer L, Veillette S, Gaudet D, Strug L, et al. Glycerophosphocholine Metabolites and Cardiovascular Disease Risk Factors in Adolescents: A Cohort Study. *Circulation*. 2016;134:1629-1636.
35. Carithers LJ, Ardlie K, Barcus M, Branton PA, Britton A, Buia SA, Compton CC, DeLuca DS, Peter-Demchok J, Gelfand ET, et al. A Novel Approach to High-Quality Postmortem Tissue Procurement: The GTEx Project. *Biopreserv Biobank*. 2015;13:311-9.
36. Rowley JW, Oler AJ, Tolley ND, Hunter BN, Low EN, Nix DA, Yost CC, Zimmerman GA and Weyrich AS. Genome-wide RNA-seq analysis of human and mouse platelet transcriptomes. *Blood*. 2011;118:e101-11.
37. Xu S, Zhao L, Larsson A and Venge P. The identification of a phospholipase B precursor in human neutrophils. *FEBS J*. 2009;276:175-86.
38. Gijon MA, Riekhof WR, Zarini S, Murphy RC and Voelker DR. Lysophospholipid acyltransferases and arachidonate recycling in human neutrophils. *J Biol Chem*. 2008;283:30235-45.
39. Perez-Chacon G, Astudillo AM, Ruiperez V, Balboa MA and Balsinde J. Signaling role for lysophosphatidylcholine acyltransferase 3 in receptor-regulated arachidonic acid reacylation reactions in human monocytes. *Journal of immunology*. 2010;184:1071-8.
40. Malhotra KT, Malhotra K, Lubin BH and Kuypers FA. Identification and molecular characterization of acyl-CoA synthetase in human erythrocytes and erythroid precursors. *Biochem J*. 1999;344 Pt 1:135-43.
41. Korchak HM, Kane LH, Rossi MW and Corkey BE. Long chain acyl coenzyme A and signaling in neutrophils. An inhibitor of acyl coenzyme A synthetase, triacsin C, inhibits superoxide anion generation and degranulation by human neutrophils. *The Journal of biological chemistry*. 1994;269:30281-7.

42. Kim MS, Pinto SM, Getnet D, Nirujogi RS, Manda SS, Chaerkady R, Madugundu AK, Kelkar DS, Isserlin R, Jain S, et al. A draft map of the human proteome. *Nature*. 2014;509:575-81.
43. Li W, Zhu L, Huang H, He Y, Lv J, Li W, Chen L and He W. Identification of susceptible genes for complex chronic diseases based on disease risk functional SNPs and interaction networks. *J Biomed Inform*. 2017;74:137-144.
44. Gellett AM, Kharel Y, Sunkara M, Morris AJ and Lynch KR. Biosynthesis of alkyl lysophosphatidic acid by diacylglycerol kinases. *Biochemical and biophysical research communications*. 2012;422:758-63.
45. Nanjundan M and Possmayer F. Pulmonary phosphatidic acid phosphatase and lipid phosphate phosphohydrolase. *Am J Physiol Lung Cell Mol Physiol*. 2003;284:L1-23.

Supplementary Table 1

Gene	SNP	Genotype	n	total cholesterol (mM, mean ± SD)	total triglycerides (mM, mean ± SD)	CVD incidence
APOE (controls)	E3	E3E3$	39	6.02 ± 0.96	1.91 ± 1.17	2
APOE	E2	E2E2	21	5.08 ± 0.99***	2.15 ± 0.99	0
APOE	E4	E4E4	37	5.69 ± 0.85	2.08 ± 1.39	4
APOA5	rs662799 (A>G)	GG	14	5.39 ± 0.82*	2.76 ± 1.25*	4
SORT1	rs599839 (A>G)	GG	38	5.45 ± 0.81**	1.79 ± 0.87	5
LDLR	rs6511720 (G>T)	TT	21	5.21 ± 1.12**	2.29 ± 2.16	4
Chr9p21	rs10757274(A>G)	GG	33	5.64 ± 0.80*	1.68 ± 0.80	6

Supplementary Table 2

Name	m/z Q1	Name	m/z Q1
TG(32:0)	600,520	TG(52:3)	874,786
TG(34:0)	628,551	TG(52:4)	872,770
TG(36:0)	656,582	TG(52:5)	870,755
TG(38:0)	684,614	TG(54:0)	908,864
TG(40:0)	712,645	TG(54:1)	906,848
TG(42:0)	740,676	TG(54:2)	904,833
TG(44:0)	768,708	TG(54:3)	902,817
TG(46:0)	796,739	TG(54:4)	900,802
TG(48:0)	824,770	TG(54:5)	898,786
TG(48:1)	822,755	TG(54:6)	896,770
TG(48:2)	820,739	TG(56:0)	936,896
TG(50:0)	852,802	TG(56:1)	934,880
TG(50:1)	850,786	TG(56:2)	932,864
TG(50:2)	848,770	TG(56:3)	930,849
TG(50:3)	846,755	TG(56:4)	928,833
TG(50:4)	844,739	TG(56:5)	926,817
TG(52:0)	880,833	TG(56:6)	924,802
TG(52:1)	878,817	TG (51:1)d5	869,837
TG(52:2)	876,802		

Dwell time: 10 msec., declustering potential: 60 V, entrance potential: 10 V; all triacylglycerol species were analysed as $[M+NH_4]^+$ ions. Accordingly the Q1 m/z values refer to this ion species.

Supplementary Table 3

Name	m/z Q1	m/z Q3
Cholesterol	404,4	369,1
CE(14:0)	614,6	369,1
CE(16:0)	642,6	369,1
CE(16:1)	640,6	369,1
CE(16:2)	638, 6	369,1
CE(18:0)	670,6	369,1
CE(18:1)	668,6	369,1
CE(18:2)	666,6	369,1
CE(18:3)	664,6	369,1
CE(20:3)	692,6	369,1
CE(20:4)	690,6	369,1
CE(20:5)	688,6	369,1
CE(22:6)	714,6	369,1
CE18:1 d7	675,6	376,3

Dwell time: 75 msec., declustering potential: 70 V, entrance potential: 10 V, collision energy: 20 V and collision cell exit potential: 25 V; all cholesterol species were analysed as $[M+NH_4]^+$ ions. Accordingly the Q1 m/z values refer to this ion species.

Supplementary Table 4

Name	m/z Q1	m/z Q3
Lyso PC (16:0)	480.3	255.2
Lyso PC (16:1)	478.3	253.2
Lyso PC (18:0)	508.4	283.3
Lyso PC (18:1)	506.4	281.2
Lyso PC (18:2)	504.3	279.2
Lyso PC (20:1)	534.4	309.3
Lyso PC (20:3)	530.3	305.3
Lyso PC (20:4)	528.3	303.2
Lyso PE (16:0)	452.3	255.2
Lyso PE (16:1)	450.3	253.2
Lyso PE (18:0)	480.3	283.3
Lyso PE (18:1)	478.3	281.2
Lyso PE (20:4)	500.3	303.2
Lyso PE (22:6)	524.3	327.2
Lyso PE (18:2)	476.3	279.2
Lyso PC 18: d7	513.8	288.3
Lyso PE 18:1 d7	485.6	288.3

Dwell time: 100 msec., entrance potential: 10 V, collision energy: 36 V, collision cell exit potential: 6 V. Declustering potential was 165 V and 112 V for lyso PC and lyso PE species, respectively. Lyso PC and Lyso PE species were detected as $[M-15]^-$ and $[M-H]^-$ ions, respectively.

Supplementary Figure 1

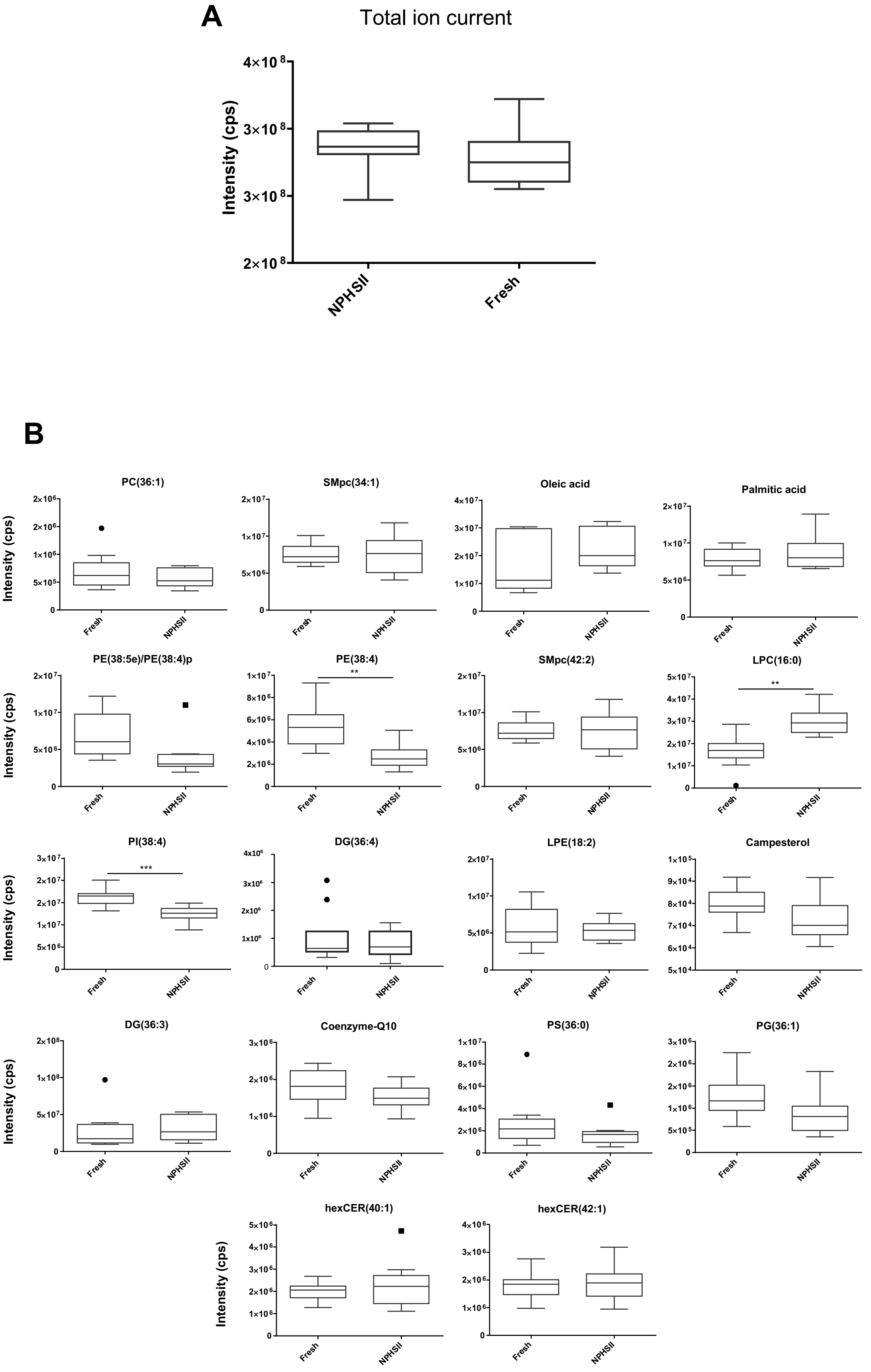

Supplementary Figure 2

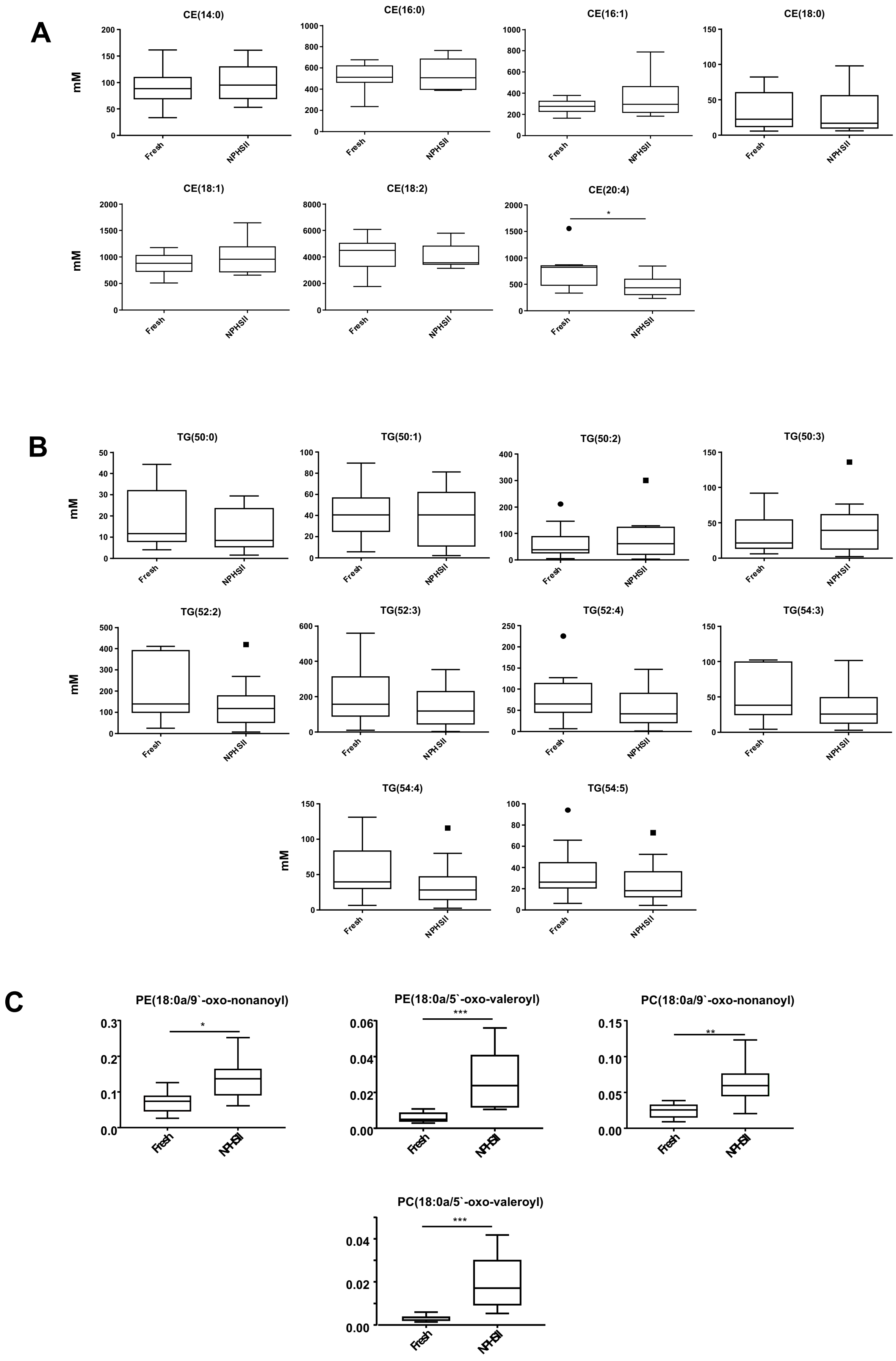

Supplementary Figure 3

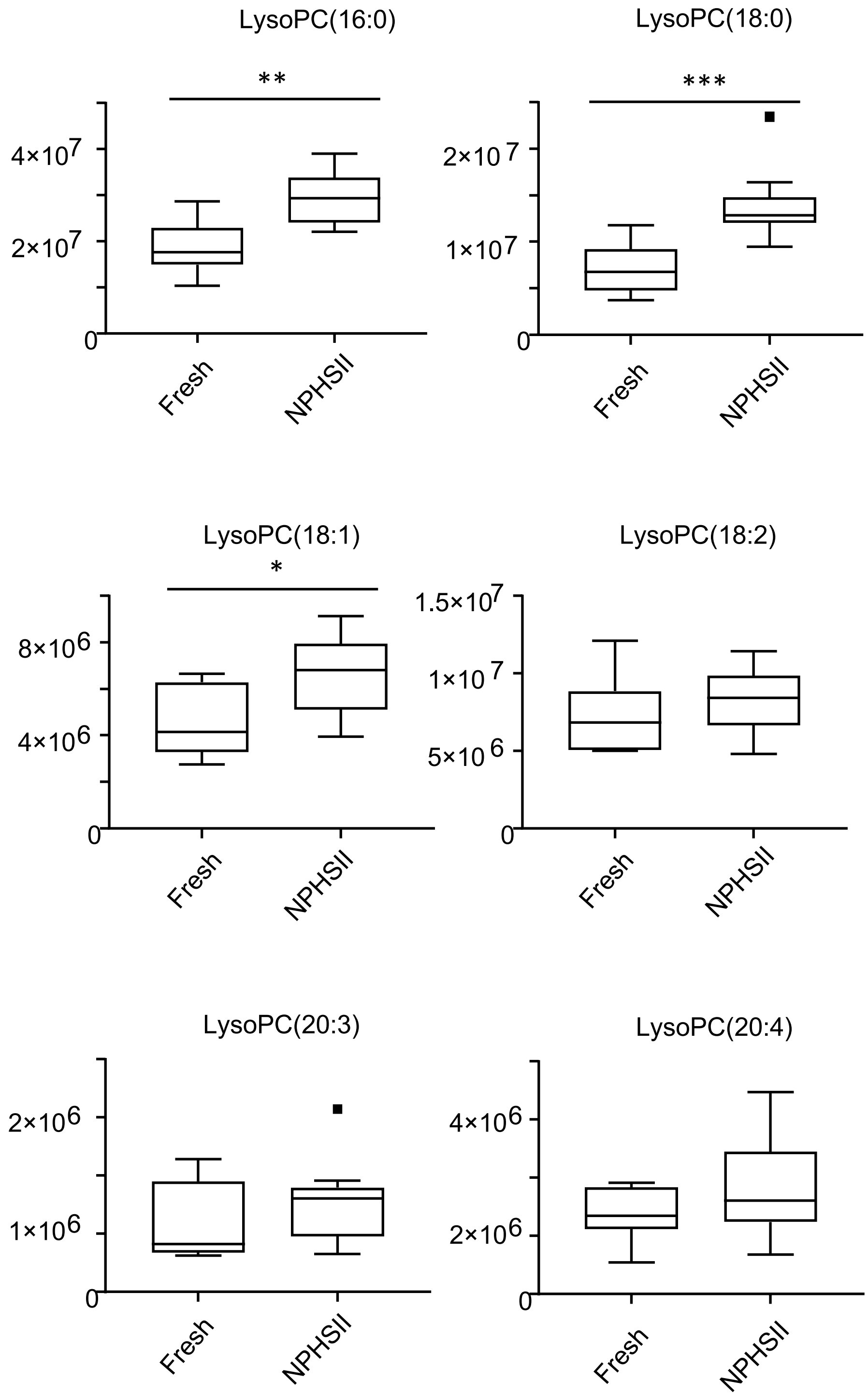

Supplementary Figure 4

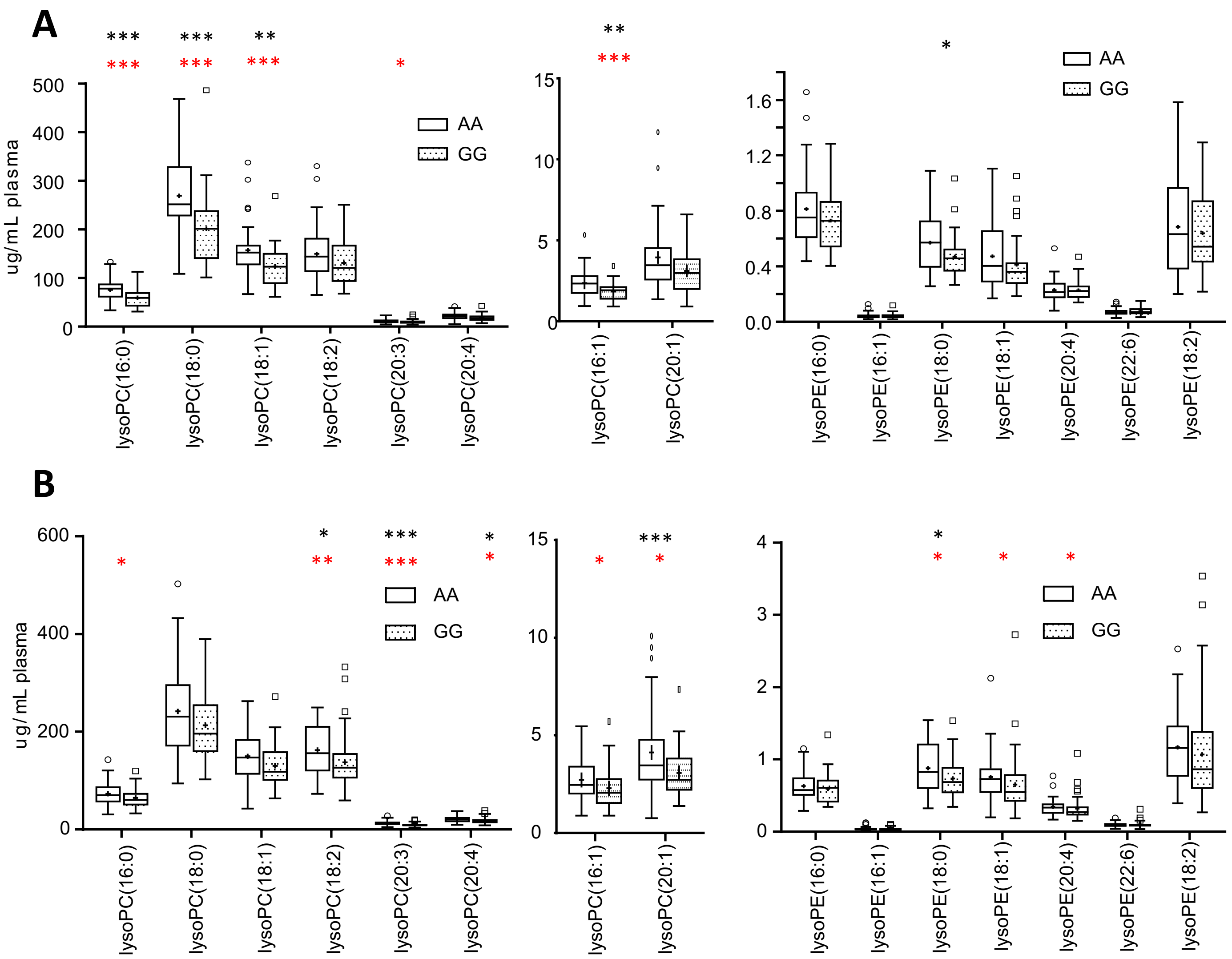

Supplementary Figure 5

48 hr shRNA vs control, Lipid Metabolic Processes

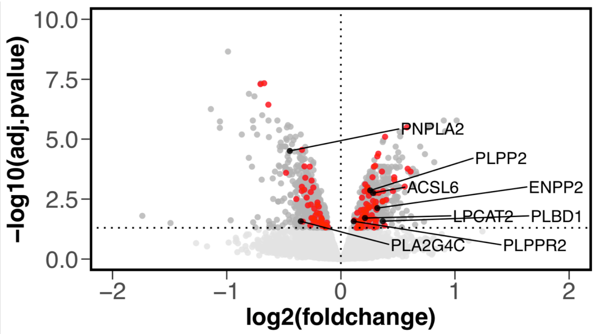

48 hr shRNA vs control, Glycosphingolipid Metabolic Processes

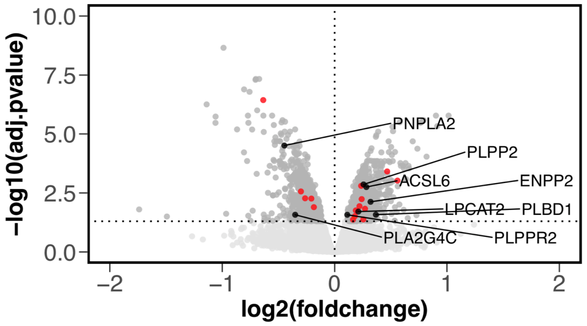

48 hr shRNA vs control, Regulation of Lipid Metabolic Processes

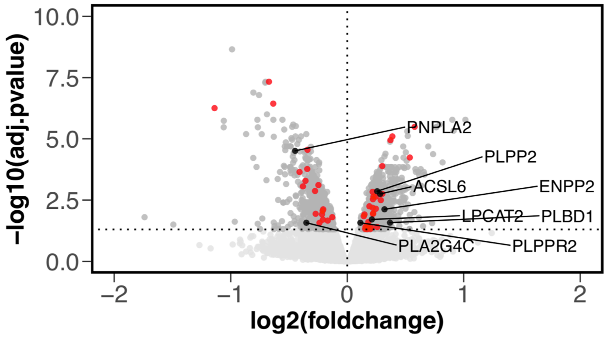

96 hr shRNA vs control, Regulation of Lipid Metabolic Processes

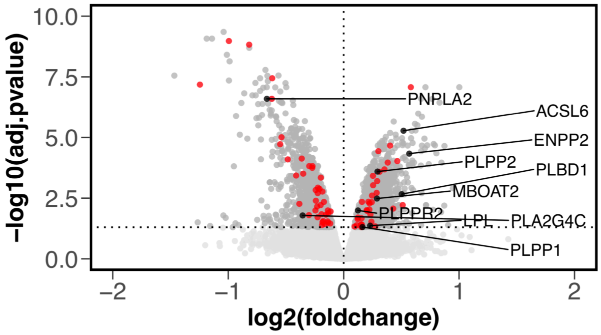

96 hr shRNA vs control, Lipid Metabolic Processes

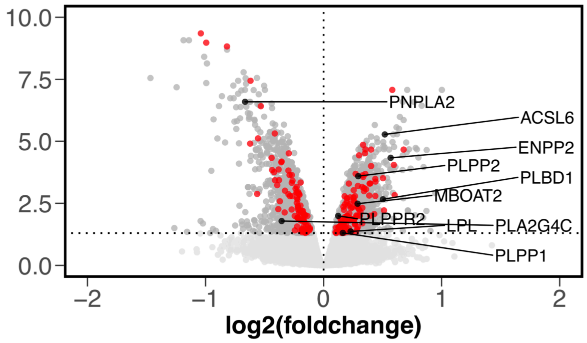

Supplementary Figure 6

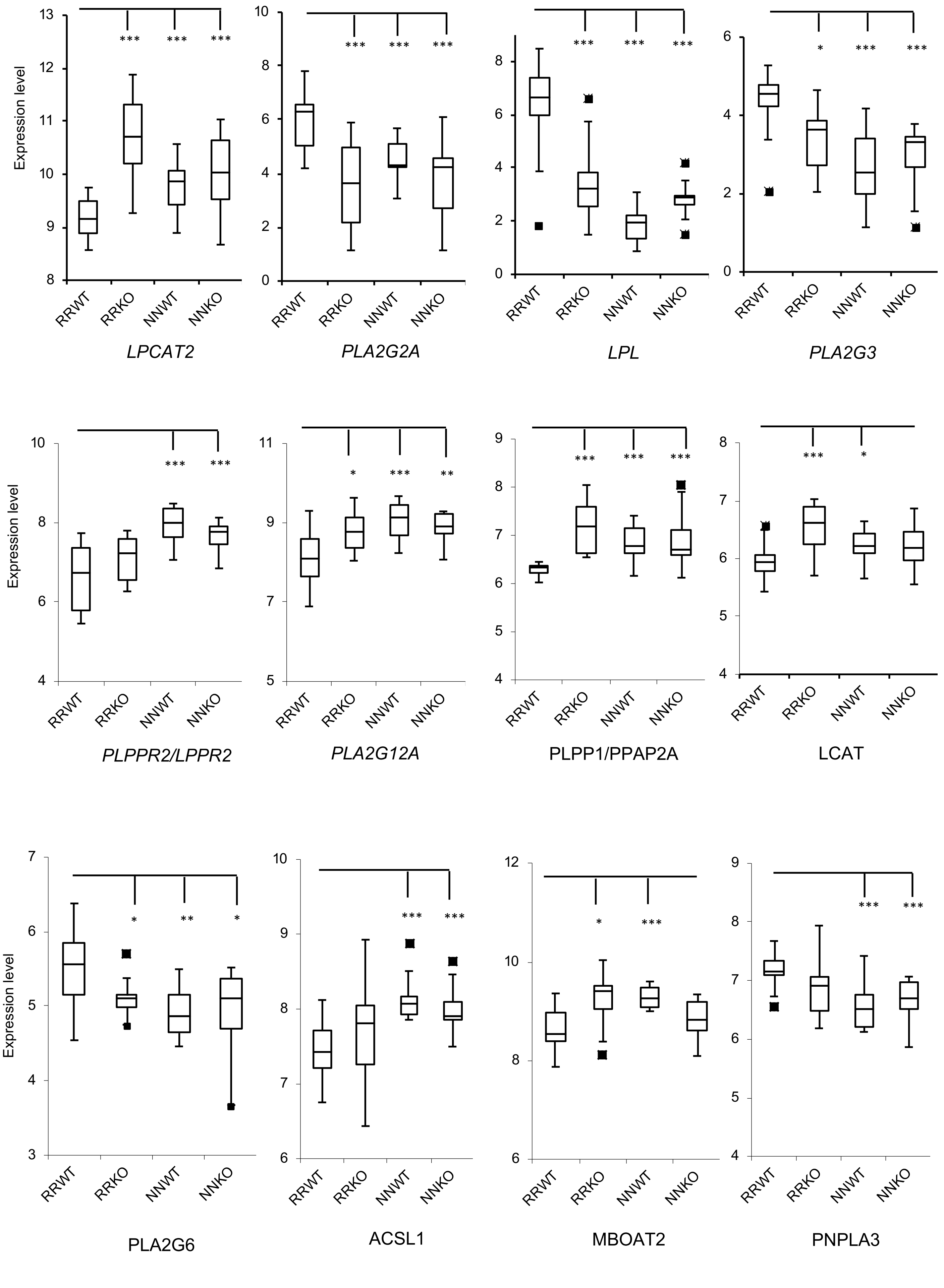

Supplementary Figure 7

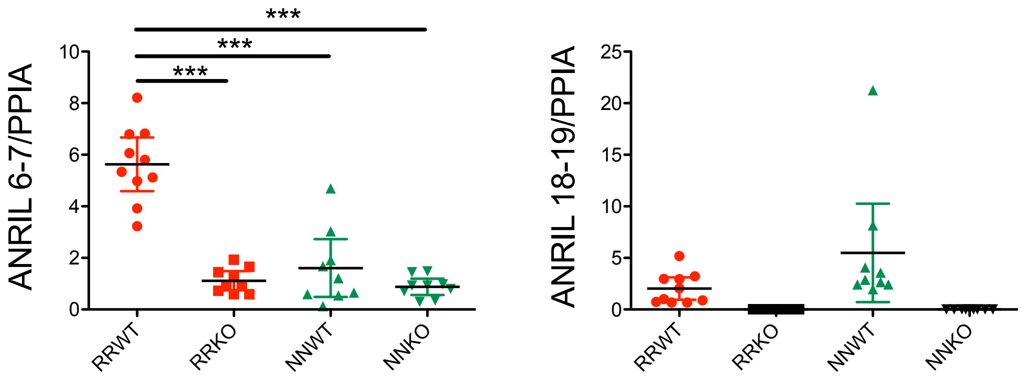

Supplementary Figure 8

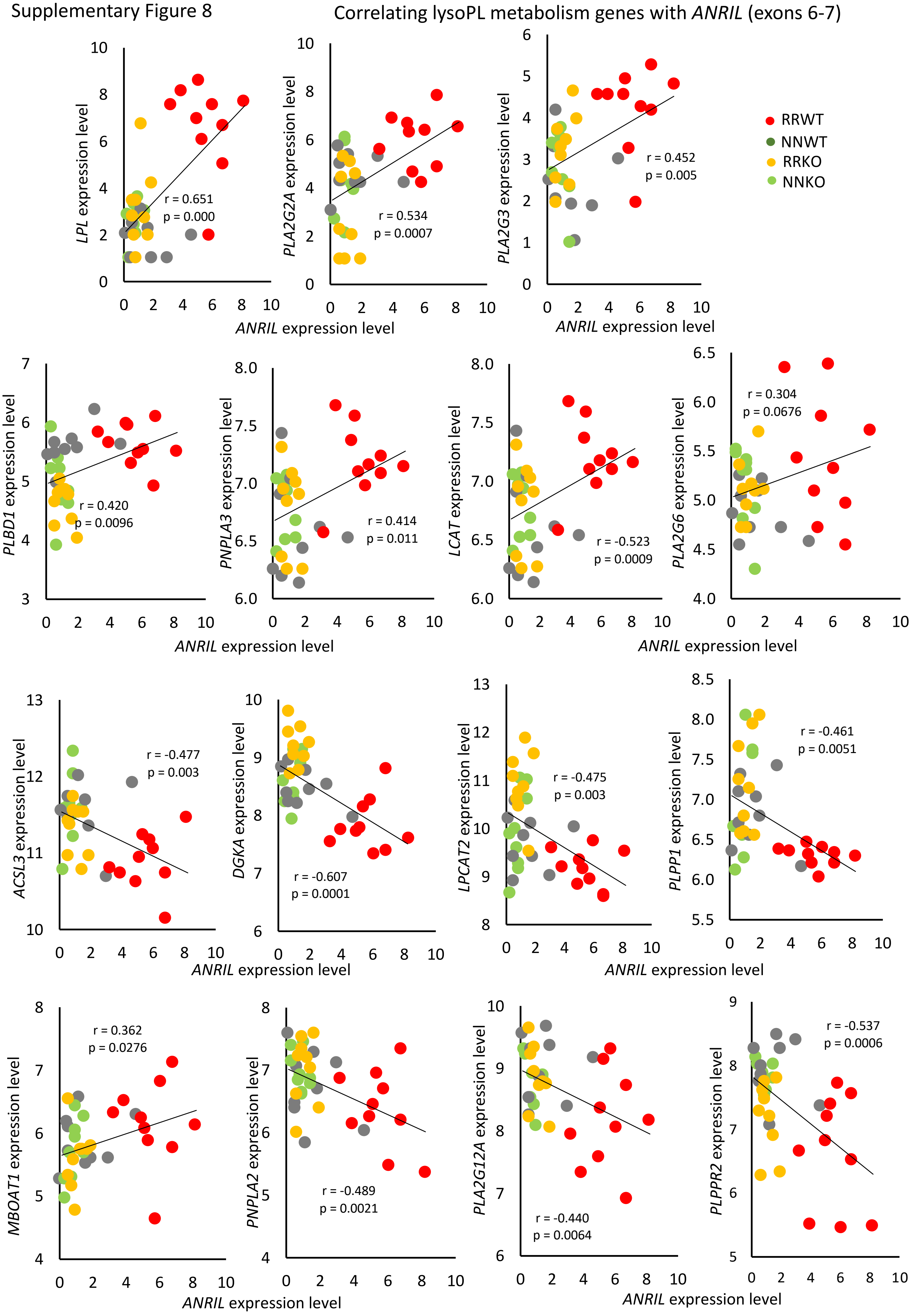

Supplementary Figure 9

Correlating lysoPL metabolism genes with *ANRIL* (exons 18-19)

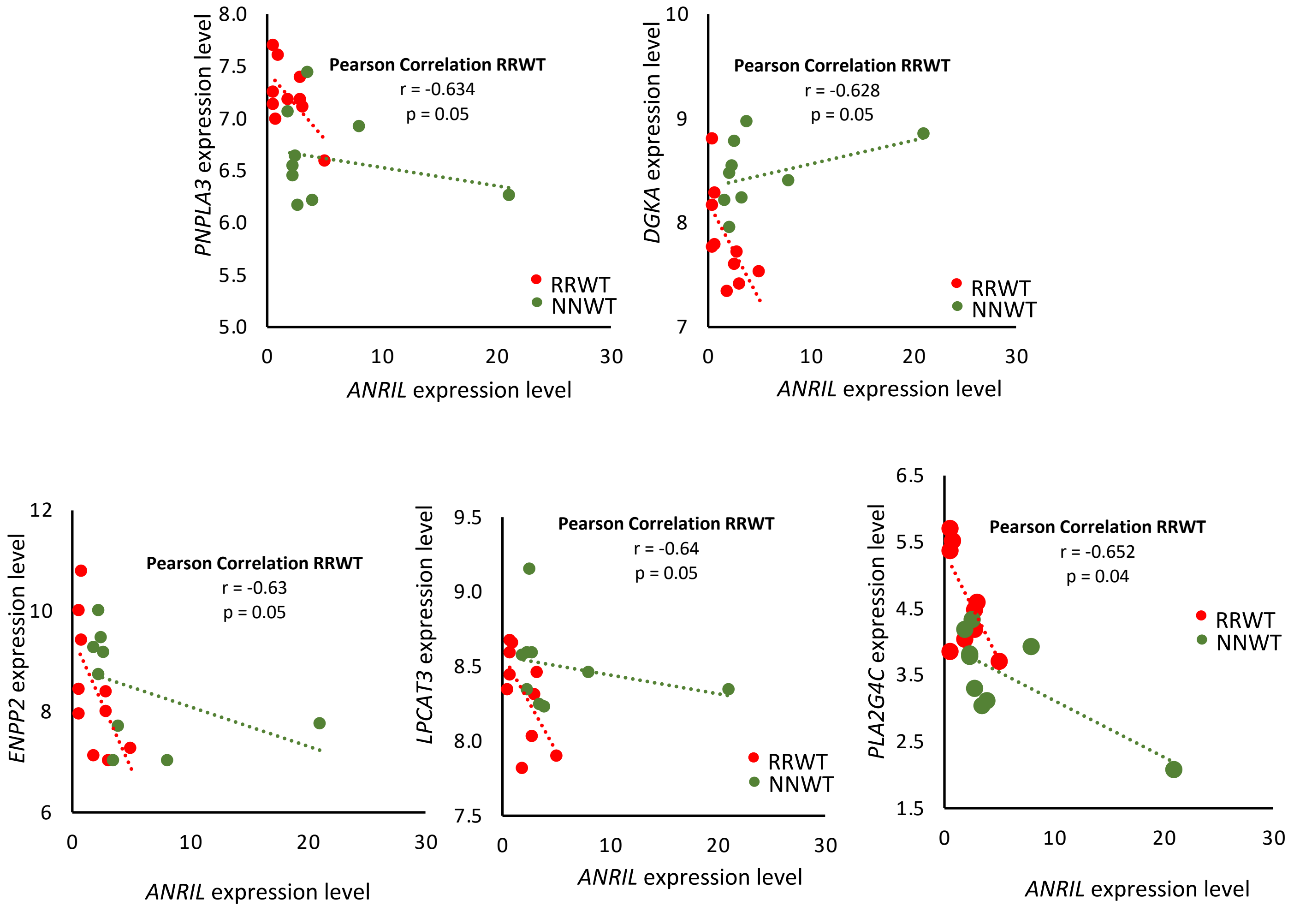

Supplementary Figure 10: Example chromatograms from lysoPL analysis (Cardiff)

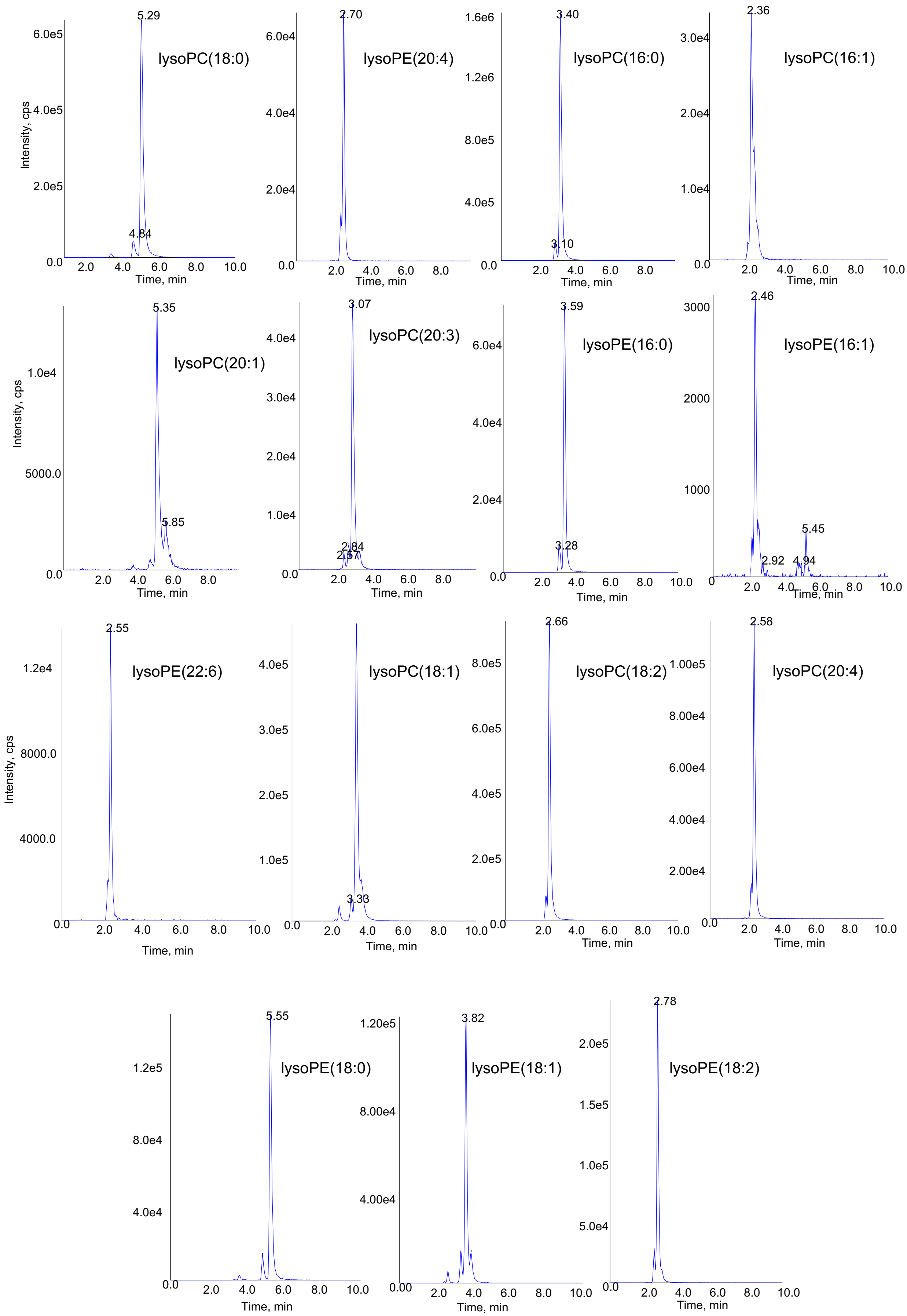

Supplementary Figure 11: Example chromatograms from lysoPA analysis (Tokyo)

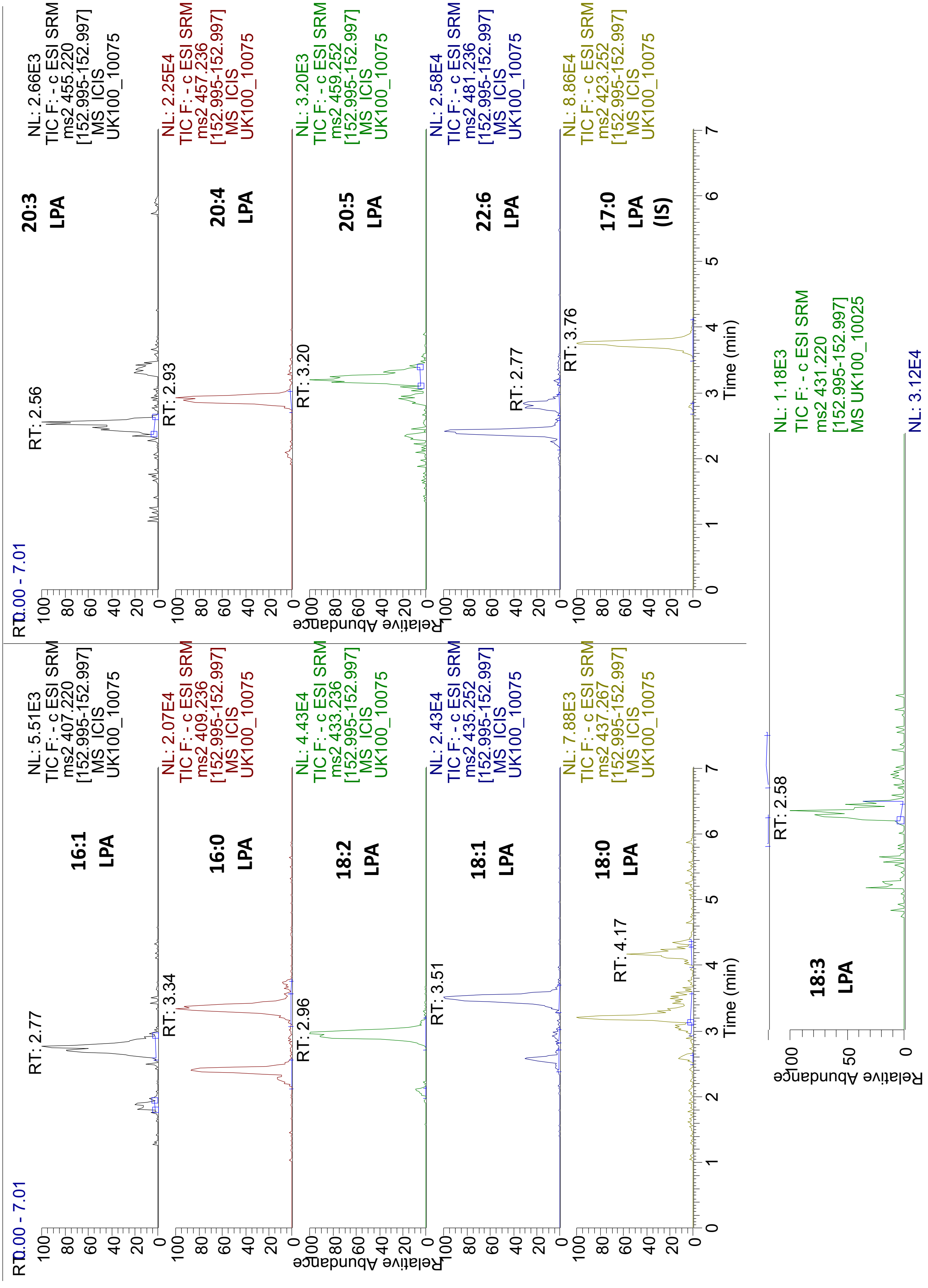

Supplementary Figure 12

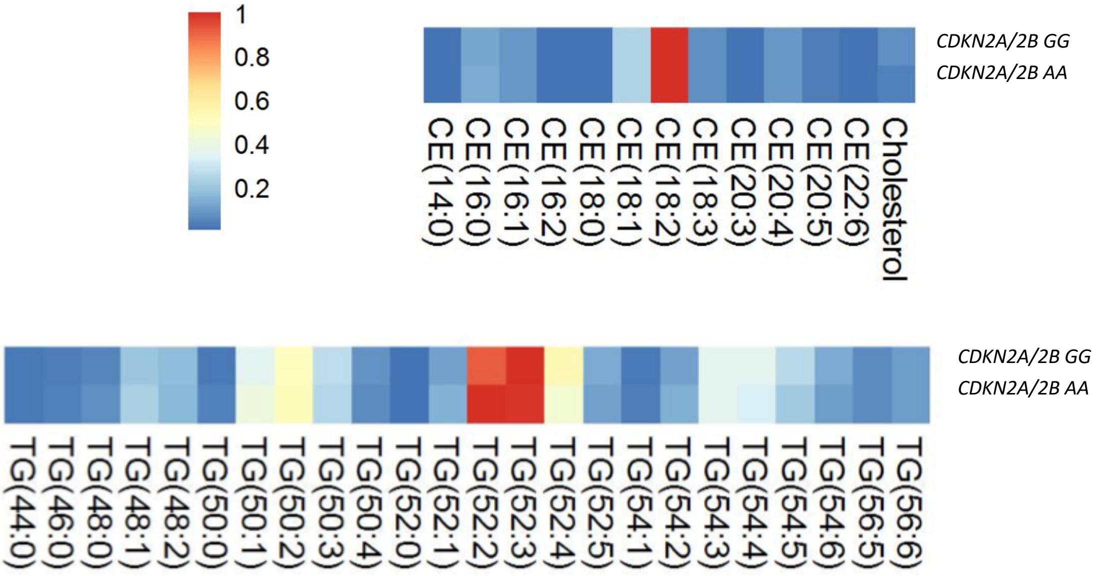

Supplementary Figure 13

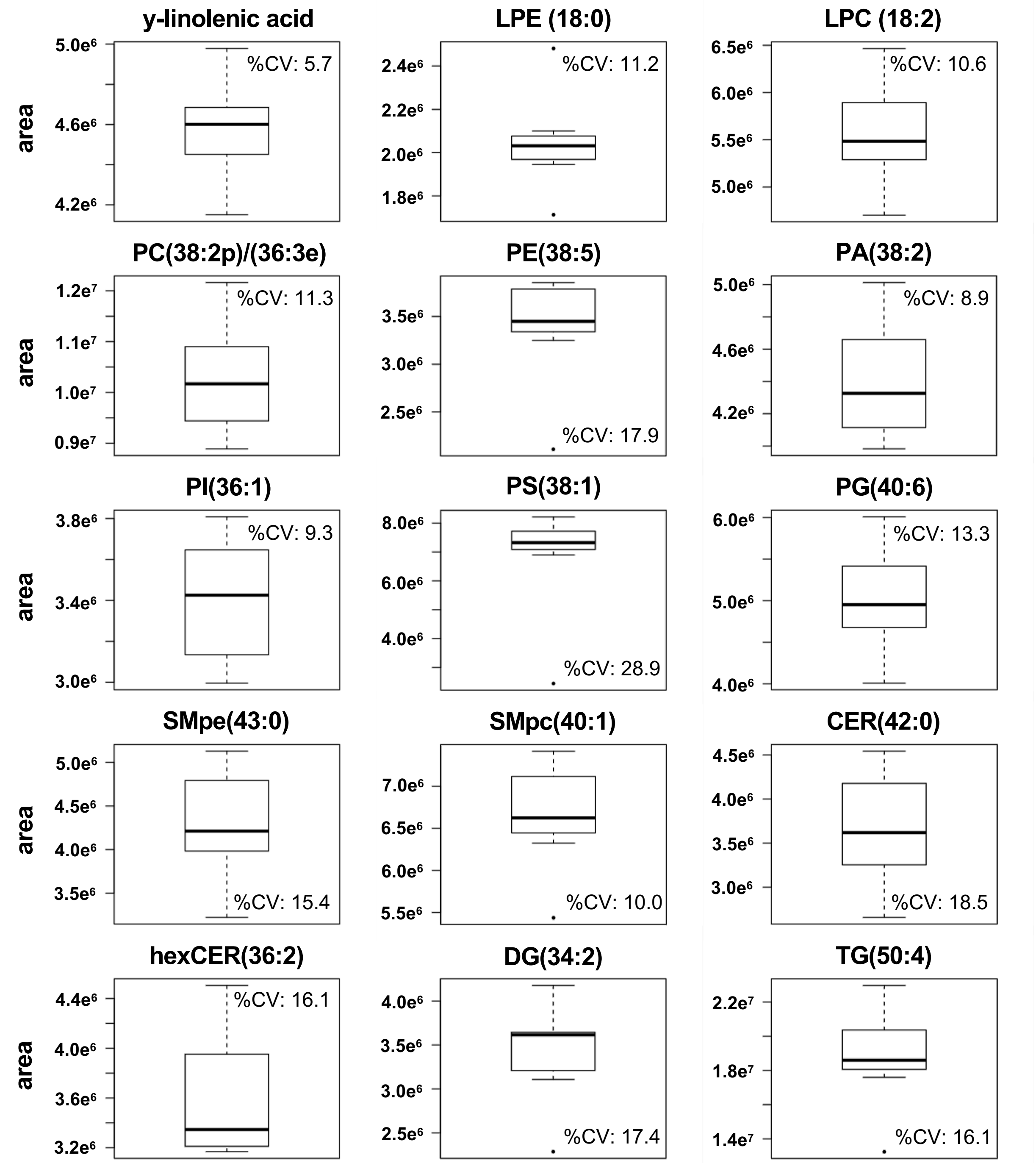

Supplement: Supplementary file 1 [file hcg-13-e002806-s001.pdf]
