# Supplementary material for: Metabolic Dysregulation of the Lysophospholipid/Autotaxin Axis in the Chromosome 9p21 Gene SNP rs10757274
Source: Circ Genom Precis Med. 2020 May 12;13(3):e002806. doi: 10.1161/CIRCGEN.119.002806 (PMC7299226; doi:10.1161/CIRCGEN.119.002806)
Supplement: Supplementary file 3 [file hcg-13-e002806-s003.pdf]

Metabolic Dysregulation of the Lysophospholipid/autotaxin Axis in the Chromosome 9p21 Gene SNP rs10757274

Running title: *Meckelmann & Hawksworth et al., Phospholipids define a cardiovascular risk SNP*

Sven W. Meckelmann, PhD[1,4]*; Jade I. Hawksworth, MSc[1]*; Daniel White, PhD[1]; Robert Andrews, PhD[1]; Patricia Rodrigues, MSc[1]; Anne O`Connor, PhD[1]; Jorge Alvarez-Jarreta, PhD[1]; Victoria J. Tyrrell, PhD[1]; Christine Hinz, PhD[1]; You Zhou, PhD[1]; Julie Williams, PhD[2]; Maceler Aldrovandi, PhD[1]; William J. Watkins, PhD[1]; Adam J. Engler, PhD[3]; Valentina Lo Sardo, PhD[5]; David A. Slatter, PhD[1]; Stuart M. Allen, PhD[6]; Jay Acharya, PhD[7]; Jacquie Mitchell, MSc[7]; Jackie Cooper, MSc[7]; Junken Aoki, PhD[8]; Kuniyuki Kano, PhD[8]; Steve E. Humphries, PhD, MRCP, FRCPath[7]; Valerie B. O'Donnell, PhD[1]

[1]Systems Immunity Research Institute and Division of Infection and Immunity, [2]Division of Neuropsychiatric Genetics and Genomics and Dementia Research Institute at Cardiff, School of Medicine, [6]School of Computer Science & Informatics, Cardiff University, Cardiff, United Kingdom; [3]Dept of Bioengineering, University of San Diego, La Jolla, CA; [4]Applied Analytical Chemistry, Faculty of Chemistry, University of Duisburg-Essen, Essen, Germany; [5]Department of Cellular and Molecular Neuroscience and Dorris Neuroscience Center, The Scripps Research Institute, La Jolla, CA; [7]Cardiovascular Genetics, Institute of Cardiovascular Science, University College London, London, United Kingdom; [8]Tohoku University, Sendai, Miyagi, Japan
*contributed equally

American Heart Association.
Circulation: Genomic and Precision Medicine

Correspondence:
Valerie O'Donnell, PhD
Systems Immunity Research Institute,
Cardiff University, School of Medicine
Heath Park, Cardiff, CF14 4XN
United Kingdom
Tel: 0044 2920 687313
Email: O-donnellvb@cardiff.ac.uk

Journal Subject Terms: Inflammation; Lipids and Cholesterol; Metabolism

Abstract:

Background - Common chromosome 9p21 SNPs increase coronary heart disease (CHD) risk, independent of "traditional lipid risk factors". However, lipids comprise large numbers of structurally related molecules not measured in traditional risk measurements, and many have inflammatory bioactivities. Here we applied lipidomic and genomic approaches to three model systems, to characterize lipid metabolic changes in common Chr9p21 SNPs which confer ~30% elevated CHD risk associated with altered expression of *ANRIL*, a long ncRNA.

Methods - Untargeted and targeted lipidomics was applied to plasma from Northwick Park Heart Study II (NPHSII) homozygotes for AA or GG in rs10757274, followed by correlation and network analysis. To identify candidate genes, transcriptomic data from shRNA downregulation of *ANRIL* in HEK293 cells was mined. Transcriptional data from vascular smooth muscle cells differentiated from iPSCs of individuals with/without Chr9p21 risk, non-risk alleles, and corresponding knockout isogenic lines were next examined. Last, an in-silico analysis of miRNAs was conducted to identify how ANRIL might control lysoPL/lysoPA genes.

Results - Elevated risk GG correlated with reduced lysophosphospholipids (lysoPLs), lysophosphatidic acids (lysoPA) and autotaxin (ATX). Five other risk SNPs did not show this phenotype. LysoPL-lysoPA interconversion was uncoupled from ATX in GG plasma, suggesting metabolic dysregulation. Significantly altered expression of several lysoPL/lysoPA metabolising enzymes was found in HEK cells lacking *ANRIL*. In the VSMC dataset, the presence of risk alleles associated with altered expression of several lysoPL/lysoPA enzymes. Deletion of the risk locus reversed expression of several lysoPL/lysoPA genes to non-risk haplotype levels. Genes that were altered across both cell datasets were *DGKA, MBOAT2, PLPP1* and *LPL*. The in-silico analysis identified four *ANRIL*-regulated miRNAs that control lysoPL genes as miR-186-3p, miR-34a-3p, miR-122-5p, miR-34a-5p.

Conclusions - A Chr9p21 risk SNP associates with complex alterations in immune-bioactive phospholipids and their metabolism. Lipid metabolites and genomic pathways associated with CHD pathogenesis in Chr9p21 and *ANRIL*-associated disease are demonstrated.

Key words: lipids; atherosclerosis; mass spectrometry; phospholipids

Nonstandard Abbreviations and Acronyms

coronary heart disease (CHD)

Northwick Park Heart Study II (NPHSII)

lysophosphospholipids (lysoPLs)

lysophosphatidic acids (lysoPA)

autotaxin (ATX)

cholesteryl esters (CE)

triglycerides (TG)

sequential goodness of fit metatest (SGoF)

risk haplotypes (RRWT)

controls (NNWT)

non-risk (NN)

G protein-coupled receptor (GPCR)

platelet activating factor (PAF)

phospholipase A2 (PLA_2)

Circulation: Genomic and Precision Medicine

Introduction

The association of altered plasma "lipids" with coronary heart disease (CHD) risk has been known for decades, however for some CHD-risk SNPs, there is no association with "traditional lipid measurements", such as lipoproteins (HDL or LDL) or their constituents: cholesteryl esters (CE) and triglycerides (TG) [1]. As a prominent example, the relatively common *CDKN2A/2B* (rs10757274, A>G) (minor allele frequency = 0.48) SNP on chromosome 9p21 confers ~30% elevated risk of CHD, but acts independently of traditional lipid risk factors [1]. Chr9p21 SNPs, including rs10757274, are believed to alter disease risk through modulation of the long non-coding (lnc)RNA, *ANRIL*, although both up and downregulation has been associated with risk (see discussion for more detail) [2, 3]. *ANRIL* isoforms are detected in peripheral blood cells, aortic smooth muscle, endothelial cells and heart, and SNPs in Chr9p21 are associated not only with CHD but also numerous cancers [2, 4-6]. Cellular studies show that *ANRIL* lncRNA down-regulates the tumour suppressors *CDKN2A/2B* by epigenetic regulation, modulating expression of pathways involved in differentiation, apoptosis, matrix remodelling, proliferation, apoptosis, senescence and inflammation [5, 7]. Whether or how the entire CHD risk region or *ANRIL* regulates bioactive lipids is currently unknown.

Lipids represent thousands of diverse molecules. However, CHD clinical risk algorithms such as Framingham or QRISK include circulating lipoproteins only [8, 9]. Importantly, bioactive lipids that regulate vascular inflammation/proliferation in line with the function of *ANRIL* and thus maybe directly relevant to Chr9p21-mediated CHD are not included in these measures. Indeed, whether *ANRIL* mediates its effects via an impact on bioactive lipid signalling has not been examined and was studied herein using lipidomics.

American Heart Association.
Circulation: Genomic and Precision Medicine

Here, plasma from a prospective cohort (Northwick Park Heart Study II, NPHSII) which recruited ~3,000 men aged 50 - 64 years clinically free of CHD in 1990-1991, was analysed using targeted and untargeted lipidomics, followed by validation, metabolic correlation and network analysis [10, 11]. Then, gene transcription for lipid metabolic enzymes was mined in data from a cellular *ANRIL* knockdown study, and from vascular smooth muscle cells differentiated from iPSCs obtained from individuals with/without Chr9p21 risk, non-risk alleles and corresponding isogenic lines deleted of the entire CHD locus [12, 13]. Database mining for potential candidate miRNAs linking ANRIL with gene expression was conducted. The study reveals novel insights into the potential role of key bioactive signalling lipids in this common but poorly understood form of CHD.

American Heart Association.

Methods

Circulation: Genomic and Precision Medicine

The authors declare that all supporting data are available within the article [and its supplementary files]. Ethical approval for use of NPHSII samples was provided by the National Hospital for Neurology and Neurosurgery and the Institute of Neurology Joint Research Ethics Committee, and Joint UCL/UCLH Committee of Human Research, Committees A and Alpha, and all samples were obtained with informed consent. Full methods are provided in Supplementary Materials

Results

Global lipidomics demonstrates that lysoPLs are reduced in GG plasma versus AA

To capture all lipids (knowns/unknowns), high resolution Orbitrap MS data from long chromatographic separations was analysed using XCMS, then processed for cleanup/assignment

to LIPID MAPS categories, using LipidFinder (Figure 1 A,B) [14]. Plasma quality was checked through careful comparison with fresh plasma, detailed in Supplemental Material. Most lipid categories were unchanged, however oxidized phospholipids and lysoPCs had elevated somewhat in storage (Supplementary Figures 1-3). This is not unexpected, and we include a full discussion of this phenomenon in Supplemental Material. To assess the impact of the rs10757274, A>G SNP, we compared AA (n = 39) with the risk genotype GG (n = 33). Data was analysed first using a Mann Whitney U test, then chromatograms for all features with $p<0.075$ were manually checked for quality. LipidFinder detected 1878 lipids, with 872 assigned to a category (Figure 1 B). Next, quantile normalization was applied followed by Mann Whitney U test, and then a p-value adjustment using sequential goodness of fit metatest (SGoF) to each subclass[15]. The SGoF has been shown as especially well-suited to small sample sizes when the number of tests is large. This data is shown in volcano plots in Figure 1 C-J, and the p-values are in column M (Supplementary Data.xls, tabs 1,2). Those most affected by genotype were GPLs and unknowns (Figure 1 C-J, Table 1). Following p-value adjustment the number of significantly different lipids was 17, with 7 putatively identified as lysoPC ions and adducts (supplementary data.xlsx, tab 1,2). An additional group of 8 had p-values close to significance at 0.05-0.08. All were reduced in GG plasma. As this method is used as for hypothesis generation only, we next validated our results using gold-standard quantitative targeted methods.

American Heart Association.
Circulation: Genomic and Precision Medicine

Quantitative targeted lipidomics confirms decreased lysoPLs in the GG samples.

The same plasmas were analysed using a targeted fully-quantitative assay for 15 lysoPLs. Of these several lysoPCs significantly decreased, with both lysoPC and lysoPEs all trending towards lower levels in GG (Supplementary Figure 4 A). This was replicated using new samples from NPHSII (n = 47: AA, 49: GG) (Supplementary Figure 4 B). When both datasets were combined

(n = 82 – 86/group), all 8 lysoPCs were significantly lower in the GG genotype (Figure 2 A). Thus, lysoPLs are overall suppressed in the GG genotype, with a more robust effect on lysoPCs than lysoPEs.

Significantly altered lysoPLs are not detected in five additional CHD risk-altering SNPs.

LipidFinder data was analysed for additional SNPs from the NPHSII cohort, comparing subjects homozygous for the common alleles with subjects homozygous for rare protective alleles for *SORT1*, *LDLR* or *APOE* E2/E2, or rare risk alleles *APOA5* or *APOE4/E4* (Supplementary Table 1). For most lysoPLs, levels were not significantly altered, with the exception of one for *APOA5* (upregulated, lysoPE(18:1), and one for *LDLR* (downregulated, lysoPC(18:2)) (Figure 2 B). This indicates that lysoPLs are consistently reduced only in the GG risk SNP rs10757274.

American Heart Association.

The plasma lysophosphatidic acids(lysoPA)/autotaxin (ATX) axis is dysregulated in the GG group.

Circulation: Genomic and Precision Medicine

Next, lysoPL-related metabolites/enzymes were measured. Metabolism of lysoPL to lysoPA in healthy plasma can be mediated by ATX[16]. Here, we used a targeted LC/MS/MS assay for lysoPAs, and an immunoenzymatic assay for ATX. ATX was significantly decreased (p = 0.026). Based on power calculations (Supplemental Material), an additional set of plasmas was included to increase sample numbers to 95-100 per group for lysoPAs. LC/MS/MS demonstrated overall small reductions, but with several being significantly lower (Figure 2 C,D). Taken with the lysoPL data, this indicates a global suppression of lysoPL/lysoPA/ATX metabolic pathway in the GG group.

Next, correlation analysis was undertaken to examine the contribution of ATX in metabolizing lysoPL to lysoPA. In AA plasmas, ATX showed very weak positive or negative correlations with total lysoPL or lysoPA, respectively (Figure 3 A,B). This agrees with reports

that ATX contributes to lysoPL conversion to lysoPA in healthy subjects [16]. In contrast, in GG plasmas, these weak trends were somewhat reversed (Figure 3 C,D). To look in more depth, we correlated substrates with products (Figure 3 E-H). In the AA group, significant positive correlations were seen for total lysoPA with lysoPL (p = 0.034). Comparing lipids with the same fatty acyl, significant correlation was seen between lysoPA(18:2) and lysoPL(18:2) (p = 0.023) (Figure 3 E,F). This indicates that as the pool of lysoPL increases, the level of lysoPA increases in parallel, and this would be consistent with conversion by ATX. This relationship was fully reversed in the GG group, where total lysoPL, lysoPL(18:2) or lysoPL(20:4) were negatively correlated with their corresponding lysoPAs (p = 0.019, 0.054, 0.019 respectively) (Figure 3 G-I). We next analysed correlation slopes for AA *versus* GG, comparing either lysoPL:lysoPA (Figure 3 E *versus* G), or lysoPL(18:2):lysoPA(18:2) (Figure 3 F *versus* H). Both these comparisons revealed significant differences (p = 0.0264 and 0.0029 respectively) [17]. These data confirm altered metabolism of lysoPL and lysoPA lipids between genotypes. Specifically, conversion of lysoPL to lysoPA appears to be suppressed in the GG homozygotes.

American Heart Association.
Circulation: Genomic and Precision Medicine

The direct contribution of ATX to metabolizing lysoPL to lysoPA was next examined by correlating normalized ratios of lysoPC(18:2):lysoPA(18:2) with ATX. In this comparison, we expect that as ATX increases, the ratio of substrate:product would reduce due to their interconversion. For AA plasma, a weak negative correlation was seen (Figure 3 J). In contrast, a significant positive correlation was observed for GG plasma (Figure 3 K). Thus, as ATX increases, a higher ratio of substrate:product was seen in GG, suggesting a decoupling of ATX from metabolizing lysoPL to lysoPA. Comparing the slopes for AA *versus* GG revealed significant differences based on genotype (p = 0.0157). This further underscores the dysregulation of the lysoPL metabolic pathway in the GG group, and suggests that non-ATX

pathways may mediate lysoPL to lysoPA conversion. Last, the relative ratios of all lysoPL and lysoPA molecular species were unchanged in the GG versus AA groups (Figure 3 L). Thus, while metabolism of lysoPL/lysoPA by ATX is altered, there was no influence of genotype on molecular composition overall. Notably, ATX preferentially metabolises unsaturated lysoPCs[18]. Overall, despite the correlations only showing associations, when taken with our observations that plasma from GG subjects has significantly less ATX protein, and that all lysoPC molecular species are similarly affected, our data strongly evidence that there is less involvement of ATX in metabolising these lipids in GG plasmas.

Next, a Pearson correlation analysis looking at relationships between individual lipids and ATX was next undertaken using Cytoscape. For thresholds, the classification system of Schober was used [19]. Here, we see that there are moderate (r = 0.40-0.69, green) or strong (r = 0.70-1.00 grey) correlations between lipids of the same class, while there are weak (r = 0.10-0.39, red) correlations between different lipid classes (Figure 4 A). Importantly, the key difference in the dataset is that the weak correlations between classes are positive for the AA group, while they are negative for the GG group (Figure 4 A). Overall, this indicates that these lipids behave similarly within AA subjects. In contrast, in GG plasma, while lipid classes still positively correlate within their groups (e.g. lysoPCs correlate strongly with each other), the links between lysoPL and lysoPA are lost. Instead correlations were weakly negative between lysoPE and lysoPA (Figure 4 A). As in Figure 4, ATX weakly positively correlates with lysoPA in the AA group, but instead with lysoPL in the GG group. This analysis reinforces our findings of altered metabolism for lysoPL/lysoPA, but here at the level of individual lipid species.

American Heart Association.
Circulation: Genomic and Precision Medicine

ANRIL knockdown significantly alters lipid and lysoPL metabolism gene expression

Chr9p21 risk SNPs are believed to act via altering expression of *ANRIL*, which regulates cell proliferation/senescence *in vitro* [2, 4, 5]. To examine for a functional link with lysoPL/lysoPA metabolism, we analysed the effect of shRNA downregulation of the proximal *ANRIL* transcripts EU741058 and DQ485454 in HEK 293 cells at 48 hrs and 96 hrs [12]. A GO analysis found significant alterations of several lipid pathways by *ANRIL*, including *Regulation of Lipid Metabolic Processes* (GO: 0019216), *Phospholipid Metabolic Processes* (GO:0006644), *Cellular Lipid Metabolic Process* (GO:0044255) and *Lipid Biosynthetic Processes* (GO:0008610), for example, *Regulation of Lipid Metabolic Processes* was 1.9 or 1.88 fold-enriched (FDR < 0.05, Benjamini-Hochberg) respectively at 48 and 96-hrs respectively (Table 2, Supplementary Data.xlsx, tabs 3,4). Thus, large numbers of lipid-associated genes were significantly differentially regulated (Supplementary Data.xls, tabs 5,6).

American Heart Association.
Circulation: Genomic and Precision Medicine

We next examined the effect of *ANRIL* knockdown on 49 candidate lysoPL metabolism genes (Supplementary Data.xlsx, tab 7). Of these, 9 were significantly changed at both timepoints, and another 6 at a single timepoint (Table 3). Several were consistent with lowered lysoPL/lysoPA including reduced *PNPLA2, PLA2G4C*, increased *LPCAT2, MBOAT2, ACSL6, PLBD1, PLPP1, PLPP2* and *PLPPR2* (Table 3, Figure 4 B,C). Additional relevant genes were regulated, but in the opposing direction, including decreased *LPCAT1* and *LPCAT3* and increased *LPL, PLA2G7,* and *DGKA* (Table 3). *ENPP2* (the gene encoding ATX) was significantly increased by *ANRIL* suppression (Table 3, Figure 4 B). This data is displayed in volcano plots of the full Affymetrix dataset (Figure 4 B,C, Supplementary Figure 5). Genes in red represent significantly different lysoPL metabolizing genes from the lipid GO pathways.

VSMCs generated from iPSCs from Chr9p21 risk haplotypes show altered expression of lysoPL metabolism genes and correlation of expression with ANRIL isoform expression. VSMCs generated by differentiation of iPSCs from humans homozygous for risk haplotypes in Chr9p21 show globally altered transcriptional networks, dysregulated adhesion, contraction and proliferation, with deletion of the risk haplotype rescuing the phenotype [13]. Here, we interrogated an RNAseq dataset of mature iPSC-derived VSMCs for expression of the 49 lysoPL metabolism genes (Supplementary Data.xls, tab 8). Examination of individual genes revealed 14 that were significantly different between RRWT and other lines, and where removal of the risk locus in RR led to partial or complete rescue: *ACSL3, DGKA, PLA2G2A, LPCAT2, LPL, PLA2G3, PLPPR2/LPPR2, PLA2G12A, PLPP1/PPAP2A, LCAT, PLA2G6, ACSL1, MBOAT2, PNPLA3* (Figure 5 A, Supplementary Figure 6). Of these, *DGKA, PLA2G12A and LCAT* were regulated in line with reduced lysoPC/lysoPA.

Circulation: Genomic and Precision Medicine

Multivariate analysis using PCA for expression of these 14 genes shows clear separation of VSMC lines containing the risk haplotypes (RRWT) from controls (NNWT) in PC1 (Figure 5 B). When the risk locus was deleted, the resulting RRKO cell lines instead clustered closer to NNWT and NNKO in PC1 (Figure 5 B). This analysis indicates that expression of several lysoPL metabolizing genes is different in risk haplotype cells, but reverts closer to non-risk (NN) on removal of the 9p21 locus.

Next, correlations of genes that metabolise lysoPLs with *ANRIL* isoforms (exons 6-7 and 18-19) were performed. Deletion of the Chr9p21 locus in the KO VSMC lines starts around exon 9 and runs downstream to the end of the CAD region[13]. Analysis of ANRIL was performed by qPCR detection of ANRIL isoforms containing exons 6-7 (present in long and short isoforms) and exons 18-19 (in long isoforms only). The analysis showed a significant increase of isoforms

containing exons 6-7 in RRWT cells, compared to NNWT cells (Supplementary Figure 7), as previously described[13]. ANRIL expression was minimal in NNWT, with levels comparable to a residual expression of ANRIL detected in both KO lines, possibly due to transcription of truncated transcripts. ANRIL analysis performed using detection of exons 18-19 showed no significant differences between RRWT and NNWT cells. No transcript expression was detected in KO lines as expected, since the deletion encompasses the last 10 exons of the ANRIL gene. These analyses confirmed that ANRIL short isoforms containing exons 6-7 but not 18-19 are upregulated in RRWT VSMCs. To evaluate possible correlations between LysoPLs-related genes and ANRIL expression, all samples were used for *ANRIL* (exons 6-7) analysis, while for correlations with *ANRIL* (exons 18-19), only WT (RR and NN) were tested. Circular *ANRIL* isoforms have not been detected in these cells.

American Heart Association.
Circulation: Genomic and Precision Medicine

(i) ANRIL (exons 6-7)

Several genes correlated significantly, either in a positive or a negative direction with these *ANRIL* isoforms (Supplementary Figure 8). RRWT samples (in red) clustered together as groups, separated from all other samples which were seen to express PL metabolism genes similarly. This was somewhat expected since these PL-metabolism genes were differently expressed in RRWT versus RRKO, NNWT, NNKO, as shown for ANRIL (6-7) expression (Figure 5 B, Supplementary Figure 7). However, the significant Pearson correlations between *ANRIL* (exons 6-7), and the individual genes show a direct association between this form of these *ANRIL* isoforms and some lysoPL genes.

(ii) ANRIL (exons 18-19)

Here, correlations were tested using RRWT or NNWT clones separately, and then compared. Five genes were identified where a significant negative correlation between *ANRIL* (18-19) and

lysoPL gene expression was seen (*PNPLA3, DGKA, ENPP2, LPCAT3, PLA2G4C*) in the RR clones. In contrast, correlations in NN samples were weaker, and not significant (Supplementary Figure 9). This suggests an impact of *ANRIL* (18-19) isoforms on gene expression, that is absent/reduced in NN. One NN clone displayed higher levels of *ANRIL* (18-19) compared to others, and as an outlier had a large impact on the correlation, reducing statistical power.

In silico analysis of miRNA databases suggests potential candidates for ANRIL regulation of lysoPL gene expression.

ANRIL displays sponge activity towards miRNAs[20]. To examine whether this could mechanistically link *ANRIL* with lysoPL gene expression, we undertook an in-silico analysis using two databases (including one that is experimentally validated: TarBase v8 (http://carolina.imis.athena-innovation.gr/diana_tools/web/index.php?r=tarbasev8%2Findex) and TargetScan (*http://www.targetscan.org/vert_72/). We searched whether miRNAs known to be inhibited by *ANRIL* interact with PL-metabolising genes that are altered in HEK or VSMC datasets. Here, the expected outcome is that target genes should be regulated in the same direction as *ANRIL*. Some hits were found, including two that were conserved across both datasets. In the HEK data, the miRNAs that interact with down-regulated genes were miR-186-3p, miR-34a-3p (*LPCAT1*) and miR-122-5p, miR-34a-5p (*LPCAT3*). In the VSMC dataset, where *ANRIL* (exons 6-7) is significantly upregulated in RR, we focused on genes that were elevated in RR and reduced when the locus was deleted. Here, we found miR-34a-5p (*PLA2G6*) and miR-122-5p (*PNPLA3*). These hits were all from the experimentally validated database (Tarbase) and differences in the target genes impacted may be due to the different cell types used.

American Heart Association.
Circulation: Genomic and Precision Medicine

Discussion

Lipidomics MS is increasingly applied to prospective CHD cohorts that contain no genetic information, while conversely GWAS studies have examined associations with traditional "lipid" measures only (e.g. total cholesterol or triglycerides) [21-35]. Cohorts are only now starting to examine the association of individual lipid molecular species with specific risk SNPs, and little information on this is yet available. Cohort lipidomics is an area that is increasing in popularity, however there are some serious pitfalls with using only untargeted methods. Including a high degree of validation, we show that a common Chr9p21 (rs10757274, A>G) CHD-risk SNP is associated with metabolic alterations to the lysoPL/lysoPA/ATX axis in human plasma (Figures 1-4). This revealed a genotype-specific change that was absent in five other GWAS-proven CHD-risk SNPs. Since the action of rs10757274 GG is independent from "traditional lipid" measurements, it may represent a different component of the disease, characterized in part by changes to bioactive signalling phospholipids (PL), rather than storage/energy lipid pools [1].

LysoPLs have an emerging role in cardiovascular disease that is not yet understood. *In vitro*, they mediate G protein-coupled receptor (GPCR) signalling that causes immune cell migration and apoptosis. This has led them to be proposed as "pro-inflammatory" [36-40]. However, this is disputed since most lysoPL is bound to albumin, immunoglobulins and other plasma carrier systems, and levels are already higher than required for mediating GPCR activation [41-43]. Importantly, recent cohort studies have shown that plasma lysoPC is inversely related to incidence of an event. These include Malmö, Bruneck, TwinGene, ULSAM and PIVUS, which showed correlations of lower lysoPC with incident CHD risk, using untargeted lipidomics [31, 44, 45]. Also, patients on haemodialysis show higher risk of a CHD event and elevated mortality with lower lysoPC[43]. Lower lysoPCs are also associated with CHD factors

American Heart Association.
Circulation: Genomic and Precision Medicine

such as visceral obesity, although since lysoPA cannot be measured using shotgun or untargeted methods, we have not found other cohort data that includes this lipid as yet [27, 31, 32]. In the Bruneck cohort, inclusion of lysoPCs in classifiers improved power for CHD risk prediction, indicating that although the reduction is rather modest, it is clinically significant[31]. Furthermore, the Malmö cohort reported that CHD development is preceded by reduced levels of lysoPCs, around 8 % similar to our data [44]. In Malmö and Bruneck, the lipidomics was limited to untargeted and shotgun methods without further validation, thus our new data provides stronger analytical confidence while linking their findings to a specific risk locus. Given the prevalence of rs10757274 GG in the general population (~23%), our data may at least in part explain the findings in other cohorts with lower lysoPLs now associating with a sub-group with a common SNP. In contrast, it is also known that elevations in long chain unsaturated lysoPA maybe a feature of an acute cardiac event, where a sudden plaque rupture results in generation/release, likely via activation of platelet phospholipases[18].

American Heart Association.
Circulation: Genomic and Precision Medicine

In addition to lipid class-specific changes in phospholipids, many significantly decreased "unknowns" were found, which are currently absent in databases (Figure 1). The plasma lipidome contains large numbers of such species and a significant challenge lies in their structural and biological characterization. The comprehensive list of all lipids detected with fold-change and significance levels is provided (Supplementary data.xlsx, tab 1) as a resource for further mining.

We next searched for potential mechanisms to explain the lipid changes using datasets from two cell models of *ANRIL* modulation, since there is increasing evidence that this long non-coding RNA plays a central role in Chr9p21-linked CVD. *ANRIL* is expressed by exons contained within Chr9p21, and there are many isoforms including long, short and circular,

resulting from alternative splicing across several exons. Both increases and decreases of various *ANRIL* transcripts have been reported to be associated with CVD. For example, compared to AA individuals, GG and several other Chr9p12 risk SNPs have almost 50% lower *ANRIL* (exon 2) in peripheral blood cells[2]. This agrees with the finding that multiple risk alleles are associated with a decrease in *ANRIL* (exons 1-2) in PBMCs[5]. However, others showed various *ANRIL* transcripts are increased in carriers of risk alleles, including from exons 1-5, 7-13 and 18-19, with no change at exons 7b or 10-13b[3]. Elsewhere, expression of short variants (exons 1-2, ending with alternative 13, and exons 1,5-7+13) were increased while long variants (coded by exons 1-12+14-20) were decreased in risk allele carriers[6]. In the VSMC dataset used in our study, lines carrying risk haplotypes (which were also GG for rs10757274) expressed higher levels of *ANRIL* transcripts (exon 6-7) compared to NNWT, which have a minimal expression, similar to a residual expression detected in KO lines[13]. In contrast, the HEK inducible knockdown targeted proximal alternatively spliced *ANRIL* transcripts EU741058 (exons 1,5-7,13) and DQ485454 (exons 1-12 and alternative exon 13). Thus, while they both model human CVD they differ significantly in terms of their impact on *ANRIL*.

American Heart Association.
Circulation: Genomic and Precision Medicine

While it is known that *ANRIL* gene products regulate metabolic genes in cultured cells and stimulate VSMC proliferation while reducing adhesion and contraction, the impact of *ANRIL* on lysoPL/lysoPA metabolism is not characterised [12, 13]. Here, we showed in both HEK and VSMC datasets that there was a significant impact on a large number of lysoPL/lysoPA metabolism genes, with GO term analysis identifying a large number of lipid terms being significantly altered in HEK cells. In the case of VSMCs, removal of the risk locus indicated that several affected genes were directly influenced by the Chr9p21 locus (Figure 5 A,B). Our in-silico screen also identified four miRNAs across the cell types with two candidates identified

from both HEK and VSMC lysoPL gene regulation: miR-34a-5p (*LPCAT3, PLA2G6*), miR-122-5p (*LPCAT3*, *PNPLA3*). Collectively, this suggests that Chr9p21 risk alleles may alter lysoPL/lysoPA in humans via *ANRIL* regulation, providing novel insights into the biology of this important cause of CHD.

Many of the candidate genes are expressed in leukocytes, platelets, erythrocytes, heart, adipose tissue and plasma, thus measuring them in plasma is not possible. However, we could measure ATX (*ENPP2*), a plasma enzyme that converts primarily unsaturated lysoPC to lysoPA in healthy subjects [18]. ATX protein was reduced and furthermore, correlation analysis was consistent with lower ATX activity (Figure 2 C, 3 A-K), providing a potential explanation for lower lysoPAs in the GG group. In line with this, *ENPP2* expression negatively correlated with *ANRIL* (exons 18-19) in VSMC from the risk group, suggesting an association between this gene and a risk form of *ANRIL* (Supplementary Figure 9). Also, *ENPP2* was elevated in HEK cells which lack *ANRIL* transcripts that contain exons 6,7. Furthermore, it has been reported that inflammatory cytokine induction of *ENPP2* is suppressed by 50% in primary human monocyte derived macrophages that carry the Chr9p21 risk haplotype allele[46]. The contribution of ATX to CHD is not understood and may vary with underlying genetic cause[47]. Indeed, while it metabolizes lysoPL to lysoPA in health, in acute coronary syndromes other pathways appear to predominate [18]. This mirrors our suggestion that ATX might be less involved in plasmas with elevated CVD risk, with other uncharacterised pathways being relevant. Here, significant downregulation of ATX in GG plasma from middle-aged men who are otherwise healthy and without clinically detectable CHD was seen indicating it precedes cardiovascular events in this group (Figure 2 C). While we suggest that reduced lysoPA maybe at least in part relate to reduced levels of plasma ATX, additional candidates were identified through transcriptional

American Heart Association.
Circulation: Genomic and Precision Medicine

analysis including *PLPP1*, *PLPP2*, *PLPPR2* (all induced in HEK cells) or *DGKA* (reduced in RRWT VSMC cell lines).

In healthy subjects, lysoPLs, particularly lysoPC, circulate at relatively high concentrations, where they could be generated by (i) lipases bound to the cell surface of endothelial cells in liver, heart and adipose tissues (*LPL, LIPC, LIPG*), (ii) Land's cycle enzymes in circulating blood cells/platelets [16], (iii) lecithin-cholesterol acyl transferase (*LCAT*) trans-esterification in the liver, or (iv) by remodelling pathways for platelet activating factor (PAF) removal (Figure 6). In healthy tissue, lipases predominate, but during vascular inflammation the balance may alter but this is not well characterised. The Land's cycle involves phospholipase A2 (PLA_2) hydrolysis, although the isoforms controlling blood levels are not fully known. Candidates include stromal isoforms and cellular or secreted PLA_2s from circulating cells and platelets, that may become relevant during inflammation. Also, a role for circulating/platelet PLA_1 from platelets in lysoPL formation has been proposed [40]. The reduction in lysoPCs is consistent with significantly different genes identified in the datasets, for example: *ACSL6, MBOAT2, LPCAT2, PLBD1* (all induced in HEK cells), *PNPLA2* and *PLA2G4C* (downregulated in HEK cells), and *PLA2G12A* and *LCAT* (reduced in RRWT VSMC cell lines). Notably, the transcriptional datasets showed complex and largely different changes in lysoPL gene expression, which may relate to them being from different cell types, and with different manipulations to *ANRIL* isoform expression. However, four genes were significantly altered across both datasets: *MBOAT2 and LPL* (always upregulated) and *DGKA, PLPP1* (increased in HEK, decreased in VSMC). A detailed discussion of the known expression patterns and roles of all these genes is provided in Supplemental Material.

American Heart Association.
Circulation: Genomic and Precision Medicine

Notably, many of the genes found to be altered in the transcriptional data are genes with known SNPs that correlate with traditional lipid measures. For many of these genes, it is not clear how or even if they regulate lipid levels directly or are simply associated with altered levels (in the case of traditional lipids). Cardiovascular disease is complex with different forms, however in the case of Chr9p21 linked CHD, a strong phenotype of coronary artery disease is noted[48]. Similarly, traditional lipid levels are also strongly associated with a strong phenotype of coronary artery disease. This indicates that clinical outcomes are similar despite the different genetic origins and for this to be the case, some convergence in biochemical or cellular pathways downstream of genetics would be expected. A potential explanation for our findings is that downstream of *ANRIL*, some of the same genes that are already known to be genetically associated with CHD are subtly altered at their gene expression levels through transcriptional mechanisms, such as proposed here (e.g. miRNA sponge activities of *ANRIL*), and that this then goes on to impact inflammation. This would promote development of coronary artery disease, through some common mechanisms, independent of the biology of traditional lipoprotein measures. While several genes were found to be altered in either the HEK or VCMC datasets, more research is required to link gene expression changes with lysoPC/lysoPA levels, including protein and enzyme activity measurements from carriers of Chr9p21 risk SNPs. LysoPCs and lysoPAs are metabolised in a complex manner, with many gene products in the vasculature expected to play a contributing role. It is also the case that "standard lipids" are metabolised in a complex manner, and that changes in one relevant gene could be compensated by changes in others, that overall lead to standard lipids such as TGs and CEs being overall unchanged in these subjects.

American Heart Association.
Circulation: Genomic and Precision Medicine

We also note that many genes in the HEK or VSMC analysis changed in directions that on their own could be considered to predict increases in lysoPC/lysoPA. However, overall it is the combination/balance of several enzymatic activities that will determine the flux of lipids through this pathway, and thus, their overall levels at steady state. Thus, changes in some could be over-compensated by changes in others, leading to the observed phenotype of overall reduced levels of these lipids. For further reference, genes with SNPs that have been found to associate with standard lipid traits, and were also found to change herein are listed in[49, 50].

A final question relates to how *ANRIL* and lysoPLs are functionally connected. PL metabolism is finely tuned during cell proliferation, with higher concentrations of lysoPC and lysoPE detected at G2/M, which fall dramatically along with concomitant increases in PC/PE due to acylation during progression to G1 [51]. This provides the PL membranes required to complete the cell cycle. Given *ANRIL's* ability to regulate cell proliferation, and observations that silencing *ANRIL* prevents division and promotes senescence, the lower levels of lysoPLs in plasma may simply reflect altered rates of cell turnover in the vasculature, but this remains to be determined (Figure 5 C,D). Here, four miRNAs known to be regulated by *ANRIL*, that also suppress expression of selected lysoPL genes across both HEK and VSMC datasets were found miR-186-3p, miR-34a-3p, miR-122-5p, miR-34a-5p. These potential hits can be followed up as candidate downstream mediators of *ANRIL's* regulation of lysoPL/lysoPA metabolism. Several have well known roles in regulating proliferation and notably circulating miR-122-5p associates with acute myocardial infarction[52].

In summary, we reveal an association of altered PL metabolism with CHD risk in a common risk SNP. The alterations in multiple lysoPL/lysoPA regulatory pathways seen on *ANRIL* silencing, or the presence/removal of the risk locus *in vitro,* further suggest the

American Heart Association.
Circulation: Genomic and Precision Medicine

involvement of bioactive signaling lipids in this form of vascular disease, and mechanistic studies are warranted. To this end, fresh blood from AA and GG subjects is required to measure plasma and cellular levels of all candidate enzymes and relevant lipids, in order to identify how lysoPC/lysoPA metabolism is altered by the presence of the risk SNP. It would also be important to compare AG with AA and GG subjects (also including women), and also those who go on to have events. However, for this considerably larger sample numbers would be required than we have available currently in NPHSII. Furthermore, if heparin is administered to subjects then *LPL*, *LIPC, LIPG* enzymes would be released and could be measured in plasma.

Author contributions: SM, JH, DW, PR, AE, MA, YZ, AC, CB, JAJ, RA, VT, CH, DS, JAoki, VLS, KK conducted experiments and undertook data analysis. SA supervised computational tool design. JAcharia, JC and JM collected and processed clinical samples. SM, VOD and SH conceived the experiments, designed the studies, and drafted the manuscript. All authors edited and approved the manuscript.

Circulation: Genomic and Precision Medicine

Acknowledgments: We gratefully acknowledge expert discussion with Drs Gerhard Liebisch (Regensburg), and Kristin Baldwin (Scripps Research Institute) during revision of the manuscript.

Sources of Funding: Wellcome Trust (094143/Z/10/Z), British Heart Foundation (RG/12/11/29815) and European Research Council (LipidArrays) to VBO. VBO is a Royal Society Wolfson Research Merit Award Holder and acknowledges funding for LIPID MAPS from Wellcome Trust (203014/Z/16/Z). SEH acknowledges grant RG008/08 from the British Heart Foundation, and the support of the UCLH NIHR BRC.

Disclosures: The authors declare no competing interests.

References:

1. Angelakopoulou A, Shah T, Sofat R, Shah S, Berry DJ, Cooper J, Palmen J, Tzoulaki I, Wong A, Jefferis BJ, et al. Comparative analysis of genome-wide association studies signals for lipids, diabetes, and coronary heart disease: Cardiovascular Biomarker Genetics Collaboration. *Eur Heart J*. 2012;33:393-407.

2. Cunnington MS, Santibanez Koref M, Mayosi BM, Burn J, Keavney B. Chromosome 9p21 SNPs Associated with Multiple Disease Phenotypes Correlate with ANRIL Expression. *PLoS Genet*. 2010;6:e1000899.

3. Holdt LM, Beutner F, Scholz M, Gielen S, Gabel G, Bergert H, Schuler G, Thiery J, Teupser D. ANRIL expression is associated with atherosclerosis risk at chromosome 9p21. *Arterioscler Thromb Vasc Biol*. 2010;30:620-7.

4. Liu Y, Sanoff HK, Cho H, Burd CE, Torrice C, Mohlke KL, Ibrahim JG, Thomas NE, Sharpless NE. INK4/ARF transcript expression is associated with chromosome 9p21 variants linked to atherosclerosis. *PLoS One*. 2009;4:e5027.

5. Congrains A, Kamide K, Oguro R, Yasuda O, Miyata K, Yamamoto E, Kawai T, Kusunoki H, Yamamoto H, Takeya Y, et al. Genetic variants at the 9p21 locus contribute to atherosclerosis through modulation of ANRIL and CDKN2A/B. *Atherosclerosis*. 2012;220:449-55.

6. Jarinova O, Stewart AF, Roberts R, Wells G, Lau P, Naing T, Buerki C, McLean BW, Cook RC, Parker JS, et al. Functional analysis of the chromosome 9p21.3 coronary artery disease risk locus. *Arterioscler Thromb Vasc Biol*. 2009;29:1671-7.

7. Yap KL, Li S, Munoz-Cabello AM, Raguz S, Zeng L, Mujtaba S, Gil J, Walsh MJ, Zhou MM. Molecular interplay of the noncoding RNA ANRIL and methylated histone H3 lysine 27 by polycomb CBX7 in transcriptional silencing of INK4a. *Mol Cell*. 2010;38:662-74.

8. Hippisley-Cox J, Coupland C, Vinogradova Y, Robson J, May M, Brindle P. Derivation and validation of QRISK, a new cardiovascular disease risk score for the United Kingdom: prospective open cohort study. *BMJ*. 2007;335:136.

9. Wilson PW, D'Agostino RB, Levy D, Belanger AM, Silbershatz H, Kannel WB. Prediction of coronary heart disease using risk factor categories. *Circulation*. 1998;97:1837-47.

10. Cooper JA, Miller GJ, Humphries SE. A comparison of the PROCAM and Framingham point-scoring systems for estimation of individual risk of coronary heart disease in the Second Northwick Park Heart Study. *Atherosclerosis*. 2005;181:93-100.

11. Miller GJ, Bauer KA, Barzegar S, Foley AJ, Mitchell JP, Cooper JA, Rosenberg RD. The effects of quality and timing of venepuncture on markers of blood coagulation in healthy middle-aged men. *Thromb Haemost*. 1995;73:82-6.

Circulation: Genomic and Precision Medicine

12. Bochenek G, Hasler R, El Mokhtari NE, Konig IR, Loos BG, Jepsen S, Rosenstiel P, Schreiber S, Schaefer AS. The large non-coding RNA ANRIL, which is associated with atherosclerosis, periodontitis and several forms of cancer, regulates ADIPOR1, VAMP3 and C11ORF10. *Hum Mol Genet*. 2013;22:4516-27.

13. Lo Sardo V, Chubukov P, Ferguson W, Kumar A, Teng EL, Duran M, Zhang L, Cost G, Engler AJ, Urnov F, et al. Unveiling the Role of the Most Impactful Cardiovascular Risk Locus through Haplotype Editing. *Cell*. 2018;175:1796-1810 e20.

14. O'Connor A, Brasher CJ, Slatter DA, Meckelmann SW, Hawksworth JI, Allen SM, O'Donnell VB. LipidFinder: A computational workflow for discovery of lipids identifies eicosanoid-phosphoinositides in platelets. *JCI Insight*. 2017;2:e91634.

15. Carvajal-Rodriguez A, de Una-Alvarez J, Rolan-Alvarez E. A new multitest correction (SGoF) that increases its statistical power when increasing the number of tests. *BMC Bioinformatics*. 2009;10:209.

16. Aoki J, Taira A, Takanezawa Y, Kishi Y, Hama K, Kishimoto T, Mizuno K, Saku K, Taguchi R, Arai H. Serum lysophosphatidic acid is produced through diverse phospholipase pathways. *J Biol Chem*. 2002;277:48737-44.

American Heart Association.

17. Wuensch KL. Comparing correlation coeffecients, slopes and intercepts. 2007.

Circulation: Genomic and Precision Medicine

18. Kurano M, Suzuki A, Inoue A, Tokuhara Y, Kano K, Matsumoto H, Igarashi K, Ohkawa R, Nakamura K, Dohi T, et al. Possible involvement of minor lysophospholipids in the increase in plasma lysophosphatidic acid in acute coronary syndrome. *Arterioscler Thromb Vasc Biol*. 2015;35:463-70.

19. Schober P, Boer C, Schwarte LA. Correlation Coefficients: Appropriate Use and Interpretation. *Anesth Analg*. 2018;126:1763-1768.

20. Kong Y, Hsieh CH, Alonso LC. ANRIL: A lncRNA at the CDKN2A/B Locus With Roles in Cancer and Metabolic Disease. *Front Endocrinol (Lausanne)*. 2018;9:405.

21. Alshehry ZH, Mundra PA, Barlow CK, Mellett NA, Wong G, McConville MJ, Simes J, Tonkin AM, Sullivan DR, Barnes EH, et al. Plasma Lipidomic Profiles Improve on Traditional Risk Factors for the Prediction of Cardiovascular Events in Type 2 Diabetes Mellitus. *Circulation*. 2016;134:1637-1650.

22. Cheng JM, Suoniemi M, Kardys I, Vihervaara T, de Boer SP, Akkerhuis KM, Sysi-Aho M, Ekroos K, Garcia-Garcia HM, Oemrawsingh RM, et al. Plasma concentrations of molecular lipid species in relation to coronary plaque characteristics and cardiovascular outcome: Results of the ATHEROREMO-IVUS study. *Atherosclerosis*. 2015;243:560-6.

23. Havulinna AS, Sysi-Aho M, Hilvo M, Kauhanen D, Hurme R, Ekroos K, Salomaa V, Laaksonen R. Circulating Ceramides Predict Cardiovascular Outcomes in the Population-Based FINRISK 2002 Cohort. *Arterioscler Thromb Vasc Biol*. 2016;36:2424-2430.

24. Hinterwirth H, Stegemann C, Mayr M. Lipidomics: quest for molecular lipid biomarkers in cardiovascular disease. *Circ Cardiovasc Genet*. 2014;7:941-54.

25. Holcapek M, Cervena B, Cifkova E, Lisa M, Chagovets V, Vostalova J, Bancirova M, Galuszka J, Hill M. Lipidomic analysis of plasma, erythrocytes and lipoprotein fractions of cardiovascular disease patients using UHPLC/MS, MALDI-MS and multivariate data analysis. *J Chromatogr B Analyt Technol Biomed Life Sci*. 2015;990:52-63.

26. Jove M, Naudi A, Portero-Otin M, Cabre R, Rovira-Llopis S, Banuls C, Rocha M, Hernandez-Mijares A, Victor VM, Pamplona R. Plasma lipidomics discloses metabolic syndrome with a specific HDL phenotype. *FASEB J*. 2014;28:5163-71.

27. Laaksonen R. Identifying new Risk Markers and Potential Targets for Coronary Artery Disease: The Value of the Lipidome and Metabolome. *Cardiovasc Drugs Ther*. 2016;30:19-32.

28. Lu J, Chen B, Chen T, Guo S, Xue X, Chen Q, Zhao M, Xia L, Zhu Z, Zheng L, et al. Comprehensive metabolomics identified lipid peroxidation as a prominent feature in human plasma of patients with coronary heart diseases. *Redox Biol*. 2017;12:899-907.

29. Meikle PJ, Wong G, Barlow CK, Kingwell BA. Lipidomics: potential role in risk prediction and therapeutic monitoring for diabetes and cardiovascular disease. *Pharmacol Ther*. 2014;143:12-23.

30. Rankin NJ, Preiss D, Welsh P, Sattar N. Applying metabolomics to cardiometabolic intervention studies and trials: past experiences and a roadmap for the future. *Int J Epidemiol*. 2016;45:1351-1371.

31. Stegemann C, Pechlaner R, Willeit P, Langley SR, Mangino M, Mayr U, Menni C, Moayyeri A, Santer P, Rungger G, et al. Lipidomics profiling and risk of cardiovascular disease in the prospective population-based Bruneck study. *Circulation*. 2014;129:1821-31.

32. Syme C, Czajkowski S, Shin J, Abrahamowicz M, Leonard G, Perron M, Richer L, Veillette S, Gaudet D, Strug L, et al. Glycerophosphocholine Metabolites and Cardiovascular Disease Risk Factors in Adolescents: A Cohort Study. *Circulation*. 2016;134:1629-1636.

33. Tsimikas S, Willeit P, Willeit J, Santer P, Mayr M, Xu Q, Mayr A, Witztum JL, Kiechl S. Oxidation-specific biomarkers, prospective 15-year cardiovascular and stroke outcomes, and net reclassification of cardiovascular events. *J Am Coll Cardiol*. 2012;60:2218-29.

34. Vorkas PA, Shalhoub J, Isaac G, Want EJ, Nicholson JK, Holmes E, Davies AH. Metabolic phenotyping of atherosclerotic plaques reveals latent associations between free cholesterol and ceramide metabolism in atherogenesis. *J Proteome Res*. 2015;14:1389-99.

American Heart Association.
Circulation: Genomic and Precision Medicine

35. Weir JM, Wong G, Barlow CK, Greeve MA, Kowalczyk A, Almasy L, Comuzzie AG, Mahaney MC, Jowett JB, Shaw J, et al. Plasma lipid profiling in a large population-based cohort. *J Lipid Res*. 2013;54:2898-908.

36. Li YF, Li RS, Samuel SB, Cueto R, Li XY, Wang H, Yang XF. Lysophospholipids and their G protein-coupled receptors in atherosclerosis. *Front Biosci (Landmark Ed)*. 2016;21:70-88.

37. Yang LV, Radu CG, Wang L, Riedinger M, Witte ON. Gi-independent macrophage chemotaxis to lysophosphatidylcholine via the immunoregulatory GPCR G2A. *Blood*. 2005;105:1127-34.

38. Aiyar N, Disa J, Ao Z, Ju H, Nerurkar S, Willette RN, Macphee CH, Johns DG, Douglas SA. Lysophosphatidylcholine induces inflammatory activation of human coronary artery smooth muscle cells. *Mol Cell Biochem*. 2007;295:113-20.

39. Matsumoto T, Kobayashi T, Kamata K. Role of lysophosphatidylcholine (LPC) in atherosclerosis. *Curr Med Chem*. 2007;14:3209-20.

40. Schmitz G, Ruebsaamen K. Metabolism and atherogenic disease association of lysophosphatidylcholine. *Atherosclerosis*. 2010;208:10-8.

41. Marathe GK, Pandit C, Lakshmikanth CL, Chaithra VH, Jacob SP, D'Souza CJ. To hydrolyze or not to hydrolyze: the dilemma of platelet-activating factor acetylhydrolase. *J Lipid Res*. 2014;55:1847-54.

42. Stafforini DM, Zimmerman GA. Unraveling the PAF-AH/Lp-PLA2 controversy. *J Lipid Res*. 2014;55:1811-4.

43. Law SH, Chan ML, Marathe GK, Parveen F, Chen CH, Ke LY. An Updated Review of Lysophosphatidylcholine Metabolism in Human Diseases. *Int J Mol Sci*. 2019;20.

44. Fernandez C, Sandin M, Sampaio JL, Almgren P, Narkiewicz K, Hoffmann M, Hedner T, Wahlstrand B, Simons K, Shevchenko A, et al. Plasma lipid composition and risk of developing cardiovascular disease. *PLoS One*. 2013;8:e71846.

45. Ganna A, Salihovic S, Sundstrom J, Broeckling CD, Hedman AK, Magnusson PK, Pedersen NL, Larsson A, Siegbahn A, Zilmer M, et al. Large-scale metabolomic profiling identifies novel biomarkers for incident coronary heart disease. *PLoS Genet*. 2014;10:e1004801.

46. Zollbrecht C, Grassl M, Fenk S, Hocherl R, Hubauer U, Reinhard W, Esslinger UB, Ebert S, Langmann T, Stark K, et al. Expression pattern in human macrophages dependent on 9p21.3 coronary artery disease risk locus. *Atherosclerosis*. 2013;227:244-9.

American Heart Association.
Circulation: Genomic and Precision Medicine

47. Smyth SS, Mueller P, Yang F, Brandon JA, Morris AJ. Arguing the case for the autotaxin-lysophosphatidic acid-lipid phosphate phosphatase 3-signaling nexus in the development and complications of atherosclerosis. *Arterioscler Thromb Vasc Biol*. 2014;34:479-86.

48. Samani NJ, Schunkert H. Chromosome 9p21 and cardiovascular disease: the story unfolds. *Circ Cardiovasc Genet*. 2008;1:81-4.

49. Teslovich TM, Musunuru K, Smith AV, Edmondson AC, Stylianou IM, Koseki M, Pirruccello JP, Ripatti S, Chasman DI, Willer CJ, et al. Biological, clinical and population relevance of 95 loci for blood lipids. *Nature*. 2010;466:707-13.

50. Wootton PT, Drenos F, Cooper JA, Thompson SR, Stephens JW, Hurt-Camejo E, Wiklund O, Humphries SE, Talmud PJ. Tagging-SNP haplotype analysis of the secretory PLA2IIa gene PLA2G2A shows strong association with serum levels of sPLA2IIa: results from the UDACS study. *Hum Mol Genet*. 2006;15:355-61.

51. Scaglia N, Tyekucheva S, Zadra G, Photopoulos C, Loda M. De novo fatty acid synthesis at the mitotic exit is required to complete cellular division. *Cell Cycle*. 2014;13:859-68.

52. Yao XL, Lu XL, Yan CY, Wan QL, Cheng GC, Li YM. Circulating miR-122-5p as a potential novel biomarker for diagnosis of acute myocardial infarction. *Int J Clin Exp Pathol*. 2015;8:16014-9.

American Heart Association.
Circulation: Genomic and Precision Medicine

Table 1: Number of detected and identified lipid features in the global lipidomics assay. The number of lipids in each class are shown, with the number of significantly different lipids (non-parametric one-tailed Mann–Whitney U test, assuming unequal variance with a threshold of $p \leq 0.05$, after SGoF correction), between the SNP group and controls shown in parentheses.

**Lipid Class**	**FA**	**GPL**	**GL**	**SL**	**Sterol**	**Prenol**	**Unknowns**	**TOTAL**
Detected (P<0.05 after SGoF)	51 (0)	220 (0)	401 (13)	166 (2)	27 (1)	7 (0)	1006 (1)	1878 (17)

FA: Fatty acyl, GPL: glycerophospholipid, GL: glycerolipid, SL: sphingolipid

American Heart Association.
Circulation: Genomic and Precision Medicine

Table 2. Several lipid related Gene Ontology Pathways are significantly regulated by ANRIL silencing in HEK 293 cells. Results are from the PANTHER Over-representation Test, Term enrichment service (pantherdb.org), using default analysis parameters (Fisher's Exact test with False Discovery Rate: FDR P < 0.05, Benjamini-Hochberg). Fold-enrichment represents the number of observed differentially expressed genes with the GO annotation of interest, relative to genome background. The full list of genes significantly altered in these GO processes, is provided in Supplementary Data.xlsx, along with a list of all significantly altered GO processes at both timepoints. Note that FDR has no unit, while fold enrichment is a ratio with no unit.

	GO biological process	Number of significantly different genes	GO term fold enrichment	P-value	FDR
**48 hrs shRNA knockdown versus control**	glycosphingolipid metabolic process (GO:0006687)	14	3.05	5.68E-04	3.12E-02
	regulation of lipid metabolic process (GO:0019216)	46	1.9	1.12E-04	9.22E-03
	phospholipid metabolic process (GO:0006644)	51	1.85	9.16E-05	7.99E-03
	cellular lipid metabolic process (GO:0044255)	109	1.68	6.07E-07	1.37E-04
	lipid metabolic process (GO:0006629)	130	1.61	5.02E-07	1.19E-04
	response to lipid (GO:0033993)	84	1.53	2.59E-04	1.74E-02
**96 hrs shRNA knockdown versus control**	regulation of lipid metabolic process (GO:0019216)	74	1.88	2.80E-06	3.38E-04
	phospholipid metabolic process (GO:0006644)	73	1.63	2.05E-04	1.30E-02
	lipid biosynthetic process (GO:0008610)	102	1.61	1.54E-05	1.51E-03
	cellular lipid metabolic process (GO:0044255)	164	1.56	2.74E-07	4.70E-05
	lipid metabolic process (GO:0006629)	203	1.54	1.61E-08	3.79E-06

American Heart Association
Circulation: Genomic and Precision Medicine

Table 3. Several LysoPL relevant genes are significantly altered in ANRIL knockdown. Data were analysed using the oligo and limma packages in Bioconductor, see methods. P-values were corrected for multiple testing using Benjamini-Hochberg (adjusted p-value cut-off: 0.05), note there are no units for P-values, and log2fold change is a ratio.

**Predicted effect on lysoPL(↓) on lysoPA(↓)**		**48 hr, knockdown vs control**		**96 hr, knockdown vs control**	
	**Gene name**	**Log2fold change in gene expression**	**adjustedPval**	**Log2fold change in gene expression**	**adjustedPval**
↓↑	*ENPP2*	1.249	7.50E-3	1.483	4.65E-05
↓	*LPCAT2*	0.212	1.95E-02	NS	NS
↓	*MBOAT2*	NS	NS	0.289	3.28E-03
↓	*ACSL6*	0.283	0.0017	0.519	5.31E-06
↓	*PLA2G4C*	-0.351	0.0266	-0.356	0.016
↓	*PNPLA2*	-0.447	0.000031	-0.666	2.53E-7
↓	*PLBD1*	0.368	0.0263	0.5036	0.00217
↓	*PLPP1*	NS	NS	0.1637	0.049
↓	*PLPP2*	0.258	0.00137	0.2940	4.65E-5
↓	*PLPPR2*	0.1136	0.0792	0.124	0.0101
↑	*PLA2G7*	0.611	0.000225	0.439	0.0016
↑	*LPCAT1*	-0.257	0.025	NS	NS
↑	*DGKA*	0.247	0.00724	0.403	9.98E-5
↑	*LPL*	NS	NS	0.230	0.0429
↑	*LPCAT3*	NS	NS	-0.298	0.0013

American Heart Association.
Circulation: Genomic and Precision Medicine

Figure Legends:

Figure 1. Global lipidomics reveals class specific changes in GPLs in rs10757274 GG vs AA. *Panel A: Scatterplot of features in a plasma sample (≈14,000) after processing high-resolution MS data using XCMS.* Analysis was undertaken using parameters provided in Supplemental Material. *Panel B. Scatterplot obtained after LipidFinder and manual-data clean-up, as described in Methods*. Each dot represents a lipid described by *m/z* value and retention time. Putative identification and assignment of category was performed using WebSearch of the curated LIPID MAPS database. *Panels C-J. Volcano plots show differences in lipid classes with genotype.* Volcano plots were generated as described in Methods, plotting log2(fold-change) versus –log10(p-value) for all (n = 39 AA, 33 GG), following p-value adjustment using sequential goodness of fit metatest (SGoF).

Circulation: Genomic and Precision Medicine

Figure 2. LysoPLs are significantly reduced in rs10757274 GG, but not in subjects with unrelated SNPs. *Panel A. Several LPCs are lower in GG samples than AA controls, and LPEs trend towards lower levels.* LysoPLs were determined using LC/MS/MS as described in Methods (n = 88 AA, 81 GG). Tukey box plot, * $p < 0.05$, ** $p < 0.01$, *** $p < 0.005$, 2-tailed, unpaired Student's T-test (black) and Mann Whitney U (red). *Panel B. Plasma lysoPL are not altered by other risk SNPs.* Plasma from the NPHSII cohort containing several risk (up or down) SNPs were analysed using LipidFinder, and *m/z* values corresponding to lysoPL extracted and compared. These are plotted on a volcano plot, to show fold change *vs* significance, following p-value adjustment using sequential goodness of fit metatest (SGoF). Numbers and genotypes are shown in Supplementary Table 1. *Panel C. ATX is significantly decreased in GG samples*

compared to AA controls. Plasma ATX activity was measured as described in Methods (n = 47 AA, 49 GG). *Panel D. LysoPAs are significantly decreased in GG plasma compared to AA controls.* Plasma lysoPAs were measured as described in Methods, using LC/MS/MS (n = 95 AA, 100 GG). Tukey box plot, * p < 0.05, ** p < 0.01, *** p < 0.005, 2-tailed, unpaired Student's T-test (black).

Figure 3. The lysoPL/lysoPA/ATX axis is dysregulated in the GG plasmas, while the profile of molecular species is unchanged for lysoPL/lysoPA. *Panels A-D. ATX shows altered correlations with plasma lysoPL or lysoPA in GG versus AA plasma*. Levels of lysoPL or lysoPA quantified by LC/MS/MS in the validation cohort were correlated using Answerminer, to determine Pearson's correlation co-efficient. *A*,*B*: AA control plasma, *C*,*D*: GG risk plasma (n = 47 AA, 49 GG). *Panels E-I. LysoPL and lysoPA are positively correlated for AA plasma, but negatively correlated for GG*. The sum of all lysoPAs or lysoPLs in each set were correlated using Answerminer, as above (E,G). Alternatively, lipids containing 18:2, or 20:4 were separately correlated (F,H,I). *E,F*: AA control plasma, G,H,I: GG risk plasma (n = 47 AA, 49 GG). *Panels J,K. The lysoPA(18:2)/lysoPL(18:2) ratio positively correlates with ATX in AA plasma, but negatively for GG, indicating a block in substrate:product conversion in GG*. Correlations were performed using Answerminer (n = 47 AA, 49 GG). *J*: AA plasma, *K*: GG plasma. P<0.05 indicates significant using Pearson's correlation test. *Panel L. The profile of individual lysoPL or lysoPA molecular species is unchanged between GG and AA plasmas*. Levels of individual lysoPL/lysoPA were compared across both groups, and shown as %.

American Heart Association.
Circulation: Genomic and Precision Medicine

Figure 4. Cytoscape analysis of lipids reveals divergent metabolism in GG *versus* AA, while *ANRIL* knockdown is associated with significant changes to lysoPL/lysoPA-metabolising genes. *Panel A. Cytoscape reveals strong links within related families, but a positive-negative switch for lysoPL-lysoPA correlations between AA-GG plasmas.* Pearson correlation networks were generated for the AA and GG validation samples (n = 47 AA, 49 GG), using lipid concentrations. Nodes are coloured by lipid sub-category and represent individual molecular species, and edges represent the correlation. Edges detail the Pearson correlation coefficients between nodes (lipids), where the width of the edge denotes value. Additionally, edges are coloured by value: red (r = 0.10-0.39); green (r = 0.40-0.69); grey (r 0.70-1.00). *Panels C,D. Significant changes in lipid regulatory gene expression are observed with ANRIL knockdown in cell culture.* Affymetrix array data generated in [5] was analysed using GO as described in Methods. Volcano plot showing differential gene expression of all genes on the Affymetrix HuGene1.0 v1, chip. LysoPL/lysoPA regulating genes that alter in line with decreased levels of the lipids in GG plasma are labelled. The horizontal dashed line shows where adj.pvalue < 0.05 (Benjamini-Hochberg correction) where points (genes) above this line are significantly differentially expressed. LysoPL-regulating genes that alter in line with decreased levels of the lipids are labelled in black. Genes in red are annotated to the GO-term detailed in the plot title. Data are plotted in R using ggplot2. *Panel B*: 48-hr shRNA knockdown, *Panel C*: 96-hr shRNA knockdown.

American Heart Association.
Circulation: Genomic and Precision Medicine

Figure 5. VSMCs from risk haplotypes show differential gene expression of lysoPL metabolizing genes, that are rescued by deletion of the Chr9p21 locus. *Panel A. PCA shows that the presence of risk haplotypes is associated with differential gene expression of lysoPL genes.*

iPSCs from peripheral monocytes were obtained and differentiated as described in Supplemental Material. RNAseq data was clustered using lysoPL metabolizing genes by PCA in R. Non-risk haplotype (NNWT), risk haplotype (RRWT) and their genome edited counterparts (NNKO and RRKO) are shown. *Panel B. Example datasets for ACSL3 and DGKA, showing that removing the risk locus reverts gene expression back to levels in non-risk individuals*. * $p < 0.05$, ** $p < 0.01$, *** $p < 0.005$, Students t-test, n = 9-10 clones per group. *Panels C,D. Schematics showing impact of ANRIL silencing or risk haplotypes on relevant lysoPL metabolizing genes.*

Figure 6. Metabolic pathway showing lysoPL/lysoPA regulatory genes that are significantly-altered by ANRIL knockdown in HEK cells. Genes that metabolise these lipids are shown. Full data on their transcriptional regulation is provided in Table 3, Supplementary data.xls tab 7. LCAT was not significantly regulated but is shown for completeness.

American Heart Association.
Circulation: Genomic and Precision Medicine

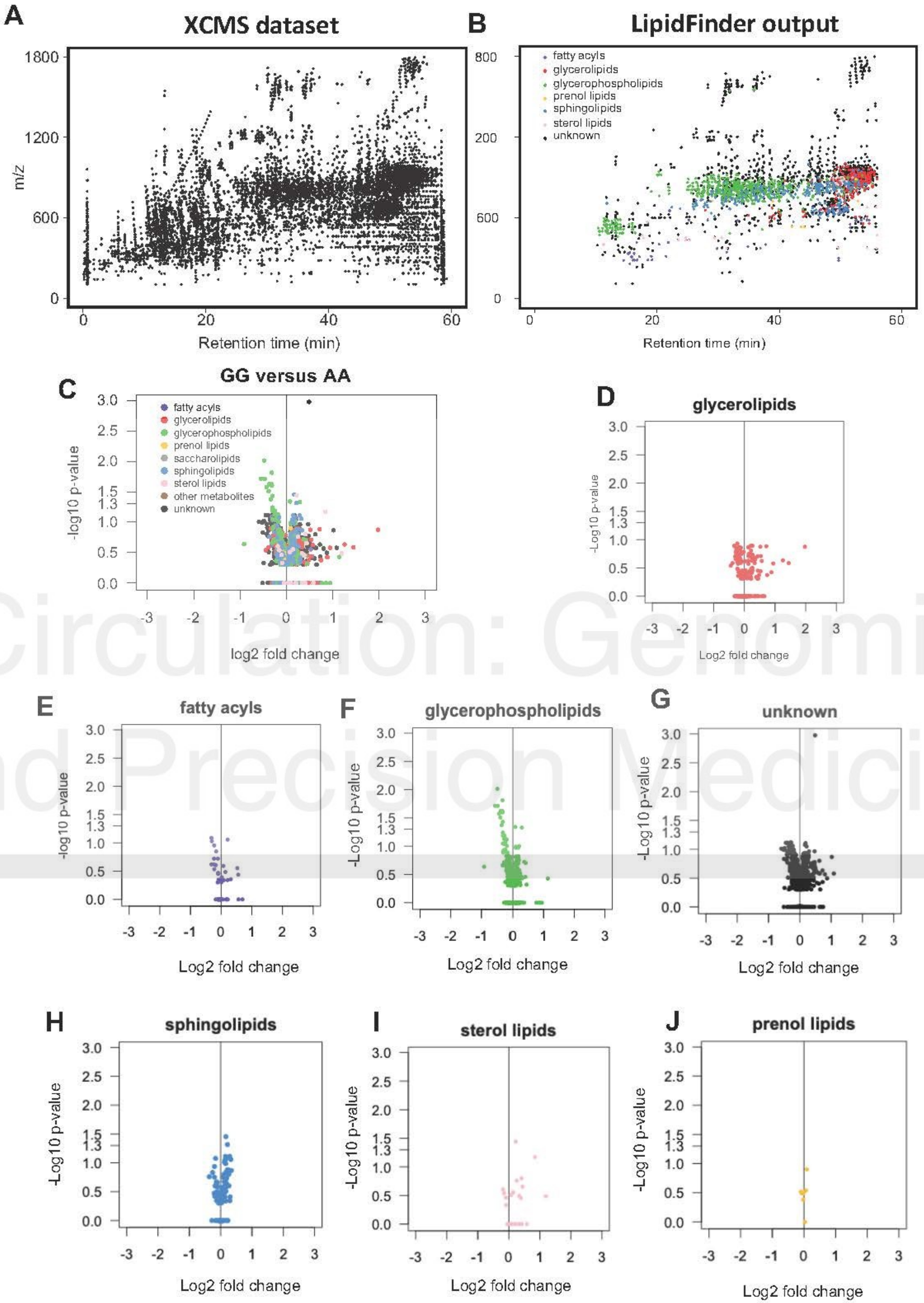

American Heart Association.
Circulation: Genomic and Precision Medicine

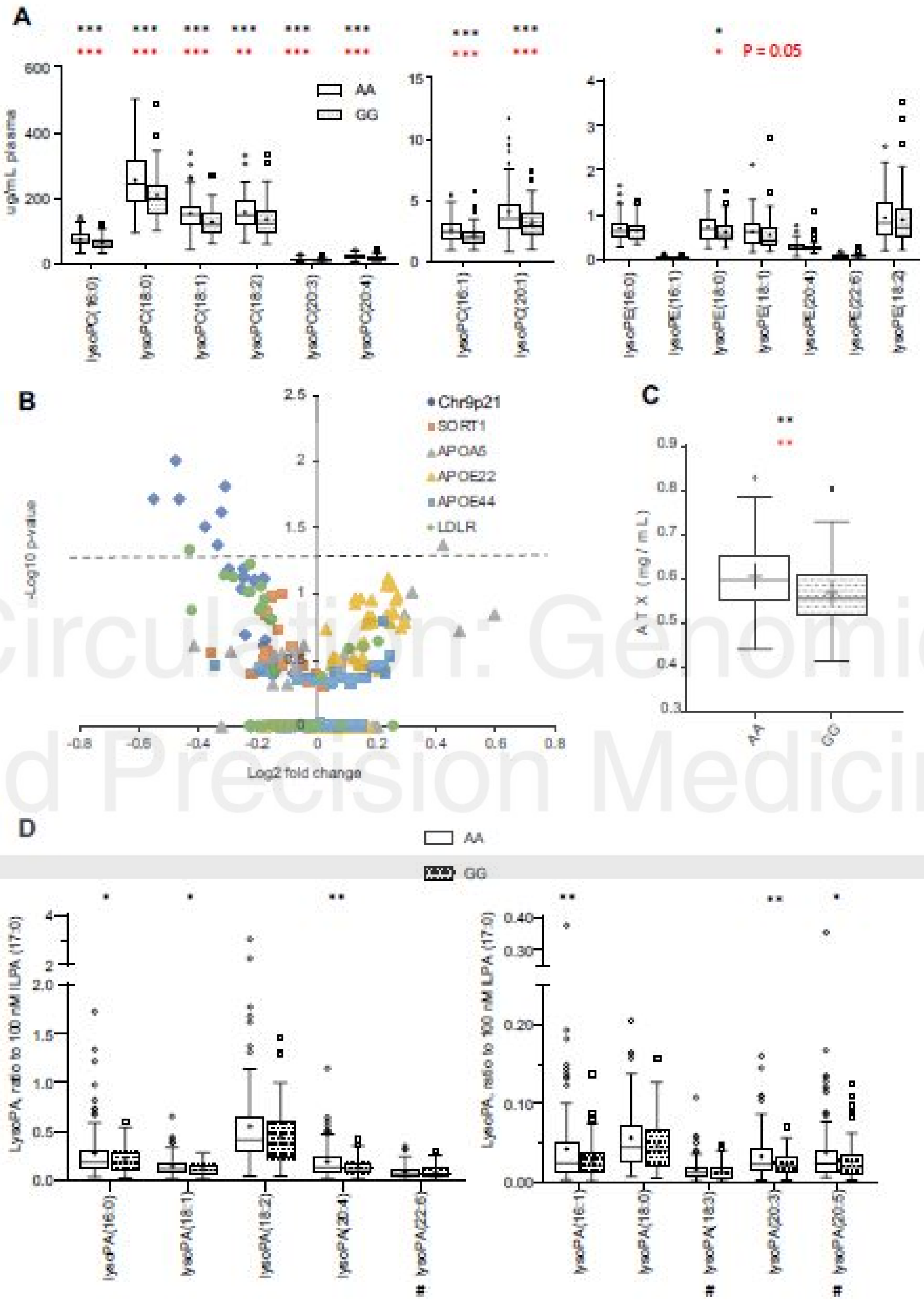

Circulation: Genomic and Precision Medicine
American Heart Association.

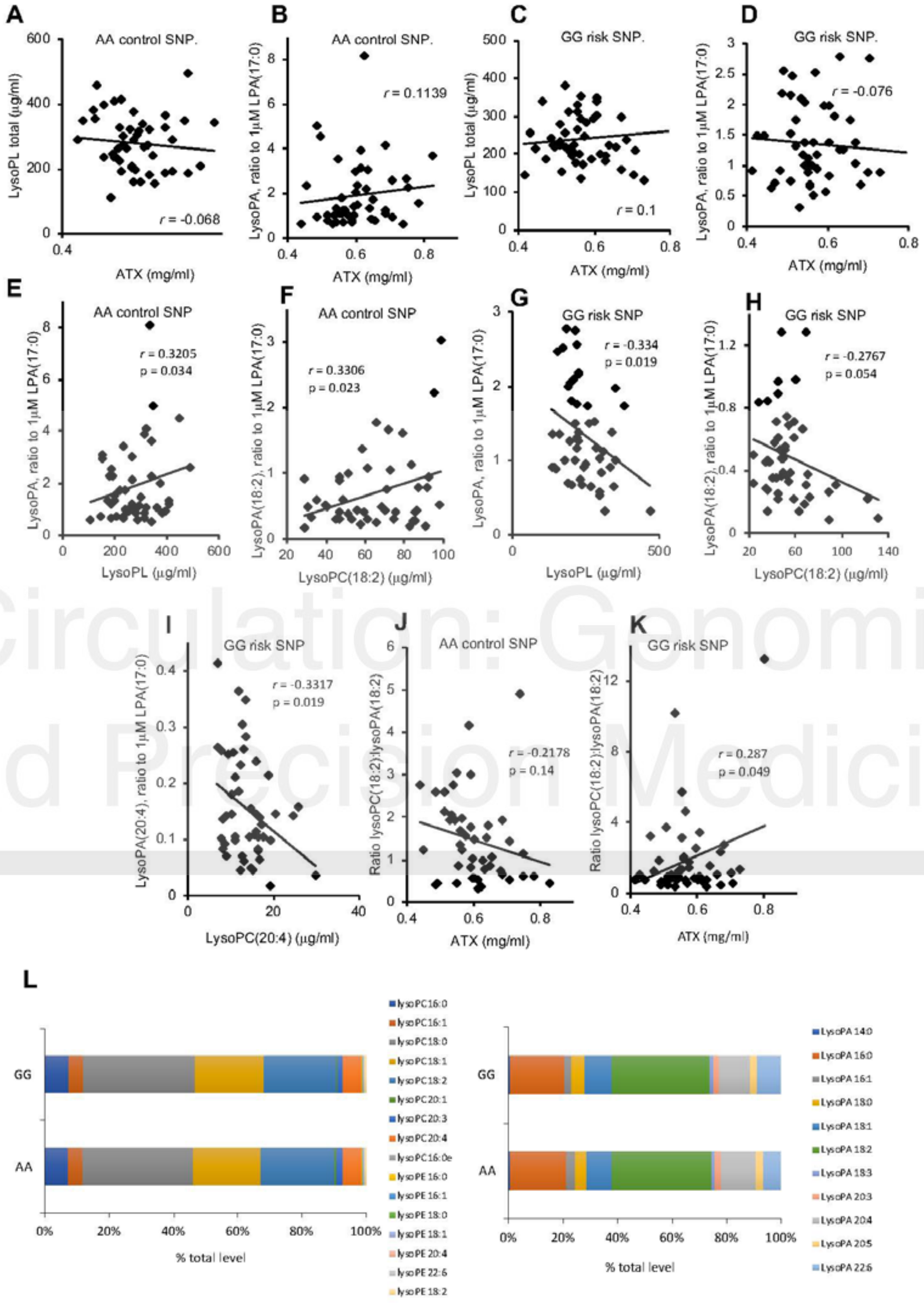

Circulation: Genomic and Precision Medicine
American Heart Association.

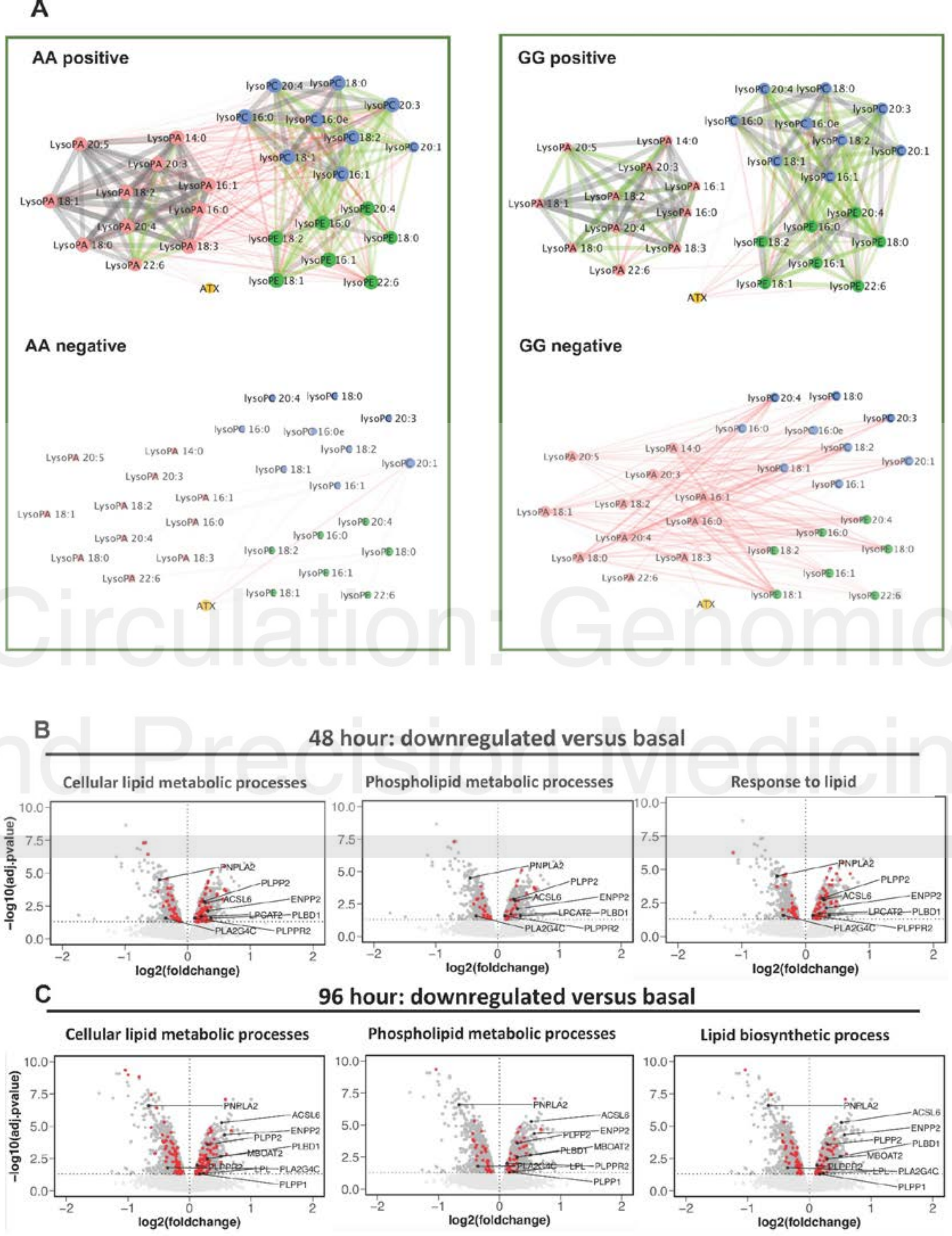

Circulation: Genomic and Precision Medicine
American Heart Association.

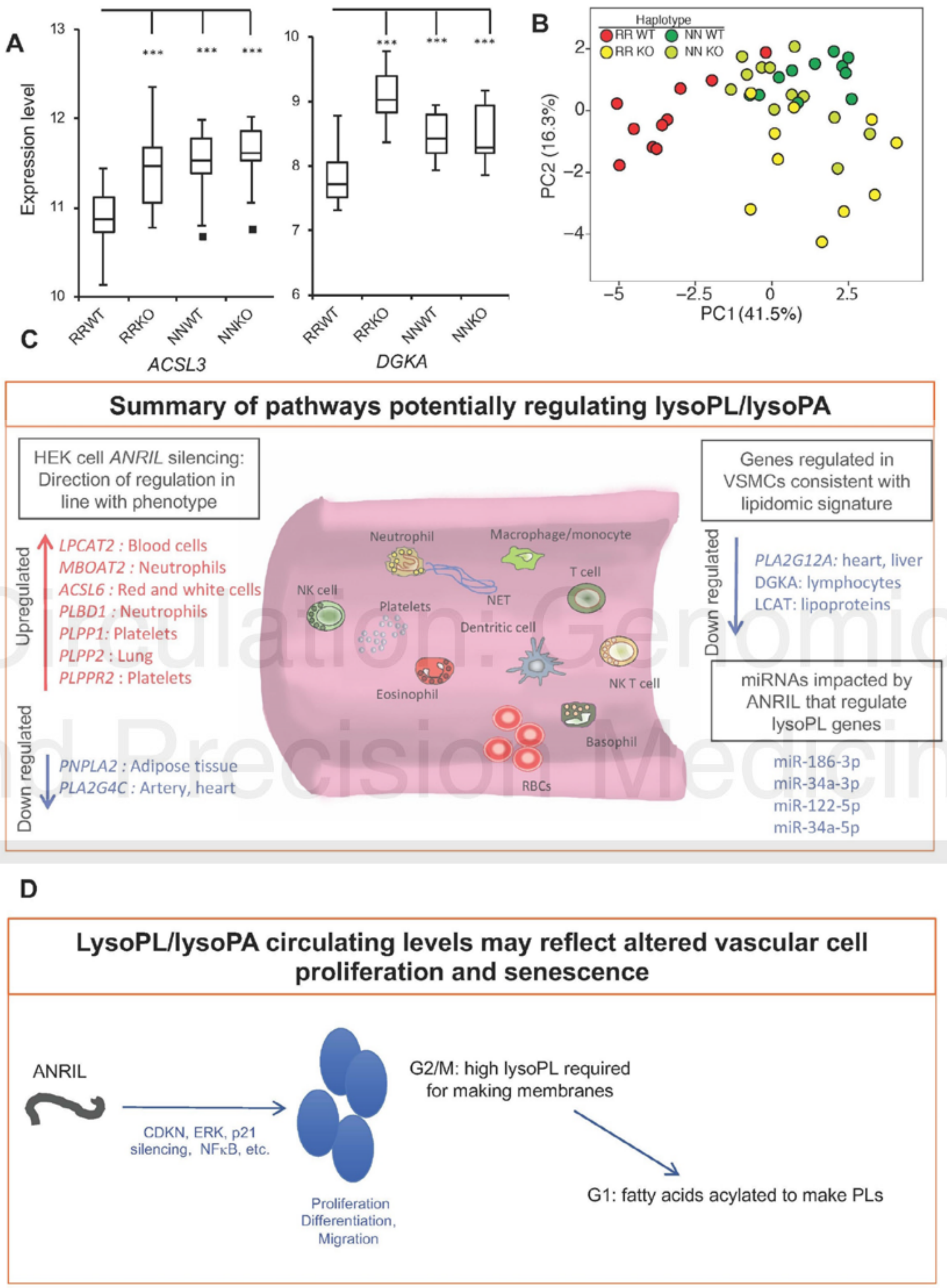

Circulation: Genomic and Precision Medicine
American Heart Association.

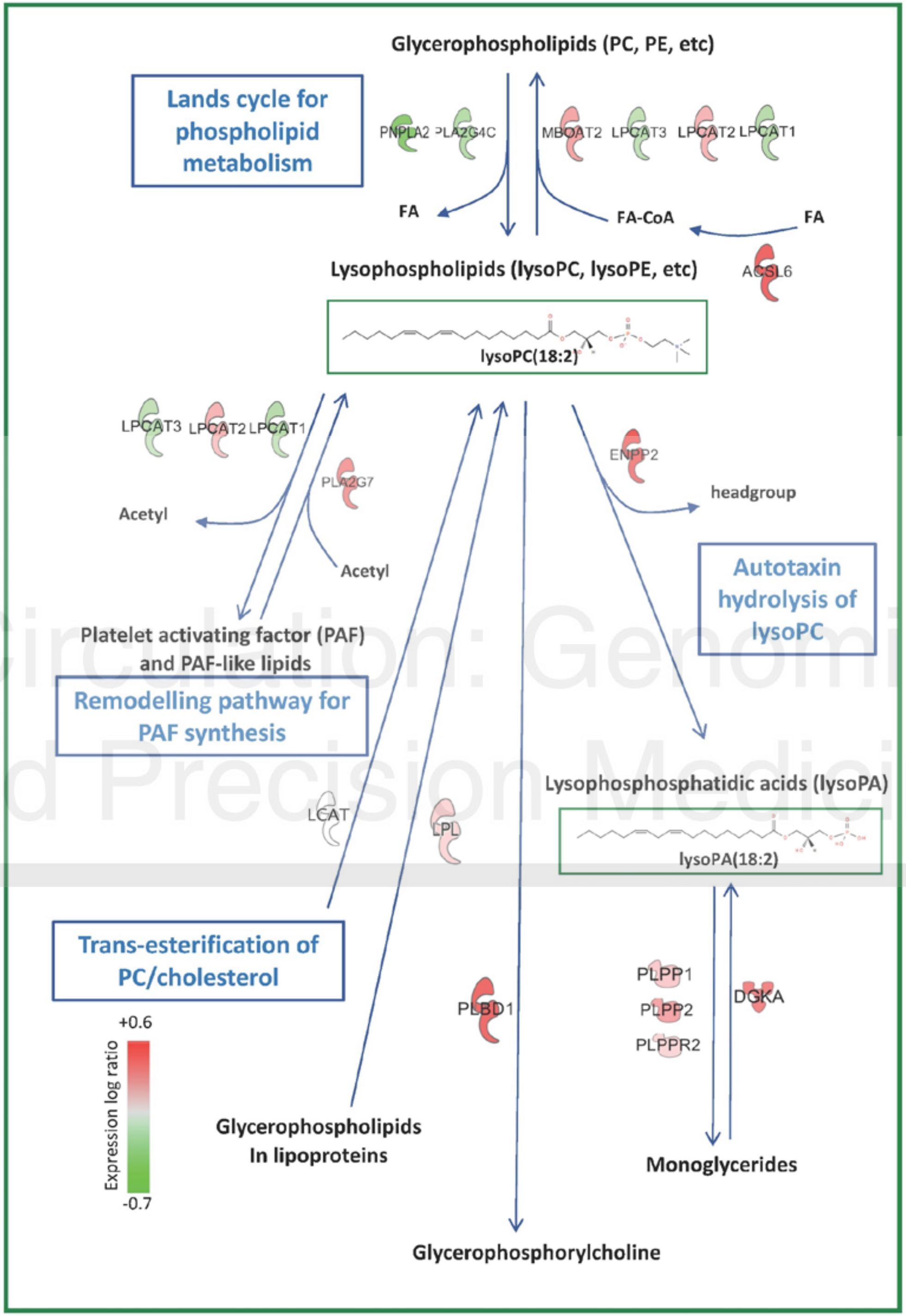

Circulation: Genomic and Precision Medicine
American Heart Association.
